# Supplementary material for: Duplication of NRAMP3 Gene in Poplars Generated Two Homologous Transporters with Distinct Functions
Source: Mol Biol Evol. 2022 Jun 14;39(6):msac129. doi: 10.1093/molbev/msac129 (PMC9234761; doi:10.1093/molbev/msac129)
Supplement: msac129_Supplementary_Data [file msac129_supplementary_data.zip › Pottier,_Le_Thi_et_al_SI.pdf]

Supplementary Material

Duplication of NRAMP3 gene in poplars generated two homologous transporters with distinct functions

*Mathieu Pottier[1,3], Van Anh Le Thi[1,4], Catherine Primard-Brisset[1], Jessica Marion[1], Michele Bianchi[1], Cindy Victor[1], Annabelle Déjardin[2], Gilles Pilate[2], Sébastien Thomine[1]**

[1]Institut de Biologie Intégrative de la Cellule, CNRS, Avenue de la Terrasse, 91198 Gif-sur-Yvette, France

[2]INRAE, ONF, BioForA, Orléans, France

[3]Current affiliation: Institute for Molecular Physiology, Heinrich-Heine-University Düsseldorf, Düsseldorf 40225, Germany

[4]Current affiliation: Graduate University of Science and Technology (GUST), Vietnam Academy of Science and Technology (VAST), 18 Hoang Quoc Viet, Cau Giay, Hanoi 10000, Vietnam

*Author for correspondence

Sébastien Thomine

Sebastien.thomine@i2bc.paris-saclay.fr

Supplementary Tables

Supplementary table S1. List of the species and corresponding resources exploited in this work. Sequences were retrieved from the Phytozome V13 (https://phytozome-next.jgi.doe.gov/) (Goodstein et al. 2012), PopGenIE V3 (https://PopGenIE.org/) (Sjödin et al. 2009), NCBI (https://www.ncbi.nlm.nih.gov/) (Sayers et al. 2021), and 1KP Project (https://db.cngb.org/onekp/) (One Thousand Plant Transcriptomes Initiative 2019) databases.

Species	Section (poplar)	Resource	Database	Reference
Arabidopsis thaliana		Genome TAIR 10	TAIR	**(Lamesch et al. 2012)**
Populus trichocarpa	Tacahamaca	Genome V4.1	Phytozome V13	(Tuskan et al. 2006)
Populus tremula	Populus	Genome V1.0	PopGenIE V3	(Lin et al. 2018)
Populus tremuloides	Populus	Genome V1.1	PopGenIE V3	
Populus grandidentata	Populus	Genome <V0.99	PopGenIE V3	UPSC Genomes Archive
Populus alba	Populus	SRA: SRR9007070	NCBI	(Zhang et al. 2019)
Populus cathayana	Tacahamaca	SRA: SRR9007071	NCBI	
Populus simonii	Tacahamaca	SRA: SRR9007072	NCBI	
Populus lasiocarpa	Leucoides	SRA: SRR9007073	NCBI	
Populus maximowiczii	Tacahamaca	SRA: SRR9007074	NCBI	
Populus euphratica	Turanga	SRA: SRR9007075	NCBI	
Populus ussuriensis	Tacahamaca	SRA: SRR9007077	NCBI	
Populus nigra	Aigeiros	SRA: SRR9007078	NCBI	
Populus deltoides	Aigeiros	SRA: SRR9007079	NCBI	
Populus mexicana	Abaso	SRA:<br>-SRR11580850<br>-SRR11580851	NCBI	
Salix purpurea		Genome V5.0	Phytozome 13	(Zhou et al. 2018)
Salix suchowensis		Genome	NCBI	(Dai et al. 2014)
Salix brachista		Genome	NCBI	(Chen et al. 2019)
Salix dasyclados		Transcriptome	1KP Project	(One Thousand Plant Transcriptomes Initiative 2019)
Salix fargesii		Transcriptome	1KP Project	
Salix eriocephala		Transcriptome	1KP Project	
Salix sachalinensis		Transcriptome	1KP Project	
Salix viminalis		Transcriptome	1KP Project	

Supplementary table S2. Sites under positive and purifying selection in *Populus NRAMP3.1* according to FEL method. Sites subjected to positive or purifying selections were estimated by the FEL method (https://www.datamonkey.org/)(Kosakovsky Pond and Frost 2005). *NRAMP3.1* coding sequences of 13 *Populus* species retrieved as indicated in Materials and Methods were used to generate a phylogenetic tree. A subset of branches encompassing the *NRAMP3.1s* were analyzed separately to estimate the synonymous substitution rate (dS) at a site (α) and the non-synonymous substitution rate (dN) at a site (β), and a maximum-likelihood approach was undertaken to calculate dN/dS rates for each codon site (ϖ). Amino-acids correspond to that of the consensus *Populus* NRAMP3.1 sequence. Dots indicate less than 90% of conservation within all investigated *Populus* NRAMP3.1 sequences. Sites subjected to positive selection are highlighted in dark ($p < 0.05$) and light green ($p < 0.1$). Sites subject to purifying selection are highlighted in dark ($p < 0.05$) and light red ($p < 0.1$). α=β : the rate estimated under the neutral model; LRT: likelihood ratio test.

Site	amino-acid	α	β	ϖ	α=β	LRT	p-value
1	M	0	0	No value	0	0	1
2	P	3.207	0	0	0.768	2.754	0.097
3	.	0	9.05	Infinity	3.725	4.756	0.029
4	P	0	0	No value	0	0	1
5	E	0	0	No value	0	0	1
6	E	0	0	No value	0	0	1
7	D	0	1.063	Infinity	0.736	0.728	0.394
8	P	5.82	1.186	0.204	2.494	1.579	0.209
9	.	0	2.809	Infinity	1.934	1.437	0.231
10	P	3.162	0	0	0.78	2.691	0.101
11	L	0	0	No value	0	0	1
12	L	2.026	0	0	1.044	1.319	0.251
13	K	0	2.603	Infinity	1.858	1.334	0.248
14	D	0	1.112	Infinity	0.84	0.563	0.453
15	Q	0	1.448	Infinity	0.991	0.754	0.385
16	E	0	0	No value	0	0	1
17	E	0	0	No value	0	0	1
18	T	0	1.3	Infinity	0.789	0.981	0.322
19	A	0	0	No value	0	0	1
20	Y	0	1.675	Infinity	1.262	0.556	0.456
21	D	0	0	No value	0	0	1
22	S	0	0	No value	0	0	1
23	D	3.393	1.134	0.334	1.741	0.509	0.476
24	G	0	1.378	Infinity	0.844	0.966	0.326
25	K	0	0	No value	0	0	1
26	V	4.189	0	0	1.879	3.269	0.071
27	L	2.005	0	0	0.801	1.819	0.177
28	S	2.724	4.033	1.481	3.495	0.095	0.758
29	F	0	1.965	Infinity	1.33	0.75	0.386
30	G	1.966	0	0	0.788	1.793	0.181
31	I	3.598	1.437	0.399	2.046	0.37	0.543
32	D	0	0	No value	0	0	1
33	Y	0	1.787	Infinity	1.199	0.771	0.38
34	D	0	1.094	Infinity	0.747	0.75	0.386
35	T	9.333	0	0	3.15	8.605	0.003
36	E	0	0	No value	0	0	1
37	S	2.649	0	0	0.699	2.605	0.107

38	G	0	0	No value	0	0	1
39	G	0	1.397	Infinity	0.689	1.361	0.243
40	S	0	0	No value	0	0	1
41	T	0	0	No value	0	0	1
42	V	0	1.693	Infinity	0.865	1.288	0.256
43	V	0	2.052	Infinity	0.981	1.442	0.23
44	P	1.903	1.077	0.566	1.374	0.157	0.692
45	S	2.32	1.47	0.634	1.826	0.094	0.759
46	F	0	0	No value	0	0	1
47	S	0	0	No value	0	0	1
48	W	0	0	No value	0	0	1
49	R	0	1.342	Infinity	0.897	0.767	0.381
50	K	4.546	0	0	0.898	3.182	0.074
51	L	0	0	No value	0	0	1
52	W	0	0	No value	0	0	1
53	L	2.183	0	0	1.044	1.408	0.235
54	F	5.723	0	0	1.224	3.249	0.071
55	T	0	0	No value	0	0	1
56	G	0	0	No value	0	0	1
57	P	0	0	No value	0	0	1
58	G	1.949	0	0	0.784	1.781	0.182
59	F	0	0	No value	0	0	1
60	L	0	0	No value	0	0	1
61	M	0	0	No value	0	0	1
62	C	1.704	1.575	0.924	1.636	0.003	0.96
63	I	0	0	No value	0	0	1
64	A	3.044	0	0	0.861	2.566	0.109
65	F	0	0	No value	0	0	1
66	L	0	0	No value	0	0	1
67	D	3.407	0	0	0.837	2.77	0.096
68	P	0	0	No value	0	0	1
69	G	6.296	0	0	2.577	5.407	0.02
70	N	0	0	No value	0	0	1
71	L	0	0	No value	0	0	1
72	E	0	0	No value	0	0	1
73	G	0	0	No value	0	0	1
74	D	0	0	No value	0	0	1
75	L	0	0	No value	0	0	1
76	Q	0	0	No value	0	0	1
77	A	0	0	No value	0	0	1
78	G	0	0	No value	0	0	1
79	A	1.979	0	0	0.727	1.943	0.163
80	I	2.45	0	0	0.853	2.105	0.147
81	A	1.942	0	0	0.725	1.931	0.165
82	G	6.48	0	0	2.625	5.495	0.019
83	Y	3.553	0	0	1.106	2.295	0.13
84	S	0	0	No value	0	0	1
85	L	0	0	No value	0	0	1
86	L	0	0	No value	0	0	1

87	W	0	0	No value	0	0	1
88	L	0	0	No value	0	0	1
89	L	1.929	0	0	0.789	1.774	0.183
90	L	0	0	No value	0	0	1
91	W	0	0	No value	0	0	1
92	A	0	0	No value	0	0	1
93	T	0	0	No value	0	0	1
94	A	0	0	No value	0	0	1
95	M	0	0	No value	0	0	1
96	G	6.514	0	0	1.995	4.826	0.028
97	L	0	0	No value	0	0	1
98	L	0	0	No value	0	0	1
99	V	0	0	No value	0	0	1
100	Q	0	0	No value	0	0	1
101	L	0	0	No value	0	0	1
102	L	1.996	0	0	0.933	1.52	0.218
103	S	0	0	No value	0	0	1
104	A	3.973	0	0	1.503	3.862	0.049
105	R	0	0	No value	0	0	1
106	L	0	0	No value	0	0	1
107	G	1.927	0	0	0.817	1.663	0.197
108	V	0	0	No value	0	0	1
109	A	0	0	No value	0	0	1
110	T	0	0	No value	0	0	1
111	G	3.292	0	0	1.156	2.579	0.108
112	R	2.398	0	0	0.75	2.241	0.134
113	H	0	0	No value	0	0	1
114	L	4.346	0	0	2.169	2.687	0.101
115	A	0	0	No value	0	0	1
116	E	0	0	No value	0	0	1
117	L	1.165	0	0	0.726	0.948	0.33
118	C	0	1.503	Infinity	1.192	0.462	0.496
119	R	0	0	No value	0	0	1
120	E	0	0	No value	0	0	1
121	E	0	0	No value	0	0	1
122	Y	0	0	No value	0	0	1
123	P	0	0	No value	0	0	1
124	T	4.515	0	0	1.426	4.283	0.038
125	W	0	0	No value	0	0	1
126	A	3.245	0	0	0.85	2.575	0.109
127	R	0	4.487	Infinity	1.927	3.294	0.07
128	M	0	0	No value	0	0	1
129	I	0	1.419	Infinity	0.968	0.732	0.392
130	L	0	0	No value	0	0	1
131	W	0	0	No value	0	0	1
132	I	0	0	No value	0	0	1
133	M	0	0	No value	0	0	1
134	A	0	0	No value	0	0	1
135	E	0	0	No value	0	0	1

136	L	0	0	No value	0	0	1
137	A	0	0	No value	0	0	1
138	L	0	0	No value	0	0	1
139	I	0	0	No value	0	0	1
140	G	0	0	No value	0	0	1
141	A	0	0	No value	0	0	1
142	D	0	0	No value	0	0	1
143	I	0	0	No value	0	0	1
144	Q	0	0	No value	0	0	1
145	E	3.161	0	0	0.885	2.509	0.113
146	V	0	0	No value	0	0	1
147	I	0	0	No value	0	0	1
148	G	1.908	0	0	0.792	1.741	0.187
149	S	0	0	No value	0	0	1
150	A	0	0	No value	0	0	1
151	I	14.276	0	0	2.316	7.011	0.008
152	A	3.118	0	0	0.844	2.533	0.111
153	I	0	0	No value	0	0	1
154	Q	0	1.412	Infinity	0.943	0.793	0.373
155	I	2.45	0	0	0.853	2.105	0.147
156	L	0	0	No value	0	0	1
157	S	0	0	No value	0	0	1
158	N	0	0	No value	0	0	1
159	G	0	0	No value	0	0	1
160	V	0	2.022	Infinity	1.288	0.882	0.348
161	L	0	0	No value	0	0	1
162	P	0	0	No value	0	0	1
163	L	2.038	0	0	1.01	1.345	0.246
164	W	0	0	No value	0	0	1
165	A	0	0	No value	0	0	1
166	G	0	0	No value	0	0	1
167	V	0	0	No value	0	0	1
168	I	0	1.297	Infinity	0.916	0.684	0.408
169	I	0	0	No value	0	0	1
170	T	0	0	No value	0	0	1
171	A	3.028	0	0	0.836	2.487	0.115
172	S	4.936	3.611	0.732	4.175	0.092	0.762
173	D	0	0	No value	0	0	1
174	C	0	1.453	Infinity	0.911	0.898	0.343
175	F	3.553	0	0	1.079	2.353	0.125
176	I	1.775	0	0	0.729	1.749	0.186
177	F	0	0	No value	0	0	1
178	L	0	0	No value	0	0	1
179	F	0	0	No value	0	0	1
180	L	2.072	0	0	0.857	1.779	0.182
181	E	0	0	No value	0	0	1
182	N	0	0	No value	0	0	1
183	Y	0	0	No value	0	0	1

184	G	3.106	0	0	0.923	2.349	0.125
185	V	0	0	No value	0	0	1
186	R	2.349	0	0	0.767	2.205	0.138
187	K	0	0	No value	0	0	1
188	L	2.025	0	0	1.01	1.345	0.246
189	E	0	0	No value	0	0	1
190	A	0	0	No value	0	0	1
191	A	2.973	1.332	0.448	1.863	0.309	0.578
192	F	0	0	No value	0	0	1
193	G	0	1.616	Infinity	0.85	1.245	0.265
194	I	9.042	1.417	0.157	3.898	2.654	0.103
195	L	1.929	0	0	0.789	1.774	0.183
196	I	0	0	No value	0	0	1
197	G	0	0	No value	0	0	1
198	I	0	0	No value	0	0	1
199	M	0	0	No value	0	0	1
200	A	0	0	No value	0	0	1
201	V	2.955	0	0	1.058	2.107	0.147
202	T	0	1.118	Infinity	0.679	0.957	0.328
203	F	0	0	No value	0	0	1
204	A	1.912	1.515	0.792	1.689	0.026	0.872
205	W	0	1.829	Infinity	1.806	0.025	0.874
206	M	0	0	No value	0	0	1
207	F	0	0	No value	0	0	1
208	A	2.996	0	0	0.868	2.516	0.113
209	D	0	0	No value	0	0	1
210	A	0	0	No value	0	0	1
211	K	0	0	No value	0	0	1
212	P	0	0	No value	0	0	1
213	S	0	0	No value	0	0	1
214	A	1.426	0	0	0.642	1.594	0.207
215	P	3.9	1.138	0.292	2.153	0.986	0.321
216	E	0	0	No value	0	0	1
217	L	3.133	0	0	0.924	2.35	0.125
218	F	0	0	No value	0	0	1
219	L	7.839	0	0	4.251	5.245	0.022
220	G	1.943	0	0	0.788	1.783	0.182
221	I	1.744	0	0	0.727	1.738	0.187
222	L	0	0	No value	0	0	1
223	I	0	0	No value	0	0	1
224	P	1.881	0	0	0.657	2.046	0.153
225	K	0	0	No value	0	0	1
226	L	0	0	No value	0	0	1
227	S	0	0	No value	0	0	1
228	S	0	0	No value	0	0	1
229	K	0	1.168	Infinity	0.809	0.712	0.399
230	T	0	0	No value	0	0	1
231	I	0	0	No value	0	0	1
232	K	0	1.242	Infinity	0.888	0.659	0.417

233	Q	3.606	0	0	0.947	2.53	0.112
234	A	3.324	0	0	0.858	2.62	0.106
235	V	9.822	0	0	2.802	5.58	0.018
236	G	0	0	No value	0	0	1
237	V	0	0	No value	0	0	1
238	V	0	0	No value	0	0	1
239	G	0	0	No value	0	0	1
240	C	0	0	No value	0	0	1
241	I	0	0	No value	0	0	1
242	I	0	0	No value	0	0	1
243	M	0	0	No value	0	0	1
244	P	0	0	No value	0	0	1
245	H	0	0	No value	0	0	1
246	N	0	0	No value	0	0	1
247	V	0	0	No value	0	0	1
248	F	0	0	No value	0	0	1
249	L	0	0	No value	0	0	1
250	H	0	0	No value	0	0	1
251	S	0	0	No value	0	0	1
252	A	0	0	No value	0	0	1
253	L	0	0	No value	0	0	1
254	V	1.917	0	0	0.861	1.569	0.21
255	Q	0	0	No value	0	0	1
256	S	0	0	No value	0	0	1
257	R	0	0	No value	0	0	1
258	E	0	0	No value	0	0	1
259	I	12.387	0	0	4.028	8.678	0.003
260	D	0	0	No value	0	0	1
261	H	0	2.029	Infinity	1.417	1.38	0.24
262	N	0	0	No value	0	0	1
263	K	0	0	No value	0	0	1
264	K	0	0	No value	0	0	1
265	G	0	0	No value	0	0	1
266	Q	1.963	2.898	1.476	2.507	0.097	0.755
267	V	0	0	No value	0	0	1
268	Q	0	0	No value	0	0	1
269	E	0	0	No value	0	0	1
270	A	0	0	No value	0	0	1
271	L	1.451	0	0	0.692	1.465	0.226
272	R	0	0	No value	0	0	1
273	Y	0	0	No value	0	0	1
274	Y	0	0	No value	0	0	1
275	S	0	0	No value	0	0	1
276	I	0	0	No value	0	0	1
277	E	0	0	No value	0	0	1
278	S	0	0	No value	0	0	1
279	T	2.004	0	0	0.698	2.104	0.147
280	A	0	1.182	Infinity	0.83	0.701	0.402

281	A	0	0	No value	0	0	1
282	L	0	0	No value	0	0	1
283	A	0	1.312	Infinity	0.753	1.092	0.296
284	I	0	0	No value	0	0	1
285	S	6.339	0	0	2.916	4.512	0.034
286	F	0	0	No value	0	0	1
287	M	2.025	1.506	0.744	1.605	0.015	0.904
288	I	0	1.345	Infinity	0.737	1.156	0.282
289	N	0	0	No value	0	0	1
290	L	0	0	No value	0	0	1
291	F	0	0	No value	0	0	1
292	V	0	0	No value	0	0	1
293	T	4.524	0	0	1.449	4.343	0.037
294	T	6.503	0	0	2.223	6.293	0.012
295	.	3.263	8.293	2.541	6.069	0.508	0.476
296	F	0	0	No value	0	0	1
297	A	0	0	No value	0	0	1
298	K	0	0	No value	0	0	1
299	G	0	0	No value	0	0	1
300	F	6.037	0	0	1.863	4.385	0.036
301	H	8.263	1.107	0.134	2.543	2.435	0.119
302	G	0	1.377	Infinity	0.781	1.105	0.293
303	T	0	0	No value	0	0	1
304	E	0	0	No value	0	0	1
305	L	1.178	0	0	0.726	0.948	0.33
306	A	0	0	No value	0	0	1
307	N	0	0	No value	0	0	1
308	S	0	0	No value	0	0	1
309	I	0	0	No value	0	0	1
310	G	0	0	No value	0	0	1
311	L	0	0	No value	0	0	1
312	V	0	0	No value	0	0	1
313	N	0	0	No value	0	0	1
314	A	0	0	No value	0	0	1
315	G	0	0	No value	0	0	1
316	Q	0	0	No value	0	0	1
317	Y	0	0	No value	0	0	1
318	L	0	0	No value	0	0	1
319	Q	3.691	0	0	0.928	2.597	0.107
320	D	7.486	0	0	1.843	5.493	0.019
321	K	0	0	No value	0	0	1
322	Y	0	0	No value	0	0	1
323	G	1.951	0	0	0.784	1.776	0.183
324	G	0	0	No value	0	0	1
325	G	1.91	0	0	0.816	1.664	0.197
326	F	0	0	No value	0	0	1
327	F	0	0	No value	0	0	1
328	P	0	0	No value	0	0	1
329	I	0	0	No value	0	0	1

330	L	0	2.266	Infinity	1.071	1.454	0.228
331	Y	0	0	No value	0	0	1
332	I	0	0	No value	0	0	1
333	W	0	0	No value	0	0	1
334	G	3.127	0	0	0.923	2.349	0.125
335	I	4.186	0	0	0.983	2.763	0.096
336	G	1.908	0	0	0.792	1.741	0.187
337	L	0	0	No value	0	0	1
338	L	0	0	No value	0	0	1
339	A	0	0	No value	0	0	1
340	A	0	0	No value	0	0	1
341	G	0	0	No value	0	0	1
342	Q	0	0	No value	0	0	1
343	S	7.48	0	0	1.669	5.858	0.016
344	S	0	0	No value	0	0	1
345	T	0	0	No value	0	0	1
346	I	0	0	No value	0	0	1
347	T	0	0	No value	0	0	1
348	G	1.454	0	0	0.693	1.475	0.224
349	T	3.129	0	0	0.787	2.663	0.103
350	Y	0	0	No value	0	0	1
351	A	0	0	No value	0	0	1
352	G	3.997	0	0	1.643	3.514	0.061
353	Q	0	0	No value	0	0	1
354	F	0	0	No value	0	0	1
355	I	0	0	No value	0	0	1
356	M	0	0	No value	0	0	1
357	G	0	0	No value	0	0	1
358	G	0	0	No value	0	0	1
359	F	0	0	No value	0	0	1
360	L	0	0	No value	0	0	1
361	N	0	0	No value	0	0	1
362	L	0	0	No value	0	0	1
363	G	5.852	1.344	0.23	2.839	1.469	0.225
364	L	0	0	No value	0	0	1
365	K	0	0	No value	0	0	1
366	K	0	0	No value	0	0	1
367	W	0	0	No value	0	0	1
368	L	4.046	0	0	2.387	3.039	0.081
369	R	0	0	No value	0	0	1
370	A	0	0	No value	0	0	1
371	L	2.048	0	0	1.011	1.347	0.246
372	I	2.45	0	0	0.853	2.105	0.147
373	T	0	0	No value	0	0	1
374	R	0	0	No value	0	0	1
375	S	0	0	No value	0	0	1
376	C	0	0	No value	0	0	1
377	A	0	0	No value	0	0	1
378	I	1.758	0	0	0.729	1.747	0.186

379	I	0	1.345	Infinity	0.728	1.166	0.28
380	P	0	0	No value	0	0	1
381	T	0	0	No value	0	0	1
382	I	0	2.192	Infinity	1.978	0.421	0.517
383	I	0	0	No value	0	0	1
384	V	0	0	No value	0	0	1
385	A	1.925	0	0	0.719	1.912	0.167
386	L	0	0	No value	0	0	1
387	V	3.186	0	0	1.053	2.147	0.143
388	F	0	0	No value	0	0	1
389	D	0	0	No value	0	0	1
390	T	1.994	0	0	0.691	2.061	0.151
391	S	7.301	0	0	2.68	5.831	0.016
392	E	0	0	No value	0	0	1
393	D	0	0	No value	0	0	1
394	S	1.925	0	0	0.909	1.447	0.229
395	L	0	0	No value	0	0	1
396	D	0	0	No value	0	0	1
397	V	0	0	No value	0	0	1
398	L	0	0	No value	0	0	1
399	N	0	0	No value	0	0	1
400	E	0	0	No value	0	0	1
401	W	0	0	No value	0	0	1
402	L	0	0	No value	0	0	1
403	N	0	0	No value	0	0	1
404	M	0	1.049	Infinity	0.821	0.481	0.488
405	L	3.19	0	0	0.976	2.432	0.119
406	Q	3.346	0	0	0.925	2.415	0.12
407	S	2.976	0	0	0.977	2.263	0.132
408	I	4.96	0	0	1.081	3.138	0.076
409	Q	0	0	No value	0	0	1
410	I	0	0	No value	0	0	1
411	P	0	0	No value	0	0	1
412	F	7.043	0	0	1.235	3.153	0.076
413	A	0	0	No value	0	0	1
414	L	0	0	No value	0	0	1
415	I	1.756	0	0	0.727	1.745	0.187
416	P	0	0	No value	0	0	1
417	L	0	0	No value	0	0	1
418	L	2.005	0	0	0.801	1.819	0.177
419	C	0	0	No value	0	0	1
420	L	4.313	0	0	2.157	2.704	0.1
421	V	1.812	0	0	0.848	1.523	0.217
422	S	9.857	0	0	3.391	7.201	0.007
423	K	0	0	No value	0	0	1
424	E	0	0	No value	0	0	1
425	Q	0	0	No value	0	0	1
426	.	0	3.661	Infinity	1.629	2.777	0.096
427	M	0	0	No value	0	0	1

428	G	0	0	No value	0	0	1
429	T	0	0	No value	0	0	1
430	F	2.609	0	0	0.884	2.117	0.146
431	T	0	1.191	Infinity	0.776	0.845	0.358
432	V	4.701	1.598	0.34	2.906	0.757	0.384
433	G	1.633	0	0	0.733	1.596	0.206
434	P	0	0	No value	0	0	1
435	I	0	0	No value	0	0	1
436	L	0	0	No value	0	0	1
437	.	0	9.322	Infinity	4.902	4.004	0.045
438	M	0	0	No value	0	0	1
439	V	3.006	1.749	0.582	2.267	0.131	0.717
440	S	0	0	No value	0	0	1
441	W	0	0	No value	0	0	1
442	L	0	1.415	Infinity	0.937	0.794	0.373
443	V	1.906	0	0	0.867	1.569	0.21
444	A	0	0	No value	0	0	1
445	A	0	0	No value	0	0	1
446	L	1.492	0	0	0.869	1.068	0.301
447	V	0	0	No value	0	0	1
448	M	0	0	No value	0	0	1
449	L	1.52	2.286	1.504	1.832	0.076	0.782
450	I	0	0	No value	0	0	1
451	N	0	0	No value	0	0	1
452	G	0	0	No value	0	0	1
453	Y	0	0	No value	0	0	1
454	L	0	0	No value	0	0	1
455	L	5.033	0	0	2.219	2.971	0.085
456	L	3.702	0	0	1.206	2.991	0.084
457	D	0	0	No value	0	0	1
458	F	0	0	No value	0	0	1
459	F	0	0	No value	0	0	1
460	S	0	1.382	Infinity	0.872	0.904	0.342
461	N	0	0	No value	0	0	1
462	E	0	0	No value	0	0	1
463	V	3.97	0	0	1.814	3.142	0.076
464	T	4.204	0	0	1.453	4.217	0.04
465	G	0	0	No value	0	0	1
466	V	0	0	No value	0	0	1
467	.	2.088	6.752	3.234	4.289	1.055	0.304
468	F	0	0	No value	0	0	1
469	T	0	1.125	Infinity	0.614	1.165	0.28
470	T	3.168	0	0	0.789	2.68	0.102
471	V	1.908	0	0	0.867	1.571	0.21
472	V	0	0	No value	0	0	1
473	C	0	0	No value	0	0	1
474	A	0	3.212	Infinity	2.047	1.732	0.188
475	F	0	0	No value	0	0	1
476	T	0	0	No value	0	0	1

477	G	2.909	0	0	0.97	2.226	0.136
478	A	1.924	0	0	0.72	1.913	0.167
479	Y	7.755	0	0	1.312	3.329	0.068
480	V	3.204	0	0	1.057	2.154	0.142
481	T	1.95	2.479	1.271	2.271	0.035	0.852
482	F	0	0	No value	0	0	1
483	I	0	1.125	Infinity	0.863	0.491	0.484
484	I	0	0	No value	0	0	1
485	Y	0	0	No value	0	0	1
486	L	0	0	No value	0	0	1
487	I	4.444	0	0	0.994	2.84	0.092
488	S	0	0	No value	0	0	1
489	R	0	0	No value	0	0	1
490	E	0	2.675	Infinity	1.695	0.312	0.577
491	V	11.922	0	0	3.623	4.557	0.033
492	.	3.85	2.96	0.769	3.412	0.054	0.816
493	I	0	4.109	Infinity	2.808	1.54	0.215
494	S	0	1.402	Infinity	0.77	1.167	0.28
495	T	2.03	1.348	0.664	1.627	0.079	0.779
496	W	0	0	No value	0	0	1
497	Y	2.601	1.625	0.625	1.989	0.104	0.747
498	C	0	0	No value	0	0	1
499	P	3.96	0	0	1.364	4.185	0.041
500	T	6.648	3.08	0.463	4.616	0.633	0.426

Supplementary table S3. Sites under positive and purifying selection in *Populus NRAMP3.2* according to FEL method. Sites subjected to positive and purifying selections were estimated by the FEL method (https://www.datamonkey.org/) (Kosakovsky Pond and Frost 2005). *NRAMP3.2* coding sequences of 13 *Populus* species retrieved as indicated in Materials and Methods were used to generate a phylogenetic tree. A subset of branches encompassing the *NRAMP3.2s* were analyzed separately to estimate the synonymous substitution rate (dS) at a site (α) and the non-synonymous substitution rate (dN) at a site (β), and a maximum-likelihood approach was undertaken to calculate dN/dS rates for each codon site (ϖ). Amino-acids correspond to that of the consensus *Populus* NRAMP3.1 sequence. Dots indicate less than 90% of conservation within all investigated Populus NRAMP3.1 sequences. Sites subjected to positive selection are highlighted in dark ($p < 0.05$) and light green ($p \leq 0.1$). Sites subject to purifying selection are highlighted in dark ($p < 0.05$) and light red ($p < 0.1$). α=β : the rate estimated under the neutral model; LRT: likelihood ratio test.

Site	amino-acid	α	β	ϖ	α=β	LRT	p-value
1	M	0	0	No value	0	0	1
2	P	3.207	2.489	0.776	2.784	0.031	0.86
3	V	0	0	No value	0	0	1
4	-	0	0	No value	0	0	1
5	E	0	0	No value	0	0	1
6	E	0	0	No value	0	0	1
7	N	0	0	No value	0	0	1
8	.	5.82	5.527	0.95	5.656	0.002	0.964
9	Q	0	0	No value	0	0	1
10	P	3.162	2.493	0.788	2.774	0.025	0.875
11	L	0	0	No value	0	0	1
12	L	2.026	0	0	1.417	0.701	0.402
13	Q	0	0	No value	0	0	1
14	E	0	0	No value	0	0	1
15	E	0	0	No value	0	0	1
16	E	0	0	No value	0	0	1
17	E	0	0	No value	0	0	1
18	R	0	0	No value	0	0	1
19	A	0	0	No value	0	0	1
20	Y	0	0	No value	0	0	1
21	D	0	0	No value	0	0	1
22	S	0	0	No value	0	0	1
23	D	3.393	0	0	1.482	1.566	0.211
24	E	0	0	No value	0	0	1
25	K	0	0	No value	0	0	1
26	V	4.189	0	0	2.641	1.786	0.181
27	L	2.005	0	0	1.21	0.984	0.321
28	I	2.724	0	0	1.271	1.46	0.227
29	I	0	0	No value	0	0	1
30	G	1.966	0	0	1.184	0.972	0.324
31	V	3.598	0	0	1.756	1.324	0.25
32	D	0	0	No value	0	0	1
33	S	0	0	No value	0	0	1
34	D	0	0	No value	0	0	1
35	T	9.333	0	0	4.662	5.081	0.024
36	E	0	0	No value	0	0	1

37	S	2.649	0	0	1.186	1.54	0.215
38	G	0	3.103	Infinity	1.507	1.453	0.228
39	G	0	3.139	Infinity	0.976	2.329	0.127
40	S	0	0	No value	0	0	1
41	T	0	0	No value	0	0	1
42	V	0	0	No value	0	0	1
43	L	0	0	No value	0	0	1
44	P	1.903	0	0	1.049	1.151	0.283
45	P	2.32	0	0	1.171	1.333	0.248
46	F	0	0	No value	0	0	1
47	S	0	0	No value	0	0	1
48	W	0	0	No value	0	0	1
49	K	0	0	No value	0	0	1
50	K	4.546	0	0	1.638	1.963	0.161
51	L	0	0	No value	0	0	1
52	W	0	0	No value	0	0	1
53	L	2.183	0	0	1.467	0.728	0.394
54	F	5.723	0	0	2.298	1.919	0.166
55	T	0	0	No value	0	0	1
56	G	0	0	No value	0	0	1
57	P	0	0	No value	0	0	1
58	G	1.949	0	0	1.178	0.961	0.327
59	F	0	0	No value	0	0	1
60	L	0	0	No value	0	0	1
61	M	0	0	No value	0	0	1
62	S	1.704	0	0	1.088	0.856	0.355
63	I	0	0	No value	0	0	1
64	A	3.044	0	0	1.444	1.501	0.221
65	F	0	0	No value	0	0	1
66	L	0	0	No value	0	0	1
67	D	3.407	0	0	1.505	1.58	0.209
68	P	0	0	No value	0	0	1
69	G	6.296	0	0	3.728	3.013	0.083
70	N	0	0	No value	0	0	1
71	L	0	0	No value	0	0	1
72	E	0	0	No value	0	0	1
73	G	0	0	No value	0	0	1
74	D	0	0	No value	0	0	1
75	L	0	0	No value	0	0	1
76	Q	0	0	No value	0	0	1
77	A	0	0	No value	0	0	1
78	G	0	0	No value	0	0	1
79	A	1.979	0	0	1.121	1.071	0.301
80	I	2.45	0	0	1.318	1.215	0.27
81	A	1.942	0	0	1.114	1.063	0.303
82	G	6.48	0	0	3.774	3.104	0.078
83	Y	3.553	0	0	1.793	1.314	0.252
84	S	0	0	No value	0	0	1

85	L	0	0	No value	0	0	1
86	L	0	0	No value	0	0	1
87	W	0	0	No value	0	0	1
88	L	0	0	No value	0	0	1
89	L	1.929	0	0	1.181	0.954	0.329
90	L	0	4.902	Infinity	1.492	2.417	0.12
91	W	0	0	No value	0	0	1
92	A	0	0	No value	0	0	1
93	T	0	0	No value	0	0	1
94	A	0	0	No value	0	0	1
95	M	0	0	No value	0	0	1
96	G	6.514	0	0	3.172	2.834	0.092
97	L	0	0	No value	0	0	1
98	L	0	0	No value	0	0	1
99	V	0	0	No value	0	0	1
100	Q	0	0	No value	0	0	1
101	L	0	0	No value	0	0	1
102	L	1.996	0	0	1.211	0.982	0.322
103	S	0	0	No value	0	0	1
104	A	3.973	0	0	2.267	2.155	0.142
105	R	0	0	No value	0	0	1
106	L	0	0	No value	0	0	1
107	G	1.927	0	0	1.2	0.889	0.346
108	V	0	0	No value	0	0	1
109	A	0	0	No value	0	0	1
110	T	0	0	No value	0	0	1
111	G	3.292	0	0	1.787	1.522	0.217
112	R	2.398	0	0	1.216	1.271	0.26
113	H	0	0	No value	0	0	1
114	L	4.346	0	0	2.942	1.433	0.231
115	A	0	0	No value	0	0	1
116	E	0	0	No value	0	0	1
117	L	1.165	0	0	0.925	0.461	0.497
118	C	0	0	No value	0	0	1
119	R	0	0	No value	0	0	1
120	E	0	0	No value	0	0	1
121	E	0	0	No value	0	0	1
122	Y	0	0	No value	0	0	1
123	P	0	0	No value	0	0	1
124	T	4.515	0	0	2.239	2.464	0.116
125	W	0	0	No value	0	0	1
126	A	3.245	0	0	1.433	1.521	0.217
127	S	0	0	No value	0	0	1
128	M	0	0	No value	0	0	1
129	V	0	3.806	Infinity	1.759	1.546	0.214
130	L	0	0	No value	0	0	1
131	W	0	0	No value	0	0	1
132	I	0	0	No value	0	0	1

133	M	0	0	No value	0	0	1
134	A	0	2.754	Infinity	1.415	1.356	0.244
135	E	0	0	No value	0	0	1
136	L	0	0	No value	0	0	1
137	A	0	0	No value	0	0	1
138	L	0	0	No value	0	0	1
139	I	0	0	No value	0	0	1
140	G	0	0	No value	0	0	1
141	A	0	0	No value	0	0	1
142	D	0	0	No value	0	0	1
143	I	0	0	No value	0	0	1
144	Q	0	0	No value	0	0	1
145	E	3.161	0	0	1.486	1.454	0.228
146	V	0	0	No value	0	0	1
147	I	0	0	No value	0	0	1
148	G	1.908	0	0	1.207	0.889	0.346
149	S	0	0	No value	0	0	1
150	A	0	0	No value	0	0	1
151	I	14.276	0	0	4.079	4.641	0.031
152	A	3.118	0	0	1.415	1.49	0.222
153	.	0	3.19	Infinity	1.692	1.281	0.258
154	K	0	0	No value	0	0	1
155	I	2.45	0	0	1.318	1.215	0.27
156	L	0	0	No value	0	0	1
157	S	0	0	No value	0	0	1
158	N	0	0	No value	0	0	1
159	G	0	0	No value	0	0	1
160	F	0	0	No value	0	0	1
161	.	0	8.927	Infinity	2.985	4.488	0.034
162	P	0	0	No value	0	0	1
163	L	2.038	0	0	1.401	0.692	0.405
164	W	0	0	No value	0	0	1
165	A	0	0	No value	0	0	1
166	G	0	0	No value	0	0	1
167	V	0	0	No value	0	0	1
168	T	0	0	No value	0	0	1
169	I	0	0	No value	0	0	1
170	T	0	0	No value	0	0	1
171	A	3.028	0	0	1.394	1.455	0.228
172	C	4.936	0	0	2.781	2.162	0.141
173	D	0	0	No value	0	0	1
174	.	0	6.937	Infinity	3.048	3.355	0.067
175	F	3.553	0	0	1.642	1.487	0.223
176	I	1.775	0	0	1.086	0.941	0.332
177	F	0	0	No value	0	0	1
178	L	0	0	No value	0	0	1
179	F	0	0	No value	0	0	1
180	L	2.072	0	0	1.419	0.757	0.384

181	E	0	0	No value	0	0	1
182	N	0	0	No value	0	0	1
183	Y	0	0	No value	0	0	1
184	G	3.106	0	0	1.509	1.358	0.244
185	V	0	0	No value	0	0	1
186	R	2.349	0	0	1.242	1.229	0.268
187	K	0	0	No value	0	0	1
188	L	2.025	0	0	1.399	0.695	0.404
189	E	0	0	No value	0	0	1
190	A	0	0	No value	0	0	1
191	V	2.973	0	0	1.65	1.186	0.276
192	F	0	0	No value	0	0	1
193	A	0	0	No value	0	0	1
194	V	9.042	0	0	4.683	3.359	0.067
195	L	1.929	0	0	1.181	0.954	0.329
196	I	0	0	No value	0	0	1
197	G	0	0	No value	0	0	1
198	I	0	2.513	Infinity	1.578	0.935	0.334
199	M	0	0	No value	0	0	1
200	A	0	0	No value	0	0	1
201	V	2.955	0	0	1.642	1.185	0.276
202	T	0	0	No value	0	0	1
203	F	0	0	No value	0	0	1
204	G	1.912	0	0	1.209	0.89	0.345
205	W	0	0	No value	0	0	1
206	M	0	0	No value	0	0	1
207	F	0	0	No value	0	0	1
208	A	2.996	2.985	0.996	2.975	0	1
209	D	0	0	No value	0	0	1
210	A	0	0	No value	0	0	1
211	K	0	0	No value	0	0	1
212	P	0	0	No value	0	0	1
213	S	0	0	No value	0	0	1
214	A	1.426	0	0	0.932	0.841	0.359
215	S	3.9	0	0	2.316	1.879	0.17
216	E	0	0	No value	0	0	1
217	L	3.133	0	0	1.52	1.352	0.245
218	F	0	0	No value	0	0	1
219	L	7.839	0	0	5.407	2.815	0.093
220	G	1.943	0	0	1.18	0.963	0.327
221	I	1.744	0	0	1.079	0.935	0.334
222	L	0	0	No value	0	0	1
223	I	0	0	No value	0	0	1
224	P	1.881	0	0	1.037	1.134	0.287
225	K	0	0	No value	0	0	1
226	L	0	0	No value	0	0	1
227	S	0	0	No value	0	0	1
228	S	0	0	No value	0	0	1

229	R	0	0	No value	0	0	1
230	T	0	0	No value	0	0	1
231	I	0	0	No value	0	0	1
232	Q	0	0	No value	0	0	1
233	Q	3.606	0	0	1.603	1.484	0.223
234	A	3.324	0	0	1.455	1.554	0.213
235	V	9.822	0	0	4.376	3.413	0.065
236	G	0	0	No value	0	0	1
237	V	0	0	No value	0	0	1
238	V	0	0	No value	0	0	1
239	G	0	0	No value	0	0	1
240	C	0	0	No value	0	0	1
241	I	0	0	No value	0	0	1
242	I	0	0	No value	0	0	1
243	M	0	0	No value	0	0	1
244	P	0	0	No value	0	0	1
245	H	0	0	No value	0	0	1
246	N	0	0	No value	0	0	1
247	V	0	0	No value	0	0	1
248	F	0	0	No value	0	0	1
249	L	0	0	No value	0	0	1
250	H	0	0	No value	0	0	1
251	S	0	0	No value	0	0	1
252	A	0	0	No value	0	0	1
253	L	0	0	No value	0	0	1
254	V	1.917	0	0	1.237	0.834	0.361
255	Q	0	0	No value	0	0	1
256	S	0	0	No value	0	0	1
257	R	0	0	No value	0	0	1
258	E	0	0	No value	0	0	1
259	I	12.387	0	0	5.669	5.406	0.02
260	D	0	0	No value	0	0	1
261	H	0	0	No value	0	0	1
262	N	0	2.549	Infinity	1.747	0.791	0.374
263	K	0	0	No value	0	0	1
264	K	0	0	No value	0	0	1
265	.	0	9.06	Infinity	2.311	4.965	0.026
266	R	1.963	0	0	1.191	0.943	0.332
267	V	0	0	No value	0	0	1
268	Q	0	0	No value	0	0	1
269	E	0	0	No value	0	0	1
270	A	0	0	No value	0	0	1
271	L	1.451	0	0	0.983	0.759	0.384
272	R	0	0	No value	0	0	1
273	Y	0	0	No value	0	0	1
274	Y	0	0	No value	0	0	1
275	S	0	0	No value	0	0	1
276	I	0	0	No value	0	0	1

277	E	0	0	No value	0	0	1
278	S	0	0	No value	0	0	1
279	T	2.004	0	0	1.102	1.174	0.278
280	T	0	0	No value	0	0	1
281	A	0	0	No value	0	0	1
282	L	0	0	No value	0	0	1
283	V	0	0	No value	0	0	1
284	I	0	0	No value	0	0	1
285	S	6.339	0	0	3.928	2.601	0.107
286	F	0	0	No value	0	0	1
287	V	2.025	4.376	2.161	3.372	0.154	0.695
288	I	0	0	No value	0	0	1
289	N	0	0	No value	0	0	1
290	L	0	0	No value	0	0	1
291	F	0	0	No value	0	0	1
292	V	0	0	No value	0	0	1
293	T	4.524	0	0	2.278	2.497	0.114
294	T	6.503	0	0	3.37	3.629	0.057
295	V	3.263	0	0	1.378	1.142	0.285
296	F	0	0	No value	0	0	1
297	A	0	0	No value	0	0	1
298	K	0	0	No value	0	0	1
299	G	0	0	No value	0	0	1
300	F	6.037	0	0	2.912	2.56	0.11
301	Y	8.263	0	0	3.57	2.706	0.1
302	G	0	0	No value	0	0	1
303	T	0	0	No value	0	0	1
304	E	0	0	No value	0	0	1
305	L	1.178	0	0	0.925	0.462	0.497
306	A	0	0	No value	0	0	1
307	N	0	0	No value	0	0	1
308	S	0	0	No value	0	0	1
309	I	0	0	No value	0	0	1
310	G	0	0	No value	0	0	1
311	L	0	0	No value	0	0	1
312	V	0	0	No value	0	0	1
313	N	0	0	No value	0	0	1
314	A	0	0	No value	0	0	1
315	G	0	0	No value	0	0	1
316	Q	0	0	No value	0	0	1
317	Y	0	0	No value	0	0	1
318	L	0	0	No value	0	0	1
319	Q	3.691	0	0	1.578	1.543	0.214
320	D	7.486	0	0	2.899	3.57	0.059
321	K	0	0	No value	0	0	1
322	Y	0	0	No value	0	0	1
323	G	1.951	0	0	1.174	0.962	0.327
324	G	0	0	No value	0	0	1

325	G	1.91	0	0	1.201	0.888	0.346
326	F	0	0	No value	0	0	1
327	F	0	0	No value	0	0	1
328	P	0	0	No value	0	0	1
329	I	0	0	No value	0	0	1
330	L	0	0	No value	0	0	1
331	Y	0	0	No value	0	0	1
332	I	0	0	No value	0	0	1
333	W	0	0	No value	0	0	1
334	G	3.127	0	0	1.508	1.357	0.244
335	I	4.186	0	0	1.685	1.671	0.196
336	G	1.908	0	0	1.207	0.889	0.346
337	L	0	0	No value	0	0	1
338	L	0	0	No value	0	0	1
339	A	0	0	No value	0	0	1
340	A	0	0	No value	0	0	1
341	G	0	0	No value	0	0	1
342	Q	0	0	No value	0	0	1
343	S	7.48	0	0	2.72	3.815	0.051
344	S	0	0	No value	0	0	1
345	T	0	0	No value	0	0	1
346	I	0	0	No value	0	0	1
347	T	0	0	No value	0	0	1
348	G	1.454	0	0	0.984	0.768	0.381
349	T	3.129	0	0	1.345	1.582	0.208
350	Y	0	0	No value	0	0	1
351	A	0	0	No value	0	0	1
352	G	3.997	0	0	2.466	1.837	0.175
353	Q	0	0	No value	0	0	1
354	F	0	0	No value	0	0	1
355	I	0	0	No value	0	0	1
356	M	0	0	No value	0	0	1
357	G	0	0	No value	0	0	1
358	G	0	0	No value	0	0	1
359	F	0	0	No value	0	0	1
360	L	0	0	No value	0	0	1
361	N	0	0	No value	0	0	1
362	L	0	0	No value	0	0	1
363	R	5.852	0	0	2.731	2.948	0.086
364	L	0	0	No value	0	0	1
365	K	0	0	No value	0	0	1
366	K	0	0	No value	0	0	1
367	W	0	0	No value	0	0	1
368	L	4.046	0	0	3.066	1.509	0.219
369	R	0	0	No value	0	0	1
370	A	0	0	No value	0	0	1
371	L	2.048	0	0	1.403	0.694	0.405
372	I	2.45	0	0	1.318	1.215	0.27

373	T	0	0	No value	0	0	1
374	R	0	0	No value	0	0	1
375	S	0	0	No value	0	0	1
376	C	0	0	No value	0	0	1
377	A	0	0	No value	0	0	1
378	I	1.758	0	0	1.085	0.94	0.332
379	I	0	0	No value	0	0	1
380	P	0	0	No value	0	0	1
381	T	0	0	No value	0	0	1
382	M	0	0	No value	0	0	1
383	I	0	0	No value	0	0	1
384	V	0	0	No value	0	0	1
385	A	1.925	0	0	1.104	1.05	0.306
386	L	0	0	No value	0	0	1
387	V	3.186	0	0	1.656	1.225	0.268
388	F	0	0	No value	0	0	1
389	D	0	0	No value	0	0	1
390	.	1.994	5.36	2.688	3.569	0.63	0.427
391	S	7.301	0	0	4.004	3.278	0.07
392	E	0	0	No value	0	0	1
393	D	0	0	No value	0	0	1
394	S	1.925	0	0	1.287	0.752	0.386
395	L	0	0	No value	0	0	1
396	D	0	0	No value	0	0	1
397	V	0	0	No value	0	0	1
398	L	0	0	No value	0	0	1
399	N	0	0	No value	0	0	1
400	E	0	0	No value	0	0	1
401	W	0	0	No value	0	0	1
402	L	0	0	No value	0	0	1
403	N	0	0	No value	0	0	1
404	V	0	0	No value	0	0	1
405	L	3.19	0	0	1.832	1.133	0.287
406	Q	3.346	0	0	1.544	1.4	0.237
407	S	2.976	0	0	1.742	1.085	0.298
408	I	4.96	0	0	1.675	2.215	0.137
409	Q	0	0	No value	0	0	1
410	I	0	0	No value	0	0	1
411	P	0	0	No value	0	0	1
412	F	7.043	0	0	2.205	1.987	0.159
413	A	0	0	No value	0	0	1
414	L	0	0	No value	0	0	1
415	I	1.756	0	0	1.083	0.937	0.333
416	P	0	0	No value	0	0	1
417	L	0	0	No value	0	0	1
418	L	2.005	0	0	1.21	0.984	0.321
419	C	0	3.379	Infinity	1.428	1.728	0.189
420	L	4.313	0	0	2.94	1.438	0.23

421	V	1.812	0	0	1.205	0.803	0.37
422	S	9.857	0	0	4.875	4.219	0.04
423	K	0	0	No value	0	0	1
424	E	0	0	No value	0	0	1
425	Q	0	0	No value	0	0	1
426	I	0	0	No value	0	0	1
427	M	0	0	No value	0	0	1
428	G	0	0	No value	0	0	1
429	T	0	0	No value	0	0	1
430	F	2.609	0	0	1.399	1.192	0.275
431	K	0	0	No value	0	0	1
432	I	4.701	0	0	2.555	2.309	0.129
433	G	1.633	3.132	1.918	2.168	0.202	0.653
434	P	0	0	No value	0	0	1
435	I	0	3.074	Infinity	1.681	1.235	0.266
436	L	0	0	No value	0	0	1
437	.	0	6.063	Infinity	3.151	2.719	0.099
438	M	0	0	No value	0	0	1
439	V	3.006	0	0	1.66	1.196	0.274
440	.	0	6.576	Infinity	3.174	2.974	0.085
441	W	0	0	No value	0	0	1
442	L	0	0	No value	0	0	1
443	V	1.906	0	0	1.242	0.832	0.362
444	A	0	0	No value	0	0	1
445	A	0	0	No value	0	0	1
446	L	1.492	0	0	1.113	0.57	0.45
447	V	0	0	No value	0	0	1
448	.	0	4.623	Infinity	4.382	0.225	0.635
449	V	1.52	0	0	1.062	0.691	0.406
450	I	0	0	No value	0	0	1
451	N	0	0	No value	0	0	1
452	G	0	0	No value	0	0	1
453	Y	0	0	No value	0	0	1
454	L	0	0	No value	0	0	1
455	L	5.033	0	0	3.127	1.601	0.206
456	L	3.702	0	0	1.98	1.704	0.192
457	D	0	0	No value	0	0	1
458	F	0	0	No value	0	0	1
459	F	0	0	No value	0	0	1
460	.	0	11.774	Infinity	2.531	2.954	0.086
461	N	0	0	No value	0	0	1
462	E	0	0	No value	0	0	1
463	V	3.97	0	0	2.536	1.707	0.191
464	.	4.204	5.359	1.275	4.754	0.053	0.818
465	G	0	0	No value	0	0	1
466	V	0	0	No value	0	0	1
467	A	2.088	0	0	1.157	1.116	0.291
468	F	0	0	No value	0	0	1

469	T	0	0	No value	0	0	1
470	T	3.168	0	0	1.351	1.595	0.207
471	V	1.908	0	0	1.241	0.832	0.362
472	V	0	3.933	Infinity	1.247	2.296	0.13
473	C	0	0	No value	0	0	1
474	G	0	0	No value	0	0	1
475	F	0	0	No value	0	0	1
476	T	0	0	No value	0	0	1
477	G	2.909	0	0	1.503	1.318	0.251
478	A	1.924	0	0	1.105	1.051	0.305
479	Y	7.755	0	0	2.387	2.118	0.146
480	.	3.204	7.665	2.392	5.456	0.467	0.494
481	A	1.95	2.887	1.481	2.343	0.069	0.792
482	F	0	0	No value	0	0	1
483	I	0	0	No value	0	0	1
484	I	0	0	No value	0	0	1
485	Y	0	0	No value	0	0	1
486	L	0	0	No value	0	0	1
487	I	4.444	0	0	1.682	1.772	0.183
488	S	0	0	No value	0	0	1
489	R	0	0	No value	0	0	1
490	G	0	0	No value	0	0	1
491	F	11.922	3.8	0.319	6.783	0.833	0.361
492	T	3.85	2.567	0.667	3.282	0.107	0.744
493	C	0	0	No value	0	0	1
494	F	0	0	No value	0	0	1
495	S	2.03	3.202	1.577	2.5	0.1	0.752
496	.	0	7.81	Infinity	5.699	1.252	0.263
497	C	2.601	0	0	1.409	1.167	0.28
498	C	0	0	No value	0	0	1
499	.	3.96	5.692	1.437	4.719	0.122	0.727
500	S	6.648	0	0	3.803	3.051	0.081
501	K	0	0	No value	0	0	1
502	Q	0	0	No value	0	0	1
503	I	0	0	No value	0	0	1
504	E	0	0	No value	0	0	1
505	V	0	0	No value	0	0	1
506	E	0	0	No value	0	0	1

Supplementary table S4. Primers used for the cloning of *P. trichocarpa NRAMP3*.

Primer names	Primer sequences
PtNramp3aLgtw	5'-TACAAAAAAGCAGGCTTCATGCCTTCACCAGAAGAAGAC-3'
PtNramp3aRgtwnostp	5'-CAAGAAAGCTGGGTCGGTGGGACAGTACCAAGTGG-3'
PtNramp3aRgtwstp	5'-CAAGAAAGCTGGGTCCTGTTTAGGTGGGACAGTACCA-3'
PtNramp3bLlonggtw	5'-GGGGACAAGTTTGTACAAAAAAGCAGGCTTCATGCCTGTAGAAGAAAAC-3'
PtNramp3bRlonggtwnostp	5'-GGGGACCACTTTGTACAAGAAAGCTGGGTCTGTAAAGCCCCTAGAATG-3'

PtNramp3bRlonggtwstp	5'-GGGGACCACTTTGTACAAGAAAGCTGGGTCTTATGTAAAGCCCCTAG-3'
U5	5'-GGGGACAAGTTTGTACAAAAAAGCAGGCTTC-3'
U3	5'-GGGGACCACTTTGTACAAGAAAGCTGGGTC-3'

Supplementary table S5. Composition of media used for poplar transformation and *in vitro* propagation.

Composition	MS30	M1	M2	M3	MS1/2
Macro-elements (10X)[1]	100 mL/L	100 mL/L	100 mL/L	100 mL/L	50 mL/L
Micro-elements (1000X)[2]	1 mL/L	1 mL/L	1 mL/L	1 mL/L	1 mL/L
Ethylenediaminetetra-acetic acid ferric monosodium salt(Fe)	40 mg/L	40 mg/L	40 mg/L	40 mg/L	40 mg/L
Myo-inositol	100 mg/L	100 mg/L	100 mg/L	100 mg/L	100 mg/L
M.E.S		250 mg/L	250 mg/L	250 mg/L	
Vitamins (100X)[3]	10 mL/L	10 mL/L	10 mL/L	10 mL/L	10 mL/L
L-Glutamine	200 mg/L	200 mg/L	200 mg/L	200 mg/L	200 mg/L
Sucrose	30 g/L	30 g/L	30 g/L	30 g/L	20 g/L
pH	5.9 – 6	5.8	5.8	5.8	5.9 - 6
Agar		7 g/L	7 g/L	7 g/L	7 g/L
N_6-(2-Isopentenyl) adenine (2ip)		5 µM	5 µM		
α-Naphthaleneacetic Acid (NAA)		10 µM	10 µM		
Ticarpen			500 mg/L	500 mg/L	
Cefotaxime			250 mg/L	250 mg/L	
Thidiazuron (TDZ)				0.1 µM	

[1]**Macro-elements (10X):** 16.5 g NH_4NO_3, 19 g KNO_3, 4.4 g $CaCl_2.2H_2O$, 3.7 g $MgSO_4.7H_2O$, 1.7 g KH_2PO_4 dissolved in 1000 ml H_2O.

[2]**Micro-elements (1000X):** 620 mg H_3BO_3, 1690 mg $MnSO_4$.H2O, 1060 mg $ZnSO_4.7H_2O$, 83 mg KI, 25 mg $Na_2MoO_4.2H_2O$, 2.5 mg $CuSO_4.5H_2O$, 2.5 mg $CoCl_2.6H_2O$ dissolved in 100 ml H_2O.

[3]**Vitamins (100X):** 50 mg Nicotinic acid, 50 mg Pyridoxine hydrochloride, 50 mg Thiamine hydrochloride, 50 mg Calcium pantothenate, 50 mg L-cysteine chlorohydrate, 5 ml of Biotine (50 mg/ 50 ml NaOH) dissolved in 500 ml H_2O.

Supplementary table S6. Primers used for the RT-qPCR.

Genes	Forward primers	Reverse primers
PotriNRAMP3.1	TGACATTTGCGTGGATGTTT	ATTGTGGTCGATCTCCCTTG
PotriNRAMP3.2	CGGCCCCATTCTTAAGATGG	TGTAAAGCCCCTAGAAATGAGAT
PtEF1	CCACACCTGTCACATTGCTG	ACCAGCATCACCGTTCTTCAG
PtPP2A	TTCCTGATGTGCGACTGAAC	CTCCAATGCCTATCCTCTGC
PtUBQ	CCATATCCAAGGTATTGCTCTCC	CGTCTCATACTTGTTCCTGTGG
AtACTIN	GGTAACATTGTGCTCAGTGGTGG	GGTAACATTGTGCTCAGTGGTGG

Supplementary Data

Supplementary data S1. NRAMP3 coding sequences identified in *Populus* and *Salix* genomes

>P. trichocarpa_NRAMP3.1_CDS
ATGCCTTCACCAGAAGAAGACCCACAACCTTTATTAAAAGACCAAGAAGAAACAGCTTATGATT
CTGACGGGAAAGTCCTTTCATTTGGGATTGATTATGACACAGAAAGCGGTGGCTCAACGGTGGT
GCCATCATTTTCATGGAGAAAATTATGGTTGTTCACTGGTCCTGGGTTTTTAATGTGCATTGCTTT
TTTGGACCCTGGCAATTTGGAAGGGGATCTTCAGGCTGGTGCAATTGCAGGGTATTCTTTGCTTT
GGCTTCTCTTATGGGCTACTGCTATGGGTTTGTTGGTGCAGTTGCTGTCAGCAAGGCTTGGAGTG
GCTACAGGAAGGCATTTGGCTGAGCTATGTAGAGAAGAGTATCCAACTTGGGCTCGAATGATTT
TGTGGATTATGGCTGAGTTGGCTTTGATTGGTGCTGATATACAAGAAGTTATTGGGAGTGCTATT
GCTATTCAGATTTTGAGTAATGGGGTTTTGCCTTTGTGGGCTGGTGTTATTATTACTGCTTCCGAT
TGCTTTATCTTCCTATTTCTTGAGAACTACGGTGTGAGGAAATTGGAGGCTGCTTTTGGGATTCTC
ATTGGAATAATGGCAGTGACATTTGCGTGGATGTTTGCTGATGCAAAACCCAGTGCCCCCGAACT
TTTTCTGGGCATCTTAATTCCAAAACTTAGCTCCAAAACAATAAAACAGGCTGTTGGAGTTGTGG
GTTGCATTATCATGCCTCACAATGTGTTCTTGCATTCTGCTCTTGTACAGTCAAGGGAGATCGAC
CACAATAAGAAAGGCCAGGTTCAAGAAGCTCTCAGATACTACTCCATAGAGTCAACTGCTGCCC
TTGCAATATCATTCATGATCAATTTGTTTGTGACGACCATTTTTGCTAAAGGTTTCCACGGGACA
GAACTGGCCAATAGTATTGGCCTTGTAAATGCAGGGCAATATCTTCAAGATAAATACGGGGGTG
GATTTTTCCCAATTTTATACATCTGGGGTATTGGGTTATTAGCAGCTGGCCAAAGTAGCACCATT
ACTGGCACTTATGCAGGGCAGTTTATCATGGGAGGTTTCCTGAACTTGGGGTTAAAGAAATGGCT
GAGGGCATTGATTACTCGAAGCTGTGCTATCATCCCAACTATAATTGTTGCACTTGTTTTTGATAC
TTCTGAAGACTCACTAGATGTTCTGAATGAATGGCTAAATATGCTTCAGTCTATTCAGATTCCTTT
TGCACTCATCCCTCTTCTTTGCTTGGTCTCTAAGGAGCAAATCATGGGCACTTTCACAGTTGGCCC
CATTCTTAAGATGGTTTCTTGGCTTGTAGCTGCCTTGGTGATGCTAATCAATGGTTACCTTTTGCT
TGACTTTTTCTCCAATGAAGTAACTGGAGTAGTGTTTACCACTGTGGTATGCGCTTTTACAGGAG
CATATGTTACGTTTATAATTTATCTCATTTCTAGGGAAGTTACCATTTCCACTTGGTACTGTCCCA
CC

>P. trichocarpa_NRAMP3.2_CDS
ATGCCTGTAGAAGAAAACTACCAACCTTTATTGCAAGAAGAAGAAGAAAGAGCTTATGATTCTG
ATGAGAAAGTGCTCATAATTGGGGTTGATTCTGACACGGAAAGCGGTGGCTCAACGGTGTTGCC
ACCGTTTTCATGGAAAAAGTTATGGTTGTTTACTGGTCCTGGGTTTTTAATGTCCATTGCGTTTTT
GGATCCTGGGAATTTGGAAGGGGATCTTCAGGCTGGTGCAATCGCAGGCTACTCTTTGCTTTGGC
TTCTTTTATGGGCTACTGCTATGGGGTTGTTGGTGCAGTTGCTTTCAGCGAGGCTTGGAGTGGCT
ACAGGGAGGCATTTAGCTGAGCTGTGTAGAGAAGAGTATCCAACCTGGGCTTCAATGGTTTTGT
GGATTATGGCTGAGTTGGCTTTGATTGGTGCTGATATACAAGAGGTTATTGGAAGTGCTATTGCT
ATTAAGATCTTGAGTAATGGGTTTGTGCCTTTGTGGGCTGGTGTTACTATTACTGCTTGTGATTGC
TTCATCTTCCTATTTCTAGAGAACTACGGTGTGAGAAAATTGGAGGCTGTATTTGCGGTCCTTATT
GGAATAATGGCAGTTACATTTGGATGGATGTTTGCAGATGCAAAACCCAGTGCCTCCGAACTTTT
TCTGGGTATCTTAATTCCAAAACTTAGCTCCAGAACAATACAACAGGCTGTTGGAGTTGTGGGTT
GCATTATCATGCCTCACAATGTGTTCTTGCATTCTGCTCTTGTACAGTCAAGGGAGATCGACCAC
AATAAGAAAGACCGGGTTCAAGAAGCTCTCAGATACTACTCCATAGAGTCAACCACTGCCCTTG
TAATATCGTTCGTAATCAATTTGTTTGTGACGACTGTTTTTGCTAAAGGTTTCTACGGGACAGAA
CTAGCCAATAGTATTGGCCTTGTAAATGCAGGGCAATATCTTCAAGACAAATACGGGGGTGGAT
TTTTCCCAATTTTATACATCTGGGGTATTGGATTATTAGCAGCTGGCCAAAGCAGCACCATTACT
GGCACTTATGCAGGACAGTTTATCATGGGAGGTTTCCTGAACTTGAGGTTAAAGAAATGGCTTA
GGGCATTGATCACTCGAAGCTGTGCTATCATCCCAACTATGATTGTTGCACTTGTTTTTGATACCT
CCGAAGACTCACTAGATGTTCTGAATGAATGGCTAAATGTGCTGCAGTCAATACAGATTCCTTTT

GCACTCATCCCTCTTCTCTGCTTAGTATCCAAGGAGCAAATCATGGGCACTTTCAAAATCGGCCC
CATTCTTAAGATGGTAGCTTGGCTTGTGGCTGCCCTGGTGATGGTAATCAATGGTTACCTTTTGCT
CGACTTTTTCTTCAATGAAGTGACCGGAGTAGCGTTTACCACTGTAGTATGCGGTTTTACAGGTG
CATATGTTGCGTTTATAATTTATCTCATTTCTAGGGGCTTTACATGTTTCTCCCGGTGCTGTCCAT
CTAAACAGATAGAAGTAGAG

>P. alba_NRAMP3.1_CDS
ATGCCTTTACCAGAAGAAGACCCGCAACCTTTATTAAAAGACCAAGAAGAAACAGCTTATGATT
CTGACGGGAAAGTCCTTTCATTTGGCATTGATTATGACACGGAAAGCGGTGGCTCAACGGCGGT
GTCATCATTTTCATGGAGAAAATTATGGTTGTTCACTGGTCCTGGGTTTTTAATGTGCATTGCTTT
TTTGGACCCTGGCAATTTGGAAGGGGATCTTCAGGCTGGTGCAATTGCAGGGTATTCTTTGCTTT
GGCTTCTCTTATGGGCTACTGCTATGGGTTTGTTGGTGCAGTTGCTGTCAGCAAGGCTTGGAGTG
GCTACAGGAAGGCATTTGGCTGAGCTATGTAGAGAAGAGTATCCAACTTGGGCTCGAATGATTT
TGTGGATTATGGCTGAGTTGGCTTTGATTGGTGCTGATATACAAGAAGTTATTGGGAGTGCTATT
GCTATTCAGATTTTGAGTAATGGGGTTTTGCCTTTGTGGGCTGGTGTTATTATTACTGCTTTCGAT
TGCTTTATCTTCCTATTTCTTGAGAACTACGGTGTGAGGAAATTGGAGGCTGCTTTTGGGATCCTC
ATTGGAATAATGGCAGTGACATTTGCGTGGATGTTTGCTGATGCAAAACCCAGTGCCCCTGAACT
TTTTCTAGGCATCTTAATTCCAAAACTTAGCTCCAAAACAATAAAACAGGCTGTTGGAGTTGTGG
GTTGCATTATCATGCCTCACAATGTGTTCTTGCATTCTGCTCTTGTACAGTCAAGGGAGATTGACC
ACAATAAGAAAGGCCAGGTTCAAGAAGCTCTCAGATACTACTCCATAGAGTCAACTGCTGCCCT
TGCAATATCATTCATGATCAATTTGTTTGTGACGACCATTTTTGCTAAAGGTTTCCACGGGACAG
AACTGGCCAATAGTATTGGCCTTGTAAATGCAGGGCAATATCTTCAAGATAAATACGGGGGTGG
ATTTTTCCCAATTTTATACATCTGGGGTATTGGGTTATTAGCAGCTGGCCAAAGTAGCACCATTA
CTGGCACTTATGCAGGGCAGTTTATCATGGGAGGTTTCCTGAACTTGGGATTAAAGAAATGGCTG
AGGGCATTGATTACTCGAAGCTGTGCTATCATCCCAACTATAATTGTTGCACTTGTTTTTGATACT
TCTGAAGACTCACTAGATGTTCTGAATGAATGGCTAAATATGCTTCAGTCTATTCAGATTCCTTTT
GCACTCATCCCTCTTCTTTGCTTGGTCTCCAAGGAGCAAATCATGGGCACTTTCACAGTTGGCCC
CATTCTTAAGATGTTTTCTTGGCTTGTAGCTGCCTTGGTGATGCTAATCAATGGTTACCTTTTGCT
TGACTTTTTCTCCAATGAAGTAACTGGAGTAGTGTTTACCACTGTGGTATGCTCTTTTACAGGAG
CATATGTTACGTTTATAATTTATCTCATTTCTAGGGAAGTATCCATTTCCACTTGGTACTGTCCCA
CC

>P. alba_NRAMP3.2_CDS
ATGCCTGTAGAAGAAAACCAACAACCTTTATTGCAAGAAGAAGAAGAAAGAGCTTATGATTCTG
ATGAGAAAGTGCTCATAATTGGGGTTGATTCTGACACGGAAAGCGGTGGCTCAACGGTGTTGCC
ACCGTTTTCATGGAAAAAGTTATGGTTGTTTACTGGTCCTGGGTTTTTAATGTCCATTGCGTTTTT
GGATCCTGGGAATTTGGAAGGGGATCTTCAGGCTGGTGCTATCGCAGGCTACTCTTTGCTTTGGC
TTCTTTTATGGGCTACTGCTATGGGTTTGTTGGTGCAGTTGCTTTCAGCGAGGCTTGGAGTGGCTA
CAGGGAGGCATTTAGCTGAGCTGTGTAGAGAAGAGTATCCAACCTGGGCGTCAATGGTTTTGTG
GATTATGGCTGAGTTGGCTTTGATTGGTGCTGATATACAAGAGGTTATTGGAAGTGCTATTGCTC
TTAAGATCTTGAGTAATGGGTTTTTGCCTCTGTGGGCTGGTGTTACTATTACTGCTTGTGATTGCT
TCATCTTCCTATTTCTAGAGAACTACGGTGTGAGAAAATTGGAGGCTGTATTTGCGGTCCTTATT
GGAATAATGGCAGTTACATTTGGATGGATGTTTGCAGATGCAAAACCCAGTGCCTCCGAACTTTT
TCTGGGTATCTTAATTCCCAAACTTAGCTCCAGAACAATACAACAGGCTGTTGGAGTTGTGGGTT
GCATTATCATGCCTCACAATGTGTTCTTGCATTCTGCTCTTGTGCAGTCAAGGGAGATCGACCAC
AATAAGAAAGGCCGGGTTCAAGAAGCTCTCAGATACTACTCCATAGAGTCAACCACTGCCCTTG
TAATATCATTCGTAATCAATTTGTTTGTGACGACTGTTTTTGCTAAAGGTTTCTATGGGACAGAAC
TGGCCAATAGTATTGGCCTTGTAAATGCAGGGCAATATCTTCAAGACAAATACGGGGGTGGATT
TTTCCCAATTTTATACATCTGGGGTATTGGATTATTAGCAGCTGGCCAAAGCAGCACCATTACTG
GCACTTATGCAGGACAGTTTATCATGGGAGGTTTCCTGAACTTGAGGTTAAAGAAATGGTTAAG

GGCATTGATCACTCGAAGCTGTGCTATCATCCCAACTATGATTGTTGCACTTGTTTTTGATACCTC
TGAAGACTCACTAGATGTTCTGAATGAATGGCTAAATGTGCTGCAGTCAATACAGATTCCTTTTG
CACTCATCCCTCTTCTCTGCTTGGTATCCAAGGAGCAAATCATGGGCACTTTCAAAATCGGTCCC
ATTCTTAAGGTATCTTGGCTTGTGGCTGCCCTGGTGATAGTAATCAATGGTTACCTTTTGCTCGAC
TTTTTCGTCAATGAAGTGGCCGGAGTAGCGTTTACCACTGTACTATGCGGTTTTACAGGTGCATA
TGTAGCGTTTATAATTTATCTCATTTCTAGGGGCTTTACATGTTTCTTCTGGTGCTGTCAATCTAA
ACAGATAGAAGTAGAG

>P. cathayana_NRAMP3.1_CDS
ATGCCTTTACCAGAAGAAGACCCACAACCTTTATTAAAAGACCAAGAAGAAACAGCTTATGATT
CTGACGGGAAAGTCCTTTCATTTGGGATTGATTATGACACCGAAAGCGGTGGCTCAACGGTGGT
GCCATCATTTTCATGGAGAAAATTATGGTTGTTCACTGGTCCTGGGTTTTTAATGTGCATTGCTTT
TTTGGACCCTGGCAATTTGGAAGGGGATCTTCAGGCTGGTGCAATTGCAGGGTATTCTTTGCTTT
GGCTTCTCTTATGGGCTACTGCTATGGGTTTGTTGGTGCAGTTGCTGTCAGCAAGGCTTGGAGTG
GCTACAGGAAGGCATTTGGCTGAGCTATGTAGAGAAGAGTATCCAACTTGGGCTCGAATGATTT
TGTGGATTATGGCTGAGTTGGCTTTGATTGGTGCTGATATACAAGAAGTTATTGGGAGTGCTATT
GCTATTCAGATTTTGAGTAATGGGGTTTTGCCTTTGTGGGCTGGTGTTATTATTACTGCTTCCGAT
TGCTTTATCTTCCTATTTCTTGAGAACTACGGTGTGAGGAAATTGGAGGCTGCTTTTGGGATTCTC
ATTGGAATAATGGCAGTGACATTTGCGTGGATGTTTGCTGATGCAAAACCCAGTGCCCCCGAACT
TTTTCTGGGCATCTTAATTCCAAAACTTAGCTCCAAAACAATAAAACAGGCTGTTGGAGTTGTGG
GTTGCATTATCATGCCTCACAATGTGTTCTTGCATTCTGCTCTTGTACAGTCAAGGGAGATCGAC
CACAATAAGAAAGGCCAGGTTCAAGAAGCTCTCAGATACTACTCCATAGAGTCAACTGCTGCCC
TTGCAATATCATTCATGATCAATTTGTTTGTGACGACCGTTTTTGCTAAAGGTTTCCACGGGACA
GAACTGGCCAATAGTATTGGCCTTGTAAATGCAGGGCAATATCTTCAAGATAAATACGGGGGTG
GATTTTTCCCAATTTTATACATCTGGGGTATTGGGTTATTAGCAGCTGGCCAAAGTAGCACCATT
ACTGGCACTTATGCAGGGCAGTTTATCATGGGAGGTTTCCTGAACTTGGGGTTAAAGAAATGGCT
GAGGGCATTGATTACTCGAAGCTGTGCTATCATCCCAACTATAATTGTTGCACTTGTTTTTGATAC
TTCTGAAGACTCACTAGATGTTCTGAATGAATGGCTAAATATGCTTCAGTCTATTCAGATTCCTTT
TGCACTCATCCCTCTTCTTTGCTTGGTCTCTAAGGAGCAAATCATGGGCACTTTCACAGTTGGCCC
CATTCTTCAGATGGTTTCTTGGCTTGTAGCTGCCTTGGTGATGCTAATCAATGGTTACCTTTTGCT
TGACTTTTTCTCCAATGAAGTGACTGGAGTAGTGTTTACCACTGTGGTATGCGCTTTTACAGGAG
CATATGTTACGTTTATAATTTATCTCATTTCTAGGGAAGTTACCATTTCCACTTGGTACTGTCCTA
CC

>P. cathayana_NRAMP3.2_CDS
ATGCCTGTAGAAGAAAACCACCAACCTTTATTGCAAGAAGAAGAAGAAAGAGCTTATGATTCTG
ATGAGAAAGTGCTCATAATTGGGGTCGATTCTGACACGGAAAGCGGTGGCTCAACGGTGTTGCC
ACCGTTTTCATGGAAAAAGTTATGGTTGTTTACTGGTCCTGGGTTTTTAATGTCCATTGCGTTTTT
GGATCCTGGGAATTTGGAAGGGGATCTTCAGGCTGGTGCAATCGCAGGCTACTCTTTGCTTTGGC
TTCTTTTATGGGCTACTGCTATGGGGTTGTTGGTGCAGTTGCTTTCAGCGAGGCTTGGAGTGGCT
ACAGGGAGACATTTAGCTGAGCTGTGTAGAGAAGAGTATCCAACCTGGGCTTCAATGGTTTTGT
GGATTATGGCTGAGTTGGCTTTGATTGGTGCTGATATACAAGAGGTTATTGGAAGTGCTATTGCT
ATTAAGATCTTGAGTAATGGGTTTGTGCCTTTGTGGGCTGGTGTTACTATTACTGCTTGTGATTGC
TTCATCTTCCTATTTCTAGAGAACTACGGTGTGAGAAAATTGGAGGCTGTATTTGCGGTCCTTATT
GGAATAATGGCAGTTACATTTGGATGGATGTTTGCAGATGCAAAACCCAGTGCCTCCGAACTTTT
TCTGGGTATCTTAATTCCAAAACTTAGCTCCAGAACAATACAACAGGCTGTTGGAGTTGTGGGTT
GCATTATCATGCCTCACAATGTGTTCTTGCATTCTGCTCTTGTACAGTCAAGGGAGATCGACCAC
AATAAGAAAGACCGGGTTCAAGAAGCTCTCAGATACTACTCCATAGAGTCAACCACTGCCCTTG
TAATATCGTTCGTAATCAATTTGTTTGTGACGACTGTTTTTGCTAAAGGTTTCTACGGGACAGAA
CTGGCCAATAGTATTGGCCTTGTAAATGCAGGGCAATATCTTCAAGACAAATACGGGGGTGGAT

TTTTCCCAATTTTATACATCTGGGGTATTGGATTATTAGCAGCTGGCCAAAGCAGCACCATTACT
GGCACTTATGCAGGACAGTTTATCATGGGAGGTTTCCTGAACTTGAGGTTAAAGAAATGGCTAA
GGGCATTGATCACTCGAAGCTGTGCTATCATCCCAACTATGATTGTTGCACTTGTTTTTGATACCT
CCGAAGACTCACTAGATGTTCTGAATGAATGGCTAAATGTGCTGCAGTCAATACAGATTCCTTTT
GCACTCATCCCTCTTCTCTGCTTAGTATCCAAGGAGCAAATCATGGGCACTTTCAAAATCGGCCC
CATTCTTAAGATGGTAGCTTGGCTTGTGGCTGCCCTGGTGATGGTAATCAATGGTTACCTTTTGCT
CGACTTTTTCTTCAATGAAGTGACCGGAGTAGCATTTACCACAGTAGTATGCGGTTTTACAGGTG
CATATGCTGCGTTTATAATTTATCTCATTTCTAGGGGCTTTACATGTTTCTCCCGGTGCTGTCCAT
CTAAACAGATAGAAGTAGAG

>P. simonii_NRAMP3.1_CDS
ATGCCTTTACCAGAAGAAGACCCACGACCTTTATTAAAAGACCAAGAAGAAACAGCTTATGATT
CTGACGGGAAAGTCCTTTCATTTGGGATTGATTATGACACCGAAAGCGGTGGCTCAACGGTGGT
GCCATCATTTTCATGGAGAAAATTATGGTTGTTCACTGGTCCTGGGTTTTTAATGTGCATTGCTTT
TTTGGACCCTGGCAATTTGGAAGGGGATCTTCAGGCTGGTGCAATTGCAGGGTATTCTTTGCTTT
GGCTTCTCTTATGGGCTACTGCTATGGGTTTGTTGGTGCAGTTGCTGTCAGCAAGGCTTGGAGTG
GCTACAGGAAGGCATTTGGCTGAGCTATGTAGAGAAGAGTATCCAACTTGGGCTCGAATGATTT
TGTGGATTATGGCTGAGTTGGCTTTGATTGGTGCTGATATACAAGAAGTTATTGGGAGTGCTATT
GCTATTCAGATTTTGAGTAATGGGGTTTTGCCTTTGTGGGCTGGTGTTATTATTACTGCTTCCGAT
TGCTTTATCTTCCTATTTCTTGAGAACTACGGTGTGAGGAAATTGGAGGCTGCTTTTGGGATTCTC
ATTGGAATAATGGCAGTGACATTTGCGTGGATGTTTGCTGATGCAAAACCCAGTGCCCCCGAACT
TTTTCTGGGCATCTTAATTCCAAAACTTAGCTCCAAAACAATAAAACAGGCTGTTGGAGTTGTGG
GTTGCATTATCATGCCTCACAATGTGTTCTTGCATTCTGCTCTTGTACAGTCAAGGGAGATTGACC
ACAATAAGAAAGGCCAGGTTCAAGAAGCTCTCAGATACTACTCCATAGAGTCAACTGCTGCCCT
TGCAATATCATTCATGATCAATTTGTTTGTGACGACTGTTTTTGCTAAAGGTTTCCACGGGACAG
AACTGGCCAATAGTATTGGCCTTGTAAATGCAGGGCAATATCTTCAAGATAAATACGGGGGTGG
ATTTTTCCCAATTTTATACATCTGGGGTATTGGGTTATTAGCAGCTGGCCAAAGTAGCACCATTA
CTGGCACTTATGCAGGGCAGTTTATCATGGGAGGTTTCCTGAACTTGGGGTTAAAGAAATGGCTG
AGGGCATTGATTACTCGAAGCTGTGCTATCATCCCAACTACAATTGTTGCACTTGTTTTTGATACT
TCTGAAGACTCACTAGATGTTCTGAATGAATGGCTAAATATGCTTCAGTCTATTCAGATTCCTTTT
GCACTCATCCCTCTTCTTTGCTTGGTCTCTAAGGAGCAAATCATGGGCACTTTCACAGTTGGCCCC
ATTCTTCAGATGGTTTCTTGGCTTGTAGCTGCCTTGGTGATGCTAATCAATGGTTACCTTTTGCTT
GACTTTTTCTCCAATGAAGTAACTGGAGTAGTGTTTACCACTGTGGTATGCGCTTTTACAGGAGC
ATATGTTACGTTTATAATTTATCTCATTTCTAGGGAAGTTACCATTTCCACTTGGTACTGTCCCAC
C

>P. simonii_NRAMP3.2_CDS
ATGCCTGTAGAAGAAAACCACCAACCTTTATTGCAAGAAGAAGAAGAAAGAGCTTATGATTCTG
ATGAGAAAGTGCTCATAATTGGGGTTGATTCTGACACGGAAAGCGGTGGCTCAACGGTGTTGCC
ACCGTTTTCATGGAAAAAGTTATGGTTGTTTACTGGTCCTGGGTTTTTAATGTCCATTGCGTTTTT
GGATCCTGGGAATTTGGAAGGGGATCTTCAGGCTGGTGCAATCGCAGGCTACTCTTTGCTTTGGC
TTCTTTTATGGGCTACTGCTATGGGGTTGTTGGTGCAGTTGCTTTCAGCGAGGCTTGGAGTGGCT
ACAGGGAGGCATTTAGCTGAGCTGTGTAGAGAAGAGTATCCAACCTGGGCTTCAATGGTTTTGT
GGATTATGACTGAGTTGGCTTTGATTGGTGCTGATATACAAGAGGTTATTGGAAGTGCTATTGCT
ATTAAGATCTTGAGTAATGGGTTTGTGCCTTTGTGGGCTGGTGTTACTATTACTGCTTGTGATTTC
ATCTTCCTATTTCTAGAGAACTACGGTGTGAGAAAATTGGAGGCTGTATTTGCGGTCCTTATTGG
AATAATGGCAGTTACATTTGGATGGATGTTTGCAGATGCAAAACCCAGTGCTTCCGAACTTTTTC
TGGGTATCTTAATTCCAAAACTTAGCTCCAGAACAATACAACAGGCTGTTGGAGTTGTGGGTTGC
ATTATCATGCCTCACAATGTGTTCTTGCATTCTGCTCTTGTACAGTCAAGGGAGATCGACCACAA
TAAGAAAGACCGGGTTCAAGAAGCTCTCAGATACTACTCCATAGAGTCAACCACTGCCCTTGTA

ATATCGTTCGTAATCAATTTGTTTGTGACGACTGTTTTTGCTAAAGGTTTCTACGGGACAGAACT
GGCCAATAGTATTGGCCTTGTAAATGCAGGGCAATATCTTCAAGACAAATACGGGGGTGGATTT
TTCCCAATTTTATACATCTGGGGTATTGGATTATTAGCAGCTGGCCAAAGCAGCACCATTACTGG
CACTTATGCAGGACAGTTTATCATGGGAGGTTTCCTGAACTTGAGGTTAAAGAAATGGCTAAGG
GCATTGATCACTCGAAGCTGTGCTATCATCCCAACTATGATTGTTGCACTTGTTTTTGATACCTCC
GAAGACTCACTAGATGTTCTGAATGAATGGCTAAATGTGCTGCAGTCAATACAGATTCCTTTTGC
ACTCATCCCTCTTCTCTGCTTAGTATCCAAGGAGCAAATCATGGGCACTTTCAAAATCGGCCCCA
TTCTTAAGATGGTATCTTGGCTTGTGGCTGCCCTGGTGATGGTAATCAATGGTTACCTTTTACTCG
ACTTTTTCTTCAATGAAGTGACCGGAGTAGCGTTTACCACTGTAGTATGCGGTTTTACAGGTGCA
TATGTTGCGTTTATAATTTATCTCATTTCTAGGGGCTTTACATGTTTCTCCCGGTGCTGTCCATCTA
AACAGATAGAAGTAGAG

>P. lasiocarpa _NRAMP3.1_CDS
ATGCCTTTACCAGAAGAAGACCCAAAACCTTTATTAAAAGACCAAGAAGAAACAGCTTATGATT
CTGACGGGAAAGTCCTTTCATTTGGGATTGATTATGGCACCGAAAGCGGTGGCTCAACGGTGGT
GCCATCATTTTCATGGAGAAAATTATGGTTGTTCACTGGTCCTGGGTTTTTAATGTGCATTGCTTT
TTTGGACCCTGGCAATTTGGAAGGGGATCTTCAGGCTGGTGCAATTGCTGGGTATTCTTTGCTTT
GGCTTCTCTTATGGGCTACTGCTATGGGTTTGTTGGTGCAGTTGCTGTCAGCAAGGCTTGGAGTG
GCTACAGGAAGGCATTTGGCTGAGCTATGTAGAGAAGAGTATCCAACTTGGGCTCGAATGATTT
TGTGGATTATGGCTGAGTTGGCTTTGATTGGTGCTGATATACAAGAAGTTATTGGGAGTGCTATT
GCTATTCAGATTTTGAGTAATGGGGTTTTGCCTTTGTGGGCTGGTGTTATTATTACTGCTTCCGAT
TGCTTTATCTTCCTATTTCTTGAGAACTACGGTGTGAGGAAATTGGAGGCTGCTTTTGGGATTCTC
ATTGGAATAATGGCAGTGACATTTGCGTGGATGTTTGCTGATGCAAAACCCAGTGCCCCCGAACT
TTTTCTGGGCATCTTAATTCCAAAACTTAGCTCCAAAACAATAAAACAGGCTGTTGGAGTTGTGG
GTTGCATTATCATGCCTCACAATGTGTTCTTGCATTCTGCTCTTGTACAGTCAAGGGAGATTGACC
ACAATAAGAAAGGCCAGGTTCAAGAAGCTCTCAGATACTACTCCATAGAGTCAACTGCTGCCCT
TGCAATATCATTCATGATCAATTTGTTTGTGACGACCGTTTTTGCTAAAGGTTTCCATGGGACAG
AACTGGCCAATAGTATTGGCCTTGTAAATGCAGGGCAATATCTTCAAGATAAATACGGAGGTGG
ATTTTTCCCAATTTTTTACATCTGGGGTATTGGGTTATTAGCAGCTGGCCAAAGTAGCACCATTAC
TGGCACTTATGCAGGGCAGTTTATCATGGGAGGTTTCCTGAACTTGGGGTTAAAGAAATGGCTG
AGGGCATTGATTACTCGAAGCTGTGCTATCATCCCAACTATAATTGTTGCACTTGTATTTGATACT
TCTGAAGACTCACTAGATGTTCTGAATGAATGGCTAAATATGCTTCAGTCTATTCAGATTCCTTTT
GCACTCATCCCTCTTCTTTGCTTGGTCTCCAAGGAGCAAATCATGGGCACTTTCACAGTTGGCCC
CATTCTTCAGATGGTTTCTTGGCTTGTAGCTGCCTTGGTGATGCTAATCAATGGTTACCTTTTGCT
TGACTTTTTCTCCAATGAAGTAACTGGAGTAGTGTTTACCACTGTGGTATGCGCTTTTACAGGAG
CATATGTTACGTTTATAATTTATCTCATTTCTAGGGAAGTTACCATTTCCACTTGGTACTGTCCCA
CC

>P. lasiocarpa _NRAMP3.2_CDS
ATGCCTGTAGAAGAAAACCACCAACCTTTATTGCAAGAAGAAGAAGAAAGAGCTTATGATTCTG
ATGAGAAAGTGCTCATAATTGGGGTTGATTCTGACACGGAAAGCGGTGGCTCAACGGTGTTGCC
ACCGTTTTCATGGAAAAAGTTATGGTTGTTTACTGGTCCTGGGTTTTTAATGTCCATTGCGTTTTT
GGATCCTGGGAATTTGGAAGGGGATCTTCAGGCTGGTGCAATCGCAGGCTACTCTTTGCTTTGGC
TTCTTTTATGGGCTACTGCTATGGGGTTGTTGGTGCAGTTGCTTTCAGCGAGGCTTGGAGTGGCT
ACAGGGAGGCATTTAGCTGAGCTGTGTAGAGAAGAGTATCCAACCTGGGCTTCAATGGTTTTGT
GGATTATGGCTGAGTTGGCTTTGATTGGTGCTGATATACAAGAGGTTATTGGAAGTGCTATTGCT
ATTAAGATCTTGAGTAATGGGTTTGTGCCTTTGTGGGCTGGTGTTACTATTACTGCTTGTGATTGG
TTCATCTTCCTATTTCTAGAGAACTACGGCGTGAGAAAATTGGAGGCTGTATTTGCGGTCCTTAT
TGGAATAATGGCAGTTACATTTGGATGGATGTTTGCAGATGCAAAACCCAGTGCCTCCGAACTTT
TTCTGGGTATCTTAATTCCAAAACTTAGCTCCAGAACAATACAACAGGCTGTTGGAGTTGTGGGT

TGCATTATCATGCCTCACAATGTGTTCTTGCATTCTGCTCTTGTACAGTCAAGGGAGATCGACCA
CAATAAGAAAGACCGGGTTCAAGAAGCTCTCAGATACTACTCCATAGAGTCAACCACTGCCCTT
GTAATATCATTCGTAATCAATTTGTTTGTGACGACTGTTTTTGCTAAAGGTTTCTACGGGACAGA
ACTGGCCAATAGTATTGGCCTTGTAAATGCAGGGCAATATCTTCAAGACAAATACGGGGGTGGA
TTTTTCCCAATTTTATACATCTGGGGTATTGGATTATTAGCAGCTGGCCAAAGCAGCACCATTAC
TGGCACTTATGCAGGACAGTTTATCATGGGAGGTTTCCTGAACTTGAGGTTAAAGAAATGGCTA
AGGGCATTGATCACTCGAAGCTGTGCTATCATCCCAACTATGATTGTTGCACTTGTTTTTGATACC
TCCGAAGACTCACTAGATGTTCTGAATGAATGGCTAAATGTGCTGCAGTCAATACAGATTCCTTT
TGCACTCATCCCTCTTCTCTGCTTAGTATCCAAGGAGCAAATCATGGGCACTTTCAAAATCGACC
CCATTCTTAAGATGGTATCTTGGCTTGTGGCTGCCCTGGTGATGGTAATCAATGGTTACCTTTTGC
TCGACTTTTTCTTCAATGAAGTGACCGGAGTAGCGTTTACCACTGTAGTATGCGGTTTTACAGGT
GCATATGTTGCGTTTATAATTTATCTCATTTCTAGGGGCTTTACATGTTTCTCCCGGTGCTGTCCA
TCTAAACAGATAGAAGTAGAG

>P. maximowiczii _NRAMP3.1_CDS
ATGCCTTTACCAGAAGAAGACCCACAACCTTTATTAAAAGACCAAGAAGAAACAGCTTATGATT
CTGACGGGAAAGTCCTTTCATTTGGGATTGATTATGACACCGAAAGCGGTGGCTCAACGGTGGT
GCCATCATTTTCATGGAGAAAATTATGGTTGTTCACTGGTCCTGGGTTTTTAATGTGCATTGCTTT
TTTGGACCCTGGCAATTTGGAAGGGGATCTTCAGGCTGGTGCAATTGCAGGGTATTCTTTGCTTT
GGCTTCTCTTATGGGCTACTGCTATGGGTTTGTTGGTGCAGTTGCTGTCAGCAAGGCTTGGAGTG
GCTACAGGAAGGCATTTGGCTGAGCTATGTAGAGAAGAGTATCCAACTTGGGCTCGAATGATTT
TGTGGATTATGGCTGAGTTGGCTTTGATTGGTGCTGATATACAAGAAGTTATTGGGAGTGCTATT
GCTATTCAGATTTTGAGTAATGGGGTTTTGCCTTTGTGGGCTGGTGTTATTATTACTGCTTCCGAT
TGCTTTATCTTCCTATTTCTTGAGAACTACGGTGTGAGGAAATTGGAGGCTGCTTTTGGGATTCTC
ATTGGAATAATGGCAGTGACATTTGCGTGGATGTTTGCTGATGCAAAACCCAGTGCCCCCGAACT
TTTTCTAGGCATCTTAATTCCAAAACTTAGCTCCAAAACAATAAAACAGGCTGTTGGAGTTGTGG
GTTGCATTATCATGCCTCACAATGTGTTCTTGCATTCTGCTCTTGTACAGTCAAGGGAGATTGACC
ACAATAAGAAAGGCCAAGTTCAAGAAGCTCTCAGATACTACTCCATAGAGTCAACTGCTGCCCT
TGCAATATCATTCATGATCAATTTGTTTGTGACGACTGTTTTTGCTAAAGGTTTCCACGGGACAG
AACTGGCCAATAGTATTGGCCTTGTAAATGCAGGGCAATATCTTCAAGATAAATACGGGGGTGG
ATTTTTCCCAATTTTATACATCTGGGGTATTGGGTTATTAGCAGCTGGCCAAAGTAGCACCATTA
CTGGCACTTATGCAGGGCAGTTTATCATGGGAGGTTTCCTGAACTTGGGGTTAAAGAAATGGCTG
AGGGCATTGATTACTCGAAGCTGTGCTATCATCCCAACTATAATTGTTGCACTTGTTTTTGATACT
TCTGAAGACTCACTAGATGTTCTGAATGAATGGCTAAATATGCTTCAGTCTATTCAGATTCCTTTT
GCACTCATCCCTCTTCTTTGCTTGGTCTCTAAGGAGCAAATCATGGGCACTTTCACAGTTGGCCCC
ATTCTTAAGATGGTTTCTTGGCTTGTAGCTGCCTTGGTGATGCTAATCAATGGTTACCTTTTGCTT
GACTTTTTCTCCAATGAAGTGACTGGAGTAGTGTTTACCACTGTGGTATGCGCTTTTACAGGAGC
ATATGTTACGTTTATAATTTATCTCATTTCTAGGGAAGTTACCATTTCCACTTGGTACTGTCCTAC
C

>P. maximowiczii _NRAMP3.2_CDS
ATGCCTGTAGAAGAAAACCACCAACCTTTATTGCAAGAAGAAGAAGAAAGAGCTTATGATTCTG
ATGAGAAAGTGCTCATAATTGGGGTTGATTCTGACACGGAAAGCGGTGGCTCAACGGTGTTGCC
ACCGTTTTCATGGAAAAAGTTATGGTTGTTTACTGGTCCTGGGTTTTTAATGTCCATTGCGTTTTT
GGATCCTGGGAATTTGGAAGGGGATCTTCAGGCTGGTGCAATCGCAGGCTACTCTTTGCTTTGGC
TTCTTTTATGGGCTACTGCTATGGGGTTGTTGGTGCAGTTGCTTTCAGCGAGGCTTGGAGTGGCT
ACAGGGAGACATTTAGCTGAGCTGTGTAGAGAAGAGTATCCAACCTGGGCTTCAATGGTTTTGT
GGATTATGGCTGAGTTGGCTTTGATTGGTGCTGATATACAAGAGGTTATTGGAAGTGCTATTGCT
ATTAAGATCTTGAGTAATGGGTTTGTGCCTTTGTGGGCTGGTGTTACTATTACTGCTTGTGATTGC
TTCATCTTCCTATTTCTAGAGAACTACGGTGTGAGAAAATTGGAGGCTGTATTTGCGGTCCTTATT

GGAATAATGGCAGTTACATTTGGATGGATGTTTGCAGATGCAAAACCCAGTGCCTCCGAACTTTT
TCTGGGTATCTTAATTCCAAAACTTAGCTCCAGAACAATACAACAGGCTGTTGGAGTTGTGGGTT
GCATTATCATGCCTCACAATGTGTTCTTGCATTCTGCTCTTGTACAGTCAAGGGAGATCGACCAC
AATAAGAAAGACCGGGTTCAAGAAGCTCTCAGATACTACTCCATAGAGTCAACCACTGCCCTTG
TAATATCGTTCGTAATCAATTTGTTTGTGACGACTGTTTTTGCTAAAGGTTTCTACGGGACAGAA
CTGGCCAATAGTATTGGCCTTGTAAATGCAGGGCAATATCTTCAAGACAAATACGGGGGTGGAT
TTTTCCCAATTTTATACATCTGGGGTATTGGATTATTAGCAGCTGGCCAAAGCAGCACCATTACT
GGCACTTATGCAGGACAGTTTATCATGGGAGGTTTCCTGAACTTGAGGTTAAAGAAATGGCTAA
GGGCATTGATCACTCGAAGCTGTGCTATCATCCCAACTATGATTGTTGCACTTGTTTTTGATACCT
CCGAAGACTCACTAGATGTTCTGAATGAATGGCTAAATGTGCTGCAGTCAATACAGATTCCTTTT
GCACTCATCCCTCTTCTCTGCTTAGTATCCAAGGAGCAAATCATGGGCACTTTCAAAATCGGCCC
CATTCTTAAGATGGTAGCTTGGCTTGTGGCTGCCCTGGTGATGGTAATCAATGGTTACCTTTTGCT
CGACTTTTTCTTCAATGAAGTGACTGGAGTAGCGTTTACCACTGTAGTATGCGGTTTTACAGGTG
CATATGCTGCGTTTATAATTTATCTCATTTCTAGGGGCTTTACATGTTTCTCCCGGTGTTGTCCAT
CTAAACAGATAGAAGTAGAG

>P. euphratica_NRAMP3.1_CDS
ATGCCTTTACCAGAAGAAGACCCCCAACCTTTATTAGAAGACCAAGAAGAAACAGCTTATGATT
CTAACGGGAAAGTCCTTTCGTTTGGGATTGATTATGACACCGAAAGTGGTGGCTCAACGGTGGT
GCCGTCATTTTCATGGAGAAAATTATGGTTGTTCACTGGTCCTGGGTTTTTAATGTGCATTGCTTT
TTTGGACCCTGGCAATTTGGAAGGGGATCTTCAGGCTGGTGCAATTGCAGGATATTCTTTGCTTT
GGCTTCTCTTATGGGCTACTGCTATGGGTTTGTTGGTGCAGTTGCTGTCAGCGAGGCTTGGAGTG
GCTACAGGAAGGCATTTGGCTGAGCTATGGAGAGAAGAGTAnCCAACTTGGGCTCGAATGATTT
TGTGGATTATGGCTGAGTTGGCTTTGATTGGTGCTGATATACAAGAAGTTATTGGGAGTGCTATT
GCTATTCAGATTTTGAGTAATGGGGTTTTGCCTTTGTGGGCTGGTGTTATTATTACTGCCTCCGAT
AGCTTTATCTTCCTATTTCTTGAGAACTACGGTGTGAGGAAATTGGAGGCTGCTTTTGGGATTCTC
ATTGGAATAATGGCAGTGACATTTGCGTGCATGTTTGCTGATGCAAAACCCAGTGCCCCCGAACT
TTTTCTAGGCATCTTAATTCCAAAACTTAGCTCCAAAACAATAAAACAGGCAGTTGGAGTTGTGG
GTTGCATTATCATGCCTCACAATGTGTTCTTGCATTCTGCTCTTGTACAGTCAAGGGAGATTGACC
ACAATAAGAAAGGCCGGGTTCAAGAAGCTCTCAGATACTACTCCATAGAGTCAACTGCTGCCCT
TGCAATATCATTCATGAACAATTTGTTTGTGACAACCGTGTTTGCTAAAGGTTTCCACAGGACAG
AACTGGCCAATAGTATTGGCCTTGTAAATGCAGGGCAATATCTTCAGGATAAATACGGGGGTGG
ATTTTTCCCAATTTTATACATCTGGGGTATTGGGTTATTAGCAGCTGGCCAAAGTAGCACCATTA
CTGGCACTTATGCAGGGCAGTTTATCATGGGAGGTTTCCTGAACTTGGGGTTAAAGAAATGGCTG
AGGGCATTGATTACTCGAAGCTGTGCTATCGTCCCAACTATAATTGTTGCACTTGTTTTTGATACT
TCTGAAGACTCACTAGATGTTCTGAATGAATGGCTAAATATGCTTCAGTCTATTCAGATTCCTTTT
GCACTCATCCCTCTTCTTTGCTTGGTCTCTAAGGAGCAAATCATGGGCACTTTCACAGTTGGCCCC
ATTCTTAAGATGGTTTCTTGGTTTGTAGCTGCCTTGGTGATGCTAATCAATGGTTACCTTTTGCTT
GACTTTTTCTCCAATGAAGTAACTGGAGTAGTGTTTATCACTGTGGTATGCGCTTTTACAGGAGC
ATATGTTACGTTTACAATTTATCTCATTTCTAGGGAAGTTACCATTTCCACTTGGTACTGTCCCTC
C

>P. euphratica_NRAMP3.2_CDS
ATGCCTGTAGAAGAAAACCAACAACTTTTATTGCAAGAAGAAGAAGAAGATAGAGCTTATGATT
CTGATGAGAAAGTGCTCATAATTGGGGTTGATTCTGACACTGAAAGCAGTGGCTCAACGGTGTT
GCCACCGTTTTCATGGAAAAAGTTATGGTTATTTACTGGTCCTGGGTTTTTAATGTCCATTGCGTT
TTTGGATCCTGGAAATTTGGAAGGGGATCTTCAGGCTGGTGCAATCGCAGGCTACTCTTTGCTTT
GGCTTCTTTTATGGGCTACTGCTATGGGGTTGTTGGTGCAGTTGCTTTCAGCGAGGCTTGGAGTG
GCTACAGGAAGGCATTTAGCTGAGCTGTGTAGAGAAGAGTATCCAACCTGGGCTTCAATGGTTT
TGTGGATTATGGCTGAGTTGGCTTTGATTGGTGCTGATATACAAGAGGTTATTGGAAGTGCTATT

GCTATTAAGATCTTGAGTAATGGGTTTTTCCTTTGTGGGCTGGTGTTACTATTACTGCTTGTGAT
TGCTTCATCTTCCTATTTCTAGAGAACTACGGTGTGAGAAAATTGGAGGCTGTATTTGCGGTACT
TATTGGAATAATGGCAGTTACATTTGGATGGATGTTTGGAGATGCAAAACCCAGTGCCTCCGAA
CTTTTTCTGGGTATCTTAATTCCAAAACTTAGCTCCAGAACAATACAACAAGCTGTTGGAGTTGT
GGGTTGCATTATCATGCCTCACAATGTGTTCTTGCATTCTGCTCTTGTACAGTCAAGGGAGATCG
ACCACAGTAAGAAAGACCGGGTTCAAGAAGCTCTCAGATACTACTCCATAGAGTCAACCACTGC
CCTTGTAATATCGTTCCTAATCAATTTGTTTGTGACAACTGTTTTTGCTAAAGGTTTCTACGGGAC
AGAACTGGCCAATAGTATTGGCCTTGTAAATGCAGGGCAATATCTTCAAGACAAATACGGGGGT
GGATTTTTCCCAATTTTATACATCTGGGGTATTGGATTATTAGCAGCTGGCCAAAGCAGCACCAT
TACTGGCACTTATGCAGGACAGTTTATCATGGGAGGTTTCCTGAACTTGAGGTTAAAGAAATGGC
TAAGGGCATTGATCACTCGAAGCTGTGCTATCATCCCAACTATGATTGTTGCACTTGTTTTTGATA
CCTCAGAAGACTCACTAGATGTTCTGAATGAATGGCTAAATGTGCTTCAGTCAATACAGATTCCT
TTTGCACTCATCCCTCTTCTCTGCTTAGTATCCAAGGAGCAAATCATGGGCACTTTCAAAATCGG
CCCCATTCTTCAGATGGTATCTTGGCTTGTGGCTGCCCTGGTGATAGTAATCAATGGTTACCTTTT
GCTTGACTTTTTCGTAAATGAAGTGACCGGAGTAGCGTTTACCACTGTAGTATGCGGTTTTACAG
GTGCATACGTTGTGTTTATAATTTATCTCATTTCTAGGGGCGTTACATGTTTCTCCTGGTGCTGTC
CATCAAAACAGATAGAAGTAGAG

>P. ussuriensis_NRAMP3.1_CDS
ATGCCTTTACCAGAAGAAGACCCACAACCTTTATTAAAAGACCAAGAAGAAACAGCTTATGATT
CTGACGGGAAAGTCCTTTCATTTGGGATTGATTATGACACCGAAAGCGGTGGCTCAACGGTGGT
GCCATCATTTTCATGGAGAAAATTATGGTTGTTCACTGGTCCTGGGTTTTTAATGTGCATTGCTTT
TTTGGACCCTGGCAATTTGGAAGGGGATCTTCAGGCTGGTGCAATTGCAGGGTATTCTTTGCTTT
GGCTTCTCTTATGGGCTACTGCTATGGGTTTGTTGGTGCAGTTGCTGTCAGCAAGGCTTGGAGTG
GCTACAGGAAGGCATTTGGCTGAGCTATGTAGAGAAGAGTATCCAACTTGGGCTCGAATGATTT
TGTGGATTATGGCTGAGTTGGCTTTGATTGGTGCTGATATACAAGAAGTTATTGGGAGTGCTATT
GCTATTCAGATTTTGAGTAATGGGGTTTTGCCTTTGTGGGCTGGTGTTATTATTACTGCTTCCGAT
TGCTTTATCTTCCTATTTCTTGAGAACTACGGTGTGAGGAAATTGGAGGCTGCTTTTGGGATTCTC
ATTGGAATAATGGCAGTGACATTTGCGTGGATGTTTGCTGATGCAAAACCCAGTGCCCCCGAACT
TTTTCTGGGCATCTTAATTCCAAAACTTAGCTCCAAAACAATAAAACAGGCTGTTGGAGTTGTGG
GTTGCATTATCATGCCTCACAATGTGTTCTTGCATTCTGCTCTTGTACAGTCAAGGGAGATTGACC
ACAATAAGAAAGGCCAGGTTCAAGAAGCTCTCAGATACTACTCCATAGAGTCAACTGCTGCCCT
TGCAATATCATTCATGATCAATTTGTTTGTGACGACTGTTTTTGCTAAAGGTTTCCACGGGACAG
AACTGGCCAATAGTATTGGCCTTGTAAATGCAGGGCAATATCTTCAAGATAAATACGGGGGTGG
ATTTTTCCCAATTTTATACATCTGGGGTATTGGGTTATTAGCAGCTGGCCAAAGTAGCACCATTA
CTGGCACTTATGCAGGGCAGTTTATCATGGGAGGTTTCCTGAACTTGGGGTTAAAGAAATGGCTG
AGGGCATTGATTACTCGAAGCTGTGCTATCATCCCAACTATAATTGTTGCACTTGTTTTTGATACT
TCTGAAGACTCACTAGATGTTCTGAATGAATGGCTAAATATGCTTCAGTCTATTCAGATTCCTTTT
GCACTCATCCCTCTTCTTTGCTTGGTCTCTAAGGAGCAAATCATGGGCACTTTCACAGTTGGCCCC
ATTCTTCAGATGGTTTCTTGGCTTGTAGCTGCCTTGGTGATGCTAATCAATGGTTACCTTTTGCTT
GACTTTTTCTCCAATGAAGTGACTGGAGTAGTGTTTACCACTGTGGTATGCGCTTTTACAGGAGC
ATATGTTACGTTTATAATTTATCTCATTTCTAGGGAAGTTACCATTTCCACTTGGTACTGTCCTAC
C

>P. ussuriensis_NRAMP3.2_CDS
ATGCCTGTAGAAGAAAACCACCAACCATTATTGCAAGAAGAAGAAGAAAGAGCTTATGATTCTG
ATGAGAAAGTGCTCATAATTGGGGTTGATTCTGACACGGAAAGCGGTGGCTCAACGGTGTTGCC
ACCGTTTTCATGGAAAAAGTTATGGTTGTTTACTGGTCCTGGGTTTTTAATGTCCATTGCGTTTTT
GGATCCTGGGAATTTGGAAGGGGATCTTCAGGCTGGTGCAATCGCAGGCTACTCTTTGCTTTGGC
TTCTTTTATGGGCTACTGCTATGGGGTTGTTGGTGCAGTTGCTTTCAGCGAGGCTTGGAGTGGCT

ACAGGGAGACATTTAGCTGAGCTGTGTAGAGAAGAGTATCCAACCTGGGCTTCAATGGTTTTGT
GGATTATGGCTGAGTTGGCTTTGATTGGTGCTGATATACAAGAGGTTATTGGAAGTGCTATTGCT
ATTAAGATCTTGAGTAATGGGTTTGTGCCTTTGTGGGCTGGTGTTACTATTACTGCTTGTGATTGC
TTCATCTTCCTATTTCTAGAGAACTACGGTGTGAGAAAATTGGAGGCTGTATTTGCGGTCCTTATT
GGAATAATGGCAGTTACATTTGGATGGATGTTTGCAGATGCAAAACCCAGTGCCTCCGAACTATT
TCTGGGTATCTTAATTCCAAAACTTAGCTCCAGAACAATACAACAGGCTGTTGGAGTTGTGGGTT
GCATTATCATGCCTCACAATGTGTTCTTGCATTCTGCTCTTGTACAGTCAAGGGAGATCGACCAC
AATAAGAAAGACCGGGTTCAAGAAGCTCTCAGATACTACTCCATAGAGTCAACCACTGCCCTTG
TAATATCGTTCGTAATCAATTTGTTTGTGACGACTGTTTTTGCTAAAGGTTTCTACGGGACAGAA
CTGGCCAATAGTATTGGCCTTGTAAATGCAGGGCAATATCTTCAAGACAAATACGGGGGTGGAT
TTTTCCCAATTTTATACATCTGGGGTATTGGATTATTAGCAGCTGGCCAAAGCAGCACCATTACT
GGCACTTATGCAGGACAGTTTATCATGGGAGGTTTCCTGAACTTGAGGTTAAAGAAATGGCTAA
GGGCATTGATCACTCGAAGCTGTGCTATCATCCCAACTATGATTGTTGCACTTGTTTTTGATACCT
CCGAAGACTCACTAGATGTTCTGAATGAATGGCTAAATGTGCTGCAGTCAATACAGATTCCTTTT
GCACTCATCCCTCTTCTCTGCTTAGTATCCAAGGAGCAAATCATGGGCACTTTCAAAATCGGCCC
CATTCTTAAGATGGTAGCTTGGCTTGTGGCTGCCCTGGTGATGGTAATCAATGGTTACCTTTTGCT
CGACTTTTTCTTCAATGAAGTGACCGGAGTAGCGTTTACCACTGTAGTATGCGGTTTTACAGGTG
CATATGCTGCGTTTATAATTTATCTCATTTCTAGGGGCTTTACATGTTTCTCCCGGTGCTGTCCAT
CTAAACAGATAGAAGTAGAG

>P. nigra_NRAMP3.1_CDS
ATGCCTGTACCAGAAGAAGACCCACAACCTTTATTAAAAGACCAAGAAGAAACAGCTTATGATT
CTGACGGGAAAGTTCTTTCATTTGGGATTGATTATGACACCGAAAGCGGTGGCTCAACGGTGGT
GCCATCATTTTCATGGAGAAAATTATGGTTGTTCACTGGTCCTGGGTTTTTAATGTGCATTGCTTT
TTTGGACCCTGGCAATTTGGAAGGGGATCTTCAGGCTGGTGCAATTGCAGGGTATTCTTTGCTTT
GGCTTCTCTTATGGGCTACTGCTATGGGTTTGTTGGTGCAGTTGCTGTCAGCAAGGCTTGGAGTG
GCTACAGGAAGGCATTTAGCTGAGCTATGTAGAGAAGAGTATCCAACTTGGGCTCGAATGATTT
TGTGGATTATGGCTGAGTTGGCTTTGATTGGTGCTGATATACAAGAAGTTATTGGGAGTGCTATT
GCTATTCAGATTTTGAGTAATGGGGTTTTGCCTTTGTGGGCTGGTGTTATTATTACTGCTTCTGAT
TGCTTTATCTTCCTATTTCTTGAGAACTACGGTGTGAGGAAATTGGAGGCTGCTTTTGGGATTCTC
ATTGGAATAATGGCAGTGACATTTGCGTGGATGTTTGCTGATGCAAAACCCAGTGCCCCCGAACT
TTTTCTAGGCATCTTAATTCCAAAACTTAGCTCCAAAACAATAAAACAGGCTGTTGGAGTTGTGG
GTTGCATTATCATGCCTCACAATGTGTTCTTGCATTCTGCTCTTGTACAGTCAAGGGAGATTGACC
ACAATAAGAAAGGCCAGGTTCAAGAAGCTCTTAGATACTACTCCATAGAGTCAACTGCTGCCCT
TGCAATATCATTCATGATCAATTTGTTTGTGACGACCGTTTTTGCTAAAGGTTTTCACGGGACAG
AACTGGCCAATAGTATTGGCCTTGTAAATGCAGGGCAATATCTTCAAGACAAATACGGGGGTGG
GTTTTTCCCAATTTTATACATCTGGGGTATTGGGTTATTAGCAGCTGGCCAAAGCAGCACCATTA
CTGGCACTTATGCAGGGCAGTTTATCATGGGAGGTTTCCTGAACTTGGGGTTAAAGAAATGGCTG
AGGGCATTGATTACTCGAAGCTGTGCTATCATCCCAACTATAATTGTTGCACTTGTTTTTGATACT
TCTGAAGACTCGCTAGATGTTCTGAATGAATGGCTAAATATGCTTCAGTCTATTCAGATTCCTTTC
GCACTCATCCCTCTTCTTTGCTTGGTCTCGAAGGAGCAAATCATGGGCACTTTCACAGTTGGCCC
CATTCTTCAGATGGTTTCTTGGCTTGTAGCTGCCTTGGTGATGCTAATCAATGGTTACCTTTTGCT
TGACTTTTTCTCCAATGAAGTAACTGGAGTAGCGTTTACCACTGTGGTATGCGCTTTTACAGGAG
CATATGTTGCGTTTATAATTTATCTCATTTCTAGGGAAGTTACCATTTCCACTTGGTACTGTCCCA
CC

>P. nigra_NRAMP3.2_CDS
ATGCCTGTAGAAGAAAACCACCAACCTTTATTGCAAGAAGAAGAAGAAAGAGCTTATGATTCTG
ATGAGAAAGTGCTCATAATTGGGGTTGATTCTGACACGGAAAGCGGTAGCTCAACGGTGTTGCC
ACCGTTTTCATGGAAAAAGTTATGGTTGTTTACTGGTCCTGGGTTTTTAATGTCCATTGCGTTTTT

GGATCCTGGGAATTTGGAAGGGGATCTTCAGGCTGGTGCAATCGCAGGCTACTCTTTGCTTTGGC
TTCTTTTATGGGCTACTGCTATGGGGTTGTTGGTGCAGTTGCTTTCAGCGAGGCTTGGAGTGGCT
ACAGGGAGGCATTTAGCTGAGCTGTGTAGAGAAGAGTATCCAACCTGGGCTTCAATGGTTTTGT
GGATTATGGCTGAGTTGGCTTTGATTGGTGCTGATATACAAGAGGTTATTGGAAGTGCTATTGCT
ATTAAGATCTTGAGTAATGGGTTTGTGCCTTTGTGGGCTGGTGTTACTATTACTGCTTGTGATTGG
TTCATATTCCTATTTCTAGAGAACTACGGTGTGAGAAAATTGGAGGCTGTATTTGCGGTCCTTAT
TGGAATAATGGCAGTTACATTTGGATGGATGTTTGCAGATGCAAAACCCAGTGCCTCCGAACTTT
TTCTAGGTATCTTAATTCCAAAACTTAGCTCCAGAACAATACAACAGGCTGTTGGAGTTGTGGGT
TGCATTATCATGCCTCACAATGTGTTCTTGCATTCTGCTCTTGTACAGTCAAGGGAGATCGACCA
CAATAAGAAAGACCGGGTTCAAGAAGCTCTCAGATACTACTCCATAGAGTCAACCACTGCCCTT
GTAATATCGTTCGTAATCAATTTGTTTGTGACGACTGTTTTTGCTAAAGGTTTCTACGGGACAGA
ACTGGCCAATAGTATTGGCCTTGTAAATGCAGGGCAATATCTTCAAGACAAATACGGGGGTGGA
TTTTTCCCAATTTTATACATCTGGGGTATTGGATTATTAGCAGCTGGCCAAAGCAGCACCATTAC
TGGGACCTATGCAGGACAGTTTATCATGGGAGGTTTCCTGAACTTGAGGTTAAAGAAATGGCTA
AGGGCACTGATCACTCGAAGCTGTGCTATCATCCCAACTATGATTGTTGCACTTGTTTTTGATAC
CTCCGAAGACTCACTAGATGTTCTGAATGAATGGCTAAATGTGCTGCAGTCAATACAGATTCCTT
TTGCACTCATCCCTCTTCTCTGCTTAGTATCCAAGGAGCAAATCATGGGCACTTTCAAAATCGGC
CCCACTCTTCAGATGGTATCTTGGCTTGTGGCTGCCCTGGTGATGGTAATCAATGGTTACCTTTTA
CTCGACTTTTTCTTCAATGAAGTGACCGGAGTAGCGTTTACCACTGTAGTATGCGGTTTTACAGG
TGCATATGTTGCGTTTATAATTTATCTCATATCTAGGGGCTTTACGTGTTTCTCCCGGTGCTGTTC
ATCTAAACAGATAGAAGTAGAG

>P. deltoides_NRAMP3.1_CDS
ATGCCTTTACCAGAAGAAGACCCACAACCTTTATTAAAAGACCAAGAAGAAACAGCTTATGATT
CTGACGGGAAAGTCCTTTCATTTGGGATTGATTATGACACCGAAAGCGGTAGCTCAACGGTGGT
GCCATCATTTTCATGGAGAAAATTATGGTTGTTCACTGGTCCTGGGTTTTTAATGTGCATTGCTTT
TTTGGACCCTGGCAATTTGGAAGGGGATCTTCAGGCTGGTGCAATTGCAGGATATTCTTTGCTTT
GGCTTCTCTTATGGGCTACTGCTATGGGTTTGTTGGTGCAGTTGCTGTCAGCAAGGCTTGGAGTG
GCTACAGGAAGGCATTTGGCTGAGCTATGTAGAGAAGAGTATCCAACTTGGGCTCGAATGATTT
TGTGGATTATGGCTGAGTTGGCTTTGATTGGTGCTGATATACAAGAAGTTATTGGGAGTGCTATT
GCTATTCAGATTTTGAGTAATGGGGTTTTGCCTTTGTGGGCTGGTGTTATTATTACTGCTTCCGAT
TGCTTTATCTTCCTATTTCTTGAGAACTACGGTGTGAGGAAATTGGAGGCTGCTTTTGGGATTCTC
ATTGGAATAATGGCAGTGACATTTGCGTGGATGTTTGCTGATGCAAAACCCAGTGCCCCCGAACT
TTTTCTGGGCATTTTAATTCCAAAACTTAGCTCCAAAACAATAAAACAGGCTGTTGGAGTTGTGG
GTTGCATTATCATGCCTCACAATGTGTTCTTGCATTCTGCTCTTGTACAGTCAAGGGAGATTGACC
ACAATAAGAAAGGCCAGGTTCAAGAAGCTCTCAGATACTACTCCATAGAGTCAACTGCTGCCCT
TGCAATATCATTCATGATCAATTTGTTTGTGACGACCGTTTTTGCTAAAGGTTTTCATGGGACAG
AACTGGCCAATAGTATTGGCCTTGTAAATGCAGGGCAATATCTTCAAGATAAATACGGGGGTGG
ATTTTTCCCAATTTTATACATCTGGGGTATTGGGTTATTAGCAGCTGGCCAAAGTAGCACCATTA
CTGGCACTTATGCAGGGCAGTTTATCATGGGAGGTTTCCTGAACTTGGGGTTAAAGAAATGGCTG
AGGGCATTGATTACTCGAAGCTGTGCTATCATCCCAACTATAATTGTTGCACTTGTTTTTGATACT
TCTGAAGACTCACTAGATGTTCTGAATGAATGGCTAAATATGCTTCAGTCTATTCAGATTCCTTTT
GCACTCATCCCTCTTCTTTGCTTGGTCTCTAAGGAGCAAATCATGGGCACTTTCACAGTTGGCCCC
ATTCTTCAGATGGTTTCTTGGCTTGTAGCTGCCTTGGTGATGTTAATCAATGGTTACCTTTTGCTT
GACTTTTTCTCCAATGAAGTAACTGGAGTAGTGTTTACCACTGTGGTATGCGCTTTTACAGGAGC
ATATGTTACGTTTATAATTTATCTCATTTCTAGGGAAGTTACCATTTCCACTTGGTACTGTCCCAC
C

>P. deltoides_NRAMP3.2_CDS

ATGCCAGTAGAAGAAAACCACCAACCTTTATTGCAAGAAGAAGAAGAAAGAGCTTATGATTCTG
ATGAGAAAGTGCTCATAATTGGGGTTGATTCTGACACGGAAAGCGGTGGCTCAACGGTGTTGCC
ACCGTTTTCATGGAAAAAGTTATGGTTGTTTACTGGTCCTGGGTTTTTAATGTCCATTGCGTTTTT
GGATCCTGGAAATTTGGAAGGGGATCTTCAGGCTGGTGCAATCGCAGGCTACTCTTTGCTTTGGC
TTCTTTTATGGGCTACTGCTATGGGGTTGTTGGTGCAGTTGCTTTCAGCGAGGCTTGGAGTGGCT
ACAGGGAGGCATTTAGCTGAGCTGTGTAGAGAAGAGTATCCAACCTGGGCTTCAATGGTTTTGT
GGATTATGGCTGAGTTGGCTTTGATTGGTGCTGATATACAAGAGGTTATTGGAAGTGCTATTGCT
ATTAAGATCTTGAGTAATGGGTTTGTGCCTTTGTGGGCTGGTGTTACTATTACTGCTTGTGATTGC
TTCATCTTCCTATTTCTAGAGAACTACGGTGTGAGAAAATTGGAGGCTGTATTTGCGGTCCTTATT
GGAATAATGGCAGTTACATTTGGATGGATGTTTGCAGATGCAAAACCCAGTGCCTCCGAACTTTT
TCTGGGTATCTTAATTCCAAAACTTAGCTCCAGAACAATACAACAGGCTGTTGGAGTTGTGGGTT
GCATTATCATGCCTCACAATGTGTTCTTGCATTCTGCTCTTGTACAGTCAAGGGAGATTGACCAC
AATAAGAAAGGCCGGGTTCAAGAAGCTCTCAGATACTACTCCATAGAGTCAACCACTGCCCTTG
TAATATCGTTCGTAATCAATTTGTTTGTGACGACTGTTTTTGCTAAAGGTTTCTACGGGACAGAA
CTGGCCAATAGTATTGGCCTTGTAAATGCAGGGCAATATCTTCAAGACAAATACGGGGGTGGAT
TTTTCCCAATTTTATACATCTGGGGTATTGGATTATTAGCAGCTGGCCAAAGCAGCACCATTACT
GGCACTTATGCAGGACAGTTTATCATGGGAGGTTTCCTGAACTTGAGGTTAAAGAAATGGCTAA
GGGCATTGATCACTCGAAGCTGTGCTATAATCCCAACTATGATTGTTGCACTTGTTTTTGATACCT
CCGAAGACTCACTAGATGTTCTGAATGAATGGCTAAATGTGCTGCAGTCAATACAGATTCCTTTT
GCACTCATCCCTCTTCTCTGCTTAGTATCCAAGGAGCAAATCATGGGCACTTTCAAAATCGGCCC
CATTCTTCAGATGGTAGCTTGGCTTGTGGCTGCCCTGGTGATGGTAATCAATGGTTACCTTTTACT
CGACTTTTTCTTCAATGAAGTGACCGGAGTAGCGTTTACCACTGTAGTATGCGGTTTTACAGGTG
CATATGCTGCGTTTATAATTTATCTCATATCTAGGGGCTTTACATGTTTCTCCCGGTGCTGTCCAT
CCAAACAGATAGAAGTAGAG

>P. tremula_NRAMP3.1_CDS
ATGCCTTTACCAGAAGAAGACCCACAACCTTTATTAAAAGACCAAGAAGAAACAGCTCATGATT
CTGACGGGAAAGTCCTTTCATTTGGGATTGATTATGACACCGAAAGCGGTGGCTCAACGGTGGT
GCCATCATTTTCATGGAGAAAATTATGGTTGTTCACTGGTCCTGGGTTTTTAATGTGCATTGCTTT
TTTGGACCCTGGCAATTTGGAAGGGGATCTTCAGGCTGGTGCAATTGCAGGGTATTCTTTGCTTT
GGCTTCTCTTATGGGCTACTGCTATGGGTTTGTTGGTGCAGTTGCTGTCAGCAAGGCTTGGAGTG
GCTACAGGAAGGCATTTGGCTGAGCTATGTAGAGAAGAGTATCCAACCTGGGCTCGAATGATTT
TGTGGATTATGGCTGAGTTGGCTTTGATTGGTGCTGATATACAAGAAGTTATTGGGAGTGCTATA
GCTATTCAGATTTTGAGTAATGGGGTTTTGCCTTTGTGGGCTGGTGTTATTATTACTGCTTCCGAT
TGCTTTATCTTCCTATTTCTTGAGAACTACGGTGTGAGGAAACTGGAGGCTGCTTTTGGGATCCTC
ATTGGAATAATGGCAGTGGCATTTGCGTGGATGTTTGCTGATGCAAAACCCAGTGCCCCTGAACT
TTTTCTGGGCATCTTAATTCCAAAACTTAGCTCCAAAACAATAAAACAGGCTGTCGGAGTTGTGG
GTTGCATTATCATGCCTCACAATGTGTTCTTGCATTCTGCTCTTGTACAGTCAAGGGAGATTGACT
GCAATAAGAAAGGCCAGGTTCAAGAAGCTCTCAGATACTACTCCATAGAGTCAACTGCTGCCCT
TGCAATATCATTCATGATCAATTTGTTTGTGACGACCATTTTTGCTAAAGGTTTCCACGGGACAG
AACTGGCCAATAGTATTGGCCTTGTAAATGCAGGGCAATATCTTCAAGATAAATACGGGGGTGG
ATTTTTCCCAATTTTATACATCTGGGGTATTGGGTTATTAGCAGCTGGCCAAAGTAGCACCATTA
CTGGCACTTATGCAGGGCAGTTTATCATGGGAGGTTTCCTGAACTTGGGATTAAAGAAATGGCTG
AGGGCATTGATTACTCGAAGCTGTGCTATCATCCCAACTATAATTGTTGCACTTGTTTTTGATACT
TCTGAAGACTCACTAGATGTTCTGAATGAATGGCTAAATATGCTTCAGTCTATTCAGATTCCTTTT
GCACTCATCCCTCTTCTTTGCTTGGTCTCCAAGGAGCAAATCATGGGCACTTTCACAGTCGGCCC
CATTCTTAAGATGGTTTCTTGGCTTGTAGCTGCCTTGGTGATGCTAATCAATGGTTACCTTTTGCT
TGACTTTTTCTCCAATGAAGTAACTGGAGTAGTGTTTACCACTGTGGTATGCGCTTTTACAGGAG
CATATGTTACGTTTATAATTTATCTCATTTCTAGGGAAGTATCCATTTCCACTTGGTACTGTCCCA
CC

>P. tremula_NRAMP3.2_CDS
ATGTCTGTAGAAGAAAACCAACAACCTTTATTGCAAGAAGAAGAAGAAAGAGCTTATGATTCTG
ATGAGAAAGTGCTCATAATTGGGGTTGATTCTGACACGGAAAGCGGTGGCTCAACGGTGTTGCC
ACCGTTTTCATGGAAAAAGTTATGGTTGTTTACTGGTCCTGGATTTTTAATGTCCATTGCGTTTTT
GGATCCTGGGAATTTGGAAGGGGATCTTCAGGCTGGTGCAATCGCAGGCTACTCTTTGCTTTGGC
TTCTTTTCTGGGCTACTGCTATGGGGTTGTTGGTGCAGTTGCTTTCAGCGAGGCTTGGAGTGGCTA
CAGGGAGGCATTTAGCTGAGCTGTGTAGAGAAGAGTATCCAACCTGGGCTTCAATGGTTTTGTG
GATTATGGCTGAGTTGGCTTTGATTGGTGCTGATATACAAGAGGTTATTGGAAGTGCTATTGCTC
TTAAGATCTTGAGTAATGGGTTTTTGCCTTTGTGGGCTGGTGTTACTATTACTGCTTGTGATTGCT
TCATCTTCCTATTTCTAGAGAACTACGGTGTGAGAAAATTGGAGGCTGTATTTGCGGTCCTTATT
GGAATAATGGCAGTTACATTTGGATGGATGTTTGCAGATGCAAAACCCAGTGCCTCCGAACTTTT
TCTGGGTATCTTAATTCCCAAACTTAGCTCCAGAACAATACAACAGGCTGTTGGAGTTGTGGGTT
GCATTATCATGCCTCACAATGTGTTCTTGCATTCTGCTCTTGTACAGTCAAGGGAGATCGACCAC
AATAAGAAAGGCCGGGTTCAAGAAGCTCTCAGATACTACTCCATAGAGTCAACCACTGCCCTTG
TAATATCGTTCGTAATCAATTTGTTTGTGACGACTGTTTTTGCTAAAGGTTTCTATGGGACAGAAC
TGGCCAATAGTATTGGCCTTGTAAATGCAGGGCAATATCTTCAAGACAAATACGGGGGTGGATT
TTTCCCAATTTTATACATCTGGGGCATTGGATTATTAGCAGCTGGCCAAAGCAGCACCATTACTG
GCACTTATGCAGGGCAGTTTATCATGGGAGGTTTCCTGAACTTGAGGTTAAAGAAATGGTTAAG
GGCATTGATCACTCGAAGCTGTGCTATCATCCCAACTATGATTGTTGCACTTGTTTTTGATGCCTC
CGAAGACTCACTAGATGTTCTGAATGAATGGCTAAATGTGCTGCAATCAATACAGATTCCTTTTG
CACTCATTCCTCTTCTCTTCTTGGTATCCAAGGAGCAAATCATGGGCACTTTCAAAATCGGTCCC
ATTCTTAAGATGGTATCTTGGCTTGTGGCTGCCCTGGTGATAGTAATCAATGGTTACCTTTTGCTC
GACTTTTTCGTCAATGAAGTGACCGGAGTAGCGTTTACCACTGTAGTATGCGGTTTTACAGGTGC
ATATGTTGCGTTTATAATTTATCTCATTTCTAGGGGCTTTAAATGTTTCTCCTGGTGCTGTCAATC
TAAACAGATAGAAGTAGAG

>P. tremuloides_NRAMP3.1_CDS
ATGCCTTTACCAGAAGAAGACCCACAACCTTTATTAAAAGACCAAGAAGAAACAGCTTATGATT
CTGACGGGAAAGTCCTTTCATTTGGGATTGATTATGACACCGAAAGCGGTGGCTCAACGGTGTTG
CCACCGTTTTCATGGAAAAAGTTATGGTTGTTTACTGGTCCTGGGTTTTTAATGTCCATTGCTTTT
TTGGACCCTGGCAATTTGGAAGGGGATCTTCAGGCTGGTGCAATTGCAGGGTATTCTTTGCTTTG
GCTTCTCTTATGGGCTACTGCTATGGGTTTGTTGGTGCAGTTGCTGTCAGCAAGGCTTGGAGTGG
CTACAGGAAGGCATTTGGCTGAGCTATGTAGAGAAGAGTATCCAACCTGGGCTCGAATGATTTT
GTGGATTATGGCTGAGTTGGCTTTGATTGGTGCTGATATACAAGAAGTTATTGGGAGTGCTATAG
CTATTCAGATTTTGAGTAATGGGGTTTTGCCTTTGTGGGCTGGTGTTATTATTACTGCTTCCGATT
GCTTTATCTTCCTATTTCTTGAGAACTACGGTGTGAGGAAATTGGAGGCTGCTTTTGGGATCCTC
ATTGGAATAATGGCAGTGACATTTGCGTGGATGTTTGCTGATGCAAAACCCAGTGCCCCTGAACT
TTTTCTGGGCATCTTAATTCCAAAACTTAGCTCCAAAACAATAAAACAGGCTGTCGGAGTTGTGG
GTTGCATTATCATGCCTCACAATGTGTTCTTGCATTCTGCTCTTGTACAGTCAAGGGAGATTGACC
ACAATAAGAAAGGCCAGGTTCAAGAAGCTCTCAGATACTACTCCATAGAGTCAACTGCTGCCCT
TGCAATATCATTCATGATCAATTTGTTTGTGACGACCATTTTTGCTAAAGGTTTCCACGGGACAG
AACTGGCCAATAGTATTGGCCTTGTAAATGCAGGGCAATATCTTCAAGATAAATACGGGGGTGG
ATTTTTCCCAATTTTATACATCTGGGGTATTGGGTTATTAGCAGCTGGCCAAAGTAGCACCATTA
CTGGCACTTATGCAGGGCAGTTTATCATGGGAGGTTTCCTGAACTTGGGATTAAAGAAATGGCTG
AGGGCATTGATTACTCGAAGCTGTGCTATCATCCCAACTATAATTGTTGCACTTGTTTTTGATACC
TCTGAAGACTCACTAGATGTTCTGAATGAATGGCTAAATATGCTTCAGTCTATTCAGATTCCTTTT
GCACTCATCCCTCTTCTTTGCTTGGTCTCCAAGGAGCAACTCATGGGCACTTTCACAGTTGGCCCC
ATTCTTAAGATGGTTTCTTGGCTTGTAGCTGCCTTGGTGATGCTAATCAATGGTTACCTTTTGCTT
GACTTTTTCTCCAATGAAGTAACTGGAGTATTGTTTACCACTGTGGTATGCGCTTTTACAGGAGC
ATATGTTACGTTTATAATTTATCTCATTTCTAGGGAAGTATCCATTTCCACTTGGTACTGTCCCAC
C

>P. tremuloides_NRAMP3.2_CDS
ATGCCTGTAGAAGAAAACCAACAACCTTTATTGCAAGAAGAAGAAGAAAGAGCTTATGATTCTG
ATGAGAAAGTGCTCATAATTGGGGTTGATTCTGACACGGAAAGCGGTGGCTCAACGGTGTTGCC
ACCGTTTTCATGGAAAAAGTTATGGTTGTTTACTGGTCCTGGGTTTTTAATGTCCATTGCGTTTTT
GGATCCTGGGAATTTGGAAGGGGATCTTCAGGCTGGTGCAATCGCAGGCTACTCTTTGCTTTGGC
TTCTTTTATGGGCTACTGCTATGGGGTTGTTGGTGCAGTTGCTTTCAGCGAGGCTTGGAGTGGCT
ACAGGGAGGCATTTAGCTGAGCTGTGTAGAGAAGAGTATCCAACCTGGGCTTCAATGGTTTTGT
GGATTATGGCTGAGTTGGCTTTGATTGGTGCTGATATACAAGAGGTTATTGGAAGTGCTATTGCT
CTTAAGATCTTGAGTAATGGGTTTTTGCCTTTGTGGGCTGGTGTTACTATTACTGCTTGTGATTGC
TTCATCTTCCTATTTCTAGAGAACTACGGTGTGAGAAAATTGGAGGCTGTATTTGCGGTCCTTATT
GGACTAATGGCAGTTACATTTGGATGGATGTTTGCAGATGCAAAACCCAGTGCCTCCGAACTTTT
TCTGGGTATCTTAATTCCCAAACTTAGCTCCAGAACAATACAACAGGCTGTTGGAGTTGTGGGTT
GCATTATCATGCCTCACAATGTGTTCTTGCATTCTGCTCTTGTACAGTCAAGGGAGATCGACCAC
AATAAGAAAGGCCGGGTTCAAGAAGCTCTCAGATACTACTCCATAGAGTCAACCACTGCCCTTG
TAATATCGTTCGTAATCAATTTGTTTGTGACGACTGTTTTTGCTAAAGGTTTCTATGGGACAGAAC
TGGCCAATAGTATTGGCCTTGTAAATGCAGGGCAATATCTTCAAGACAAATACGGGGGTGGATT
TTTCCCAATTTTATACATCTGGGGTATTGGATTATTAGCAGCTGGCCAAAGCAGCACCATTACTG
GCACTTATGCAGGACAGTTTATCATGGGAGGTTTCCTGAACTTGAGGTTAAAGAAATGGTTAAG
GGCATTGATCACTCGAAGCTGTGCTATCATCCCAACTATGATTGTTGCACTTGTTTTTGATGCCTC
CGAAGACTCACTAGATGTTCTGAATGAATGGCTAAATGTGCTGCAGTCAATACAGATTCCTTTTG
CACTCATCCCTCTTCTCTGCTTGGTATCCAAGGAGCAAATCATGGGCACTTTCAAAATCGGCCCC
ATTCTTAAGATGGTATCTTGGCTTGTGGCTGCCCTGGTGATAGTAATCAATGGTTACCTTTTGCTC
GACTTTTTCGTCAATGAAGTGGCCGGAGTAGCGTTTACCACTGTAGTATGCGGTTTTACAGGTGC
ATATGTTGCGTTTATAATTTATCTCATTTCTAGGGGCTTTACATGTTTCTCCTGGTGCTGTCCATCT
AAACAGATAGAAGTAGAG

>P. grandidentata_NRAMP3.1_CDS
ATGCCTTTACCAGAAGAAGACCCACAACCTTTATTAAAAGACCAAGAAGAAACAGCTTATGATT
CTGACGGGAAAGTCCTTTCATTTGGGATTGATTATGACACCGAAAGCGGTGGCTCAACGGTGGT
GCCATCATTTTCATGGAGAAAATTATGGTTGTTCACTGGTCCTGGGTTTTTAATGTGCATTGCTTT
TTTGGACCCTGGCAATTTGGAAGGGGATCTTCAGGCTGGTGCAATTGCAGGGTATTCTTTGCTTT
GGCTTCTCTTATGGGCTACTGCTATGGGTTTGTTGGTGCAGTTGCTGTCAGCAAGGCTTGGGGTG
GCTACAGGAAGGCATTTGGCTGAGCTATGTAGAGAAGAGTATCCAACTTGGGCTCGAATGATTT
TGTGGATTATGGCTGAGTTGGCTTTGATTGGTGCTGATATACAAGAAGTTATTGGGAGTGCTATT
GCCATTCAGATTTTGAGTAATGGGGTTTTGCCTTTGTGGGCTGGTGTTATTATTACTGCTTCCGAT
TGCTTTATCTTCCTATTTCTTGAGAACTACGGTGTGAGGAAATTGGAGGCTGCTTTTGGGATCCTC
ATTGGAATAATGGCAGTGACATTTGCGTGGATGTTTGCTGATGCAAAACCCAGTGCCCCTGAACT
TTTTCTGGGCATCTTAATTCCAAAACTTAGCTCCAAAACAATAAAACAGGCTGTCGGAGTTGTGG
GTTGCATTATCATGCCTCACAATGTGTTCTTGCATTCTGCTCTTGTACAGTCAAGGGAGATTGACC
ACAATAAGAAAGGCCAGGTTCAAGAAGCTCTCAGATACTACTCCATAGAGTCAACTGCTGCCCT
TGCAATATCATTCATGATCAATTTGTTTGTGACGACCATTTTTGCTAAAGGTTTCCACGGGACAG
AACTGGCCAATAGTATTGGCCTTGTAAATGCAGGGCAATATCTTCAAGATAAATACGGGGGTGG
ATTTTTCCCAATTTTATACATCTGGGGTATCGGGTTATTAGCAGCTGGCCAAAGTAGCACCATTA
CTGGCACTTATGCAGGGCAGTTTATCATGGGAGGTTTCCTGAACTTGGGATTAAAGAAATGGCTG
AGGGCATTGATTACTCGAAGCTGTGCTATCATCCCAACTATAATTGTTGCACTTGTTTTTGATACT
TCTGAAGACTCACTAGATGTTCTGAATGAATGGCTAAATATGCTTCAGTCTATTCAGATTCCTTTT
GCACTCATCCCTCTTCTTTGCTTGGTCTCCAAGGAGCAACTCATGGGCACTTTTACAGTTGGCCCC
ATTCTTAAGATGGTTTCTTGGCTTGTAGCTGCCTTGGTGATGCTAATCAATGGTTACCTTTTGCTT
GACTTTTTCTCCAATGAAGTAACTGGAGTAGTGTTTACCACTGTGGTATGCGCTTTTACAGGAGC

GTATGTTACGTTTATAATTTATCTCATTTCTAGGGAAGTATCCATTTCCACTTGGTACTGTCCCAC
C

>P. grandidentata_NRAMP3.2_CDS
ATGCCTGTAGAAGAAAACCAACAACCTTTATTGCAAGAAGAAGAAGAAAGAGCTTATGATTCTG
ATGAGAAAGTGCTCATAATTGGGGTTGATTCTGACACGGAAAGCGGTGGCTCAACGGTGTTGCC
ACCGTTTTCATGGAAAAAGTTATGGTTGTTTACTGGTCCTGGGTTTTTAATGTCAATTGCGTTTTT
GGATCCTGGGAATTTGGAAGGGGATCTTCAGGCTGGTGCAATCGCAGGCTACTCTTTGCTTTGGC
TTCTTTTATGGGCTACTGCTATGGGGTTGTTGGTGCAGTTGCTTTCAGCGAGGCTTGGAGTGGCT
ACAGGGAGGCATTTAGCTGAGCTGTGTAGAGAAGAGTATCCAACCTGGGCTTCAATGATTTTGT
GGATTATGGCTGAGTTGGCTTTGATTGGTGCTGATATACAAGAGGTTATTGGAAGTGCTATTGCT
CTTAAGATCTTGAGTAATGGGTTTTTGCCTTTGTGGGCTGGTGTTACTATTACTGCTTGTGATTGC
TTCATCTTCCTATTTCTAGAGAACTACGGTGTGAGAAAATTGGAGGCTGTATTTGCGGTCCTTATT
GGAATAATGGCAGTTACATTTGGATGGATGTTTGCAGATGCAAAACCCAGTGCCTCCGAACTTTT
TCTGGGTATCTTAATTCCCAAACTTAGCTCCAGAACAATACAACAGGCTGTTGGAGTTGTGGGTT
GCATTATCATGCCTCACAATGTGTTCTTGCATTCTGCTCTTGTACAGTCAAGGGAGATCGACCAC
AATAAGAAAGGCCGGGTTCAAGAAGCTCTCAGATACTACTCCATAGAGTCAACCACTGCCCTTG
TAATATCGTTCGTAATCAATTTGTTTGTGACGACTGTTTTTGCTAAAGGTTTCTATGGGACAGAAC
TGGCCAATAGTATTGGCCTTGTAAATGCAGGGCAATATCTTCAAGACAAATACGGGGGTGGATT
TTTCCCAATTTTATACATCTGGGGTATTGGATTATTAGCAGCTGGCCAAAGCAGCACCATTACTG
GCACTTATGCAGGACAGTTTATCATGGGAGGTTTCCTGAACTTGAGGTTAAAGAAATGGTTAAG
GGCATTGATCACTCGAAGCTGTGCTATCATCCCAACTATGATTGTTGCCCTTGTTTTTGATGCCTC
CGAAGACTCACTAGATGTTCTGAATGAATGGCTAAATGTGCTGCAGTCAATACAGATTCCTTTTG
CACTCATCCCTCTTCTCTGCTTGGTATCCAAGGAGCAAATCATGGGCACTTTCAAAATCGGCCCC
ATTCTTAAGATGGTATCTTGGCTTGTGGCTGCCCTGGTGATAGTAATCAATGGTTACCTTTTGCTC
GACTTTTTCGTCAATGAAGTGGCCGGAGTAGCGTTTACCACTGTAGTATGCGGTTTTACAGGTGC
ATATGTTGCATTTATAATTTATCTCATTTCTAGGGGCTTTACATGTTTCTCCCGGTGCTGTCCATCT
AAACAGATAGAAGTAGAG

>S. purpurea_NRAMP3_CDS
ATGTCTTTAGATGAAAACCAGCAACCTTTATTGCAAGAAGAAGAAGAGAGAGCTTATGATTCTG
ATGAGAAAGTGCTCGTAATTGGGATTGATTCTGACGCCGAAAGCGGTGGAACGGTGTTGCCACC
GTTTTCATGGAAAAAGTTATGGTTGTTTACTGGTCCTGGGTTTTTAATGTCCATTGCGTTTTTGGA
TCCTGGAAATTTGGAAGGGGATCTTCAGGCTGGTGCAATTGCAGGCTACTCTTTGCTTTGGCTCC
TTTTTTGGGCCACTGCTATGGGGTTGTTGGTGCAGTTGCTGTCAGCCAGGCTTGGAGTGGCTACA
GGGAGGCATTTAGCTGAGCTGTGTAGAGAAGAGTATCCAACCTGGGCTAGAATGATTTTGTGGA
TTATGGCTGAGTTAGCTTTGATTGGTGCTGATATACAAGAGGTTATTGGAAGTGCTATTGCTATT
AAGATCTTGAGTAACGGGGTTGTGCCTTTATGGGCTGGTGTTACTATTACTGCTTGTGACAGCTT
CATCTTCCTATTTCTAGAGAACTACGGTGTGAGAAAATTGGAGGCTGTATTTGCGGTCCTCATTG
GAGTCATGGCAGTTACATTTGGATGGATGTTTGCTGATGCCAAACCCAGTGCCTCCGAACTTTTT
CTGGGTATCTTTATTCCAAAACTTAGCTCCAGAACAATACAGCAGGCTGTAGGAGTTGTGGGTTG
CATTATCATGCCTCACAATGTGTTCCTGCATTCTGCTCTTGTACAGTCAAGGGAGATCGACCACA
ATAATAAAGTCCAGGTTCAAGAAGCTCTCAGATACTACTCCATAGAGTCAACCACTGCCCTTGTG
ATATCGTTCGTAATCAATTTGTTCGTGACGACTGTGTTTGCTAAAGGTTTCTATGGGACAGAACT
GGCCAATAGTATTGGCCTTGTAAATGCAGGGCAATATCTTCAAGACAAATACGGGGGTGGATTT
TTCCCAATCTTATACATCTGGGGTATTGGATTATTAGCTGCTGGCCAAAGTAGCACCATTACTGG
GACCTATGCAGGACAGTTTATAATGGGAGGTTTCCTGAACATGAGGTTAAAGAAATGGCTAAGG
GCATTGATCACTCGAAGTTGTGCTATCATCCCAACGATAATTGTTGCTCTTATTTTTGATACCTCT
GAAGATTCTCTAGATGTTCTGAATGAATGGCTAAATGTGCTGCAGTCAATACAGATTCCTTTTGC
ACTCATCCCTCTTCTCTGTTTGGTCTCCAAGGAGCGAATCATGGGCACTTTCAAAATTGGCTCCAT

CCTTAAGGTCGTTTCTTGGCTTGTGGCGGCCCTGGTGATAGTAATCAATGGTTACCTTTTGCTCGA
CTTTTTCTTCAATGAAGTGACTGGAGTAGCGTTTACCACTGTAGTATGCACTTTTACAGCTGCATA
TGCTGCGTTTATAATTTATCTCACTTCCAGGGGCGTTACATGTTCTTCCTGGCGCGGCCCACCTAA
ACAGATAGAAGTAGAG

>S. suchowensis_NRAMP3_CDS
ATGTCTTTAGATGAAAACCAGCAACCATTATTGCAAGAAGAAGAAGAGAGAGCTTATGATTCTG
ATGAGAAAGTGCTCGTAATTGGGATTGATTCTGACGCCGAAAGCGGTAGAACGGTGTTGCCACC
GTTTTCATGGAAAAAGTTATGGTTGTTTACTGGTCCTGGGTTCTTAATGTCCATTGCGTTTTTGGA
TCCTGGAAATTTGGAAGGGGATCTTCAGGCTGGTGCAATTGCAGGCTACTCTTTGCTTTGGCTCC
TTTTATGGGCCACTGCTATGGGGTTGTTGGTGCAGTTGCTGTCTGCCAGGCTTGGAGTGGCTACA
GGGAGGCATTTAGCTGAGCTGTGTAGAGAAGAGTATCCAACCTGGGCTAGAATGATTTTGTGGA
TTATGGCTGAGTTAGCTTTGATTGGTGCTGATATACAAGAGGTTATTGGAAGTGCTATTGCTATT
AAGATCTTGAGTAACGGGGTTGTGCCTTTATGGGCTGGTGTTACTATTACTGCTTGTGATTGCTTC
ATCTTCCTATTTCTAGAGAACTACGGTGTGAGAAAATTGGAGGCTGTATTTGCGGTCCTCATTGG
AGTCATGGCAGTTACATTTGGATGGATGTTTGCTGATGCCAAACCCAGTGCCTCCGAACTTTTTC
TGGGTATCTTAATTCCAAAACTTAGCTCCAGAACAATACAGCAGGCTGTAGGAGTTGTGGGTTGC
ATTATCATGCCTCACAATGTGTTCCTGCATTCTGCTCTTGTACAGTCAAGGGAGATCGACCACAA
TAATAAAGTCCAGGTTCAAGAAGCTCTCAGATACTACTCCATAGAGTCAACCACTGCCCTTGTGA
TATCGTTCGTAATCAATTTGTTCGTGACGACTGTGTTTGCTAAAGGTTTCTATGGGACAGAACTG
GCCAATAGTATTGGCCTTGTAAATGCAGGGCAATATCTTCAAGACAAATACGGGGGTGGATTTTT
TCCAATCTTATACATCTGGGGTATTGGATTATTAGCTGCTGGCCAAAGTAGCACCATTACTGGGA
CGTATGCAGGACAGTTTATCATGGGAGGTTTCCTGAACATGAGGTTAAAGAAATGGCTAAGGGC
ATTGATCACTCGAAGTTGTGCTATCATCCCAACGATAATTGTTGCGCTTATTTTTGATACCTCTGA
AGATTCTCTAGATGTTCTGAATGAATGGCTAAATGTGCTGCAGTCAATACAGATTCCTTTTGCAC
TCATCCCTCTTCTCTGTTTGGTCTCCAAGGAGCGAATCATGGGCACTTTCAAAATTGGCTCCATCC
TTAAGGTCGTTTCTTGGCTTGTGGCGGCCCTGGTGATAGTAATCAATGGTTACCTTTTGCTCGACT
TTTTCTTCAATGAAGTGACTGGAGTAGCGTTTACCACTGCAGTATGCACTTTCACAGCTGCATAT
GCTGCGTTTATAATTTACCTCACTTCCAGGGGCATTACATGTTCTTCCTGGCGCGGCCCACCTAA
ACAGATAGAAGCAGAG

>S. brachista_NRAMP3_CDS
ATGTCTTTAGATGAAAACCAGCAACCATTATTGCAAGAAGAAGAAGAGAGAGCTTATGATTCTG
ATCAGAAAGTGCTCGTAATTGGGATTGATTCTGACGCCGAAAGCGGTGGCACGGTGTTACCACC
GTTTTCATGGAAAAAGTTATGGTTGTTTACTGGTCCTGGGTTTTTAATGTCCATTGCGTTTTTGGA
TCCTGGAAATTTGGAAGGGGATCTTCAGGCTGGTGCGATTGCAGGCTACTCTTTGCTTTGGCTCC
TTTTATGGGCCACTGCTATGGGGTTGTTGGTGCAGTTGCTGTCAGCCAGGCTTGGAGTGGCTACA
GGGAGGCATTTAGCTGAGCTGTGTAGAGAAGAGTATCCAACCTGGGCTAGAATGATTTTGTGGA
TTATGGCTGAGTTAGCTTTGATTGGTGCTGATATACAAGAGGTTATTGGAAGTGCTATTGCTATT
AAGATCTTGAGTAACGGGGTTGTGCCTTTATGGGCTGGTGTTACTATTACTGCTTGTGATTGCTTC
ATCTTCCTATTTCTAGAGAACTACGGTGTGAGAAAATTGGAGGCTGTATTTGCGGTCCTAATTGG
AGTAATGGCAGTTACATTTGGAATGATGTTTGCTGATGCCAAACCCAGTGCCTCCGAACTTTTTC
TGGGTATCTTAATCCCAAAACTTAGCTCCAGAACAATACAACAGGCTGTAGGAGTTGTGGGTTG
CATTATCATGCCTCACAATGTGTTCCTGCATTCTGCTCTTGTACAGTCAAGGGAGATCGACCACA
ATAATAAAGTCCAGGTTCAAGAAGCTCTCAGATACTACTCCATAGAGTCAACCACTGCCCTTGTA
ATATCGTTCGTAATCAATTTGTTCGTGACGACTGTGTTTGCTCAAGGTTTCTATGGGACAGAACT
GGCCAATAGTATTGGCCTTGTAAATGCAGGGCAATATCTTCAAGACAAATACGGGGGTGGATTT
TTCCCAATCTTATACATCTGGGGTATTGGATTATTAGCTGCTGGCCAAAGTAGCACCATTACAGG
GACCTATGCAGGACAGTTTATCATGGGAGGTTTCCTGAACATGAGGTTAAAGAAATGGCTGAGG
GCATTGATCACTCGAAGTTGTGCTATCATCCCAACGATAATTGTTGCGCTTGTTTTTGAAACCTCT

GAAGAGTCTCTAGATGTTCTGAATGAATGGCTAAATGTGCTGCAGTCAATACAGATTCCTTTTGC
ACTCATCCCTCTTCTCTGTTTGGTCTCCAAGGAGCGAATCATGGGCACTTTCAAAATTGGCTCCAT
TCTTAAGGTCGTTTCTTGGCTTGTGGCGGCCCTGGTGATAATAATCAATGGTTACCTTTTGCTCGA
CTTTTTCTTCAATGAAGTGACTGGAGTAGCGTATACCACTGCAGTATGCACTTTTACAGCTGCAT
ATGCTGCGTTTATAATTTATCTCACTTCTAGGGGCGTTACATGCTCTTCCTGGCGCGGCCCACCTA
AACAGAATCAACAGATAGAAGTAGAG

>S. eriocephala_NRAMP3_CDS
ATGTCTTTAGATGAAAACCAGCAAGCTTTATTGCAAGAAGAAGAAGAGAGAGCTTATGATTCTG
ATGAGAAAGTGCTCGTAATTGGGGTTTATTCTGACGCCGAAAGCGGTGGCACGGTGTTGCCACC
GTTTTCATGGAAAAAGTTATGGTTGTTTACTGGTCCTGGGTTCTTAATGTCCATTGCGTTTTTGGA
TCCTGGAAATTTGGAAGGGGATCTTCAGGCTGGTGCAATTGCAGGCTACTCTTTGCTTTGGCTCC
TCTTATGGGCTACTGCTATGGGGTTGTTGGTGCAGTTGCTGTCAGCCAGGCTTGGAGTGGCTACA
GGGAGGCATTTAGCTGAGCTGTGTAGAGAAGAGTATCCAACCTGGGCTAGAATGATTTTGTGGA
TTATGGCTGAGTTAGCTTTGATTGGAGCTGATATACAAGAGGTTATTGGAAGTGCTATTGCTATT
AAGATCTTGAGTAACGGGGTTGTGCCTTTATGGGCTGGTGTTACTATTACTGCTTGTGATTGCTTC
ATCTTCCTATTTCTAGAGAACTACGGTGTGAGAAAATTGGAGGCTGTATTTGCGGTCCTAATTGG
AGTCATGGCAGTTACATTTGGATGGATGTTTGCTGATGCGAAACCCAGTGCCTCCGAACTTTTTC
TGGGTATCTTAATTCCAAAACTTAGCTCCAGAACAATACAGCAGGCTGTAGGAGTTGTGGGTTGC
ATTATCATGCCTCACAATGTGTTCCTGCATTCTGCTCTTGTACAGTCAAGGGAGATCGACCACAA
TAATAAAATCCAGGTTCAAGAAGCTCTCAGATACTACTCCATAGAGTCAACCACTGCCCTTGTAA
TATCGTTCGTAATCAATTTGTTCGTGACGACTGTGTTTGCTAAAGGTTTCTATGGGACAGAACTG
GCCAATAGTATTGGCCTTGTAAATGCAGGGCAATATCTTCAAGACAAATACGGGGGTGGATTTTT
CCCAATCTTATACATCTGGGGTATTGGATTATTAGCTGCTGGCCAAAGTAGCACCATTACTGGGA
CCTATGCAGGACAGTTTATCATGGGAGGTTTCCTGAACATGAGGTTAAAGAAATGGATAAGGGC
ATTGATCACTCGAAGTTGTGCTATCATCCCAACGATAATTGTTGCGCTTATTTTTGATACCTCTGA
AGATTCTCTAGATGTTCTGAATGAATGGCTAAATGTGCTGCAGTCAATACAGATTCCTTTTGCAC
TCATCCCTCTTCTCTGTTTGGTCTCCAAGGAGCGAATCATGGGCACTTTCAAAATTGGCTCCATCC
TTAAGGTCGTTTCTTGGCTTGTGGCGGCCCTGGTGATGGTAATCAATGGTTACCTTTTGCTCGACT
TTTTCTTCAATGAAGTGACCGGAGTAGCGTTTACCACTGCAGTATGCACTTTTACAGCTGCATAT
GCTGCGTTTATAATTTATCTCACTTCCAGGGGCGTTACATGTTCTTCCTGGCGCGGCCCACCTAAA
CAGATAGAAGTAGAG

>S. sachalinensis_NRAMP3_CDS
ATGTCTTTAGATGAAAACCAGCAACCTTTATTGCAAGAAGAAGAAGAGAGAGCTTATGATTCTG
ATGAGAAAGTGCTCGTAATTGGGATTGATTCTGACGCCGAAAGCGGTGGAACGGTGTTGCCACC
GTTTTCATGGAAAAAGTTATGGTTGTTTACTGGTCCTGGGTTTTTAATGTCCATTGCGTTTTTGGA
TCCTGGAAATTTGGAAGGGGATCTTCAGGCTGGTGCAATTGCAGGCTACTCTTTGCTTTGGCTCC
TTTTATGGGCCACTGCTATGGGGTTGTTGGTGCAGTTGCTGTCAGCCAGGCTTGGAGTGGCTACA
GGGAGGCATTTAGCTGAGCTGTGTAGAGAAGAGTATCCAACCTGGGCTAGAATGATTTTGTGGA
TTATGGCTGAGTTAGCTTTGATTGGTGCTGATATACAAGAGGTTATTGGAAGTGCTATTGCTATT
AAGATCTTGAGTAACGGGGTTGTGCCTTTATGGGCTGGTGTTACTATTACTGCTTGTGATTGCTTC
ATCTTCCTATTTCTAGAGAACTACGGTGTGAGAAAATTGGAGGCTGTATTTGCGGTCCTCATTGG
AGTCATGGCAGTTACATTTGGATGGATGTTTGCTGATGCCAAACCCAGTGCCTCCGAACTTTTTC
TGGGTATCTTAATTCCAAAACTTAGCTCCAGAACAATACAGCAGGCTGTAGGAGTTGTGGGTTGC
ATTATCATGCCTCACAATGTGTTCCTGCATTCTGCTCTTGTACAGTCAAGGGAGATCGACCACAA
TAATAAAGTCCAGGTTCAAGAAGCTCTCAGATACTACTCCATAGAGTCAACCACTGCCCTTGTGA
TATCGTTCGTAATCAATTTGTTCGTGACGACTGTGTTTGCTAAAGGTTTCTATGGGACAGAACTG
GCCAATAGTATTGGCCTTGTAAATGCAGGGCAATATCTTCAAGACAAATACGGGGGTGGATTTTT
CCCAATCTTATACATCTGGGGTATTGGATTATTAGCTGCTGGCCAAAGTAGCACCATTACTGGGA

CCTATGCAGGACAGTTTATCATGGGAGGTTTCCTGAACATGAGGTTAAAGAAATGGCTAAGGGC
ATTGATCACTCGAAGTTGTGCTATCATCCCAACGATAATTGTTGCGCTTATTTTTGATACCTCTGA
AGATTCTCTAGATGTTCTGAATGAATGGCTAAATGTGCTGCAGTCAATACAGATTCCTTTTGCAC
TCATCCCTCTTCTCTGTTTGGTCTCCAAGGAGCGAATCATGGGCACTTTCAAAATTGGCTCCATCC
TTAAGGTCGTTTCTTGGCTTGTGGCGGCCCTGGTGATAGTAATCAATGGTTACCTTTTGCTCGACT
TTTTCTTCAATGAAGTGATTGGAGTAGCGTTTACCACTGCAGTATGCACTTTCACAGCTGCATAT
GCTGCGTTTATAATTTACCTCACTTCCAGGGGCATTACATGTTCTTCCTGGCGCGGCCCACCTAA
ACAGATAGAAGCAGAG

>S. dasyclados_NRAMP3_CDS

ATGGCAGTTACATTTGGATGGATGTTTGCTGATGCCAAACCCAGTGCCTCCGAACTTTTTCTGGG
TATCTTAATTCCAAAACTTAGCTCCAGAACAATACAACAGGCTGTTGGGGTTGTGGGTTGCATTA
TCATGCCTCACAATGTGTTCCTGCATTCTGCTCTTGTACAGTCAAGGGAGATCGACCACAATAAT
AAAGTCCAGGTTCAAGAAGCTGTCAGATACTACTCCATAGAGTCAACCACTGCCCTTGTGATATC
GTTCGTAATCAATTTGTTCGTGACGACTGTGTTTGCTCAAGGTTTCTATGGGACAGAACTGGCCA
ATAGTATTGGCCTTGTAAATGCAGGGCAATATCTTCAAGACAAATACGGGGGTGGATTTTTCCCA
ATCTTATACATCTGGGGTATTGGATTATTAGCTGCTGGCCAAAGTAGCACCATTACTGGGACCTA
TGCAGGACAGTTTATCATGGGAGGTTTCCTGAACATGAGGTTAAAGAAATGGCTAAGGGCATTG
ATCACTCGAAGTTGTGCTATCATCCCAACGATAATTGTTGCGCTTATTTTTGATACCTCTGAAGAT
TCTCTAGATGTTCTGAATGAATGGCTAAATGTGCTGCAGTCAATACAGATTCCTTTTGCACTCAT
CCCTCTTCTCTGTTTGGTCTCCAAGGAGCGAATCATGGGCACTTTCAAAATTGGCTCCATCCTTAA
GGTCGTTTCTTGGCTTGTGGCGGCCCTGGTGATAGTAATCAATGGCTACCTTTTGCTCGACTTTTT
CTTCAATGAAGTGACCGGAGTAGCGTTTACCACCGCAGTATGCACTTTTACAGCTGCATATGCTG
CGTTTATAATTTATCTCACTTCTAGGGGCATTACATGCTCTTCCTGGCGCGGCCCACCTAAACAG
ATAGAAGTAGAG

>S._viminalis_NRAMP3_CDS

ATGTCTTTAGATGAAAACCAGCAACCTTTATTGCAAGAAGAAGAAGAGAGAGCTTATGAT
TCTGATGAGAAAGTGCTCGTAATTGGGATTGATTCTGACGCCGAAAGCAGTGGCACGGTG
TTGCCACCGTTTTCATGGAAAAAGTTATGGTTGTTTACTGGTCCTGGGTTTTTAATGTCC
ATTGCGTTTTTGGATCCTGGAAATTTGGAAGGGGATCTTCAGGCTGGTGCAATTGCAGGC
TACTCTTTGCTTTGGCTCCTTTTATGGGCTACTGCTATGGGGTTGTTGGTGCAGTTGCTG
TCAGCCAGGCTTGGAGTGGCTACAGGGAGGCATTTAGCTGAGCTGTGTAGAGAAGAGTAT
CCAACCTGGGCTAGAATGATTTTGTGGATTATGGCTGAGTTAGCTTTGATTGGTGCTGAT
ATACAAGAGGTTATTGGAAGTGCTATTGCTATTAAGATCTTGAGTAACGGGGTTGTGCCT
TTATGGGCTGGTGTTACTATTACTGCTTGTGATTGCTTCATCTTCCTATTTCTAGAGAAC
TACGGTGTGAGAAAATTGGAGGCTGTATTTGCGGTCCTAATTGGAGTAATGGCAGTTACA
TTTGGATGGATGTTTGCTGATGCCAAACCCAGTGCCTCCGAACTTTTTCTGGGTATCTTA
ATTCCAAAACTTAGCTCCAGAACAATACAGCAGGCTGTTGGGGTTGTGGGTTGCATTATC
ATGCCTCACAATGTGTTCCTGCATTCTGCTCTTGTACAGTCAAGGGAGATCGACCACAAT
AATAAAGTCCAGGTTCAAGAAGCTGTCAGATACTACTCCATAGAGTCAACCACTGCCCTT
GTGATATCGTTCGTAATCAATTTGTTCGTGACGACTGTGTTTGCTCAAGGTTTCTATGGG
ACAGAACTGGCCAATAGTATTGGCCTTGTAAATGCAGGGCAATATCTTCAAGACAAATAC
GGGGGTGGATTTTTCCCAATCTTATACATCTGGGGTATTGGATTATTAGCTGCTGGCCAA
AGTAGCACCATTACTGGGACCTATGCAGGACAGTTTATCATGGGAGGTTTCCTGAACATG
AGGTTAAAGAAATGGCTAAGGGCATTGATCACTCGAAGTTGTGCTATCATCCCAACGATA
ATTGTTGCGCTTATTTTTGATACCTCTGAAGATTCTCTAGATGTTCTGAATGAATGGCTA
AATGTGCTGCAGTCAATACAGATTCCTTTTGCACTCATCCCTCTTCTCTGTTTGGTCTCC

AAGGAGCGAATCATGGGCACTTTCAAAATTGGCTCCATCCTTAAGGTCGTTTCTTGGCTT
GTGGCGGCCCTGGTGATAGTAATCAATGGTTACCTTTTGCTCGACTTTTTCTTCNNNNNNNNG

>S. fargesii_NRAMP3_CDS

ATGTCCATTGCGTTTTTGGATCCTGGAAATTTGGAAGGGGATCTTCAGGCTGGTGCGATT
GCAGGCTACTCTTTGCTTTGGCTCCTTTTATGGGCCACTGCTATGGGGTTGTTGGTGCAG
TTGCTGTCAGCCAGGCTTGGAGTGGCTACAGGGAGGCATTTAGCTGAGCTGTGTAGAGAA
GAGTATCCAACCTGGGCTAGAATGATTTTGTGGATTATGGCTGAGTTAGCTTTGATTGGT
GCTGATATACAAGAGGTTATTGGAAGTGCTATTGCTATTAAGATCTTGAGTAACGGGGTT
GTGCCTTTATGGGCTGGTGTTACTATTACTGCTTGTGATTGCTTCATCTTCCTATTTCTA
GAGAACTACGGTGTGAGAAAATTGGAGGCTGTATTTGCGGTCCTAATTGGAGTAATGGCA
GTTACATTTGGAATGATGTTTGCTGATGCCAAACCCAGTGCCTCCGAACTTTTTCTGGGT
ATCTTAATCCCAAAACTTAGCTCCAGAACAATACAACAGGCTGTAGGAGTTGTGGGTTGC
ATTATCATGCCTCACAATGTGTTCCTGCATTCTGCTCTTGTACAGTCAAGGGAGATCGAC
CACAATAATAAAGTCCAGGTTCAAGAAGCTCTCAGATACTACTCCATAGAGTCAACCACT
GCCCTTGTAATATCGTTCGTAATCAATTTGTTCGTGACGACTGTGTTTGCTCAAGGTTTC
TATGGGACAGAACTGGCCAATAGTATTGGCCTTGTAAATGCAGGGCAATATCTTCAAGAC
AAATACGGGGGTGGATTTTTCCCAATCTTATACATCTGGGGTATTGGATTATTAGCTGCT
GGCCAAAGTAGCACCATTACAGGGACCTATGCAGGACAGTTTATCATGGGAGGTTTCCTG
AACATGAGGTTAAAGAAATGGCTGAGGGCATTGATCACTCGAAGTTGTGCTATCATCCCA
ACGATAATTGTTGCGCTTGTTTTTGAAACCTCTGAAGAGTCTCTAGATGTTCTGAATGAA
TGGCTAAATGTGCTGCAGTCAATACAGATTCCTTTTGCACTCATCCCTCTTCTCTGTTTG
GTCTCCAAGGAGCGAATCATGGGCACTTTCAAAATTGGCTCCATTCTTAAGGTCGTTTCT
TGGCTTGTGGCGGCCCTGGTGATAATAATCAATGGTTACCTTTTGCTCGACTTTTTCTTC
AATGAAGTGACTGGAGTAGCGTATACCACTGCAGTATGCACTTTTACAGCTGCATATGCT
GCGTTTATAATTTATCTCACTTCTAGGGGCGTTACATGCTCTTCCTGGCGCGGCCCACCT
AAACAGAATCAACAGATAGAAGTAGAG

Supplementary data S2. Partial genomic sequence of *NRAMP3.1* and *RNAMP3.2* retrieved from *Populus mexicana* genome.

>P. mexicana_NRAMP3.1_genomic
nnnnnnnnnnnnnnnnnnnnnnnnnnnnnnnnnnnnnnnnnnnnnnnnnnnnnnnnnnnnnnnnnnnnnnnnnnnnnnnnnnnnnnnn
nnnnnnnnnnnnnnnnnnnnnnnnnnnnnnnnnnnnnnnnnnnnnnnnnnnnnnnnnnnnnnnnnnnnnnnnnnnnnnnnnnnnnnnn
nnnnnnnnnnnnnnnnnnnnnnnnnnnnnnnnnnnnnnnnnnnnnnnnnnnnnnnnnnnnnnnnnnnnnnnnnnnnnnnnnnnnnnnn
nnnnnnnnnnnnnnnnnnnnnnnnnnnnnnnnnnnnnnnnnnnnnnnnnnnnnnnnnnnnnnnnnnnnnnnnnnnnnnnnnnnnnnn
nnnnnnnnnnnnnnnnnnnnnnnnnnnnnnnnnnnnnnnnnnnnnnnnnnnnnnnnnnnnnnnnnnnnnnnnnnnnnnnnnnnnnnnn
nnnnnnnnnnnnnnnnnnnnnnnnnnnnnnnnnnnnnnnnnnnnnnnnnnnnnnnnnnnnnnnnnnnnnnnnnnnnnnnnnnnnnnnn
nnnnnnnnnnnnnnnnnnnnnnnnnnnnnnnnnnnnnnnnnnnnnnnnnnnnnnnnnnnnnnnnnnnnnnnnnnnnnnnnnnnnnnnn
nnnnnnnnnnnnnnnnnnnnnnnnnnnnnnnnnnnnnnnnnnnnnnnnnnnnnnnnnnnnnnnnnnnnnnnnnnnnnnnnnnnnnnnn
nnnnnnnnnnnnnnnnnnnnnnnnnnnnnnnnnnnnnnnnnnnnnnnnnnnnnnnnnnnnnnnnnnnnnnnnnnnnnnnnnnnnnnnn
nnnnnnnnnnnnnnnnnnnnnnnnnnnnnnnnnnnnnnnnnnnnnnnnnnnnnnnnnnnnnnnnnnnnnnnnnnnnnnnnnnnnnnnn
nnnnnnnnnnnnnnnnnnnnnnnnnnnnnnnnnnnnnnnnnnnnnnnnnnnnnnnnnnnnnnnnnnnnnnnnnnnnnnnnnnnnnnnn
nnnnnnnnnnnnnnnnnnnnnnnnnnnnnnnnnnnnnnnnnnnnnnnnnnnnnnnnnnnnnnnnnnnnnnnnnnnnnnnnnnnnnnnn
nnnnnnnnnnnnnnnnnnnnnnnnnnnnnnnnnnnnnnnnnnnnnnnnnnnnnnnnnnnnnnnnnnnnnnnnnnnnnnnnnnnnnnnn
nnnnnnnnnnnnnnnnnnnnnnnnnnnnnnnnnnnnnnnnnnnnnnnnnnnnnnnnnnnnnnnnnnnnnnnnnnnnnnnnnnnnnnnn
nnnnnnnnnnnnnnnnnnnnnnCTGGCCAAAAATTTCAATTTACAAGCTCCAGTTTCTCTTGTTTCTCTTCA
TTTGTCCGACTATAGTACTGCTTTGTATCTAGGAGACAGTTAATTGGATTCTCTATTACAGGCATC
TTAATTCCAAAACTTAGCTCCAAAACAATAAAACAGGCTGTTGGAGTTGTGGGTTGCATTATCAT
GCCTCACAATGTGTTCTTGCATTCTGCTCTTGTACAGTCAAGGGAGATTGACCACAATAAGAAAG

GCCAGGTTCAAGAAGCTCTCAGATACTACTCCATAGAGTCAACTGCTGCCCTTGCAATATCATTC
ATGATCAATTTGTTTGTGACGACTGTTTTTGCTAAAGGTTTCCATGGGACAAAACTGGCCAATAG
TATTGGCCTTGTAAATGCAGGGCAATATCTTCAAGATAAATACAGGGGTGGATTTTTCCCAATTT
TATACATCTGGGGTATTGGGTTATTAGCAGCTGGTCAAAGTAGCACCATTACTGGCACCTATGCA
GGGCAGTTTATCATGGGAGGTTTCCTGAACTTGGGATTAAAGAAATGGCAGAGGGCATTGATTA
CTCGAAGCTGTGCTATCATCCCAACTATAATTGTTGCACTTGTTTTTGATACTTCTGAAGACTCAC
TAGATGTTCTGAATGAATGGCTAAATATGCTTCAGTCTATTCAGATTCCTTTTGCACTCATCCCTC
TTCTTTGCTTGGTCTCCAAGGAGCAAATCATGGGCACCTTCACAGTTGGCCCCATTCTTAAGGTA
TGTTGACTAGTCTCTGTAACTCTGTTGTCCAATGTTTCTGTCATTAAACTCAAGATAACCCATTAA
ATTGGTGACAGTAGATTGGATCAAGGAAGCAGTTGCCATTTTCAATTTGTTTGGAAGTTTGTGTA
Annnnnnnnnnnnnnnnnnnnnnnnnnnnnnnnnnnnnnnnnnnnnnnnnnnnnnnnnnnnnnnnnnnnnnnnnnnnnnnnnnnnnnn
nnnnnnnnnnnnnnnnnnnnnnnnnnnnnnnnnnnnnnnnnnnnnnnnnnnnnnnnnnnnnnnnnnnnnnnnnnnnnnnnnnnnnnnn
nnnnnnnnnnnnnnnnnnnnnnnnnnnnnnnnnnnnnnnnnnnnnnnnnnnnnnnnnnnnnnnnnnnnnnnnnnnnnnnnnnnnnnnn
nnnnnnnnnnnnnnnnnnnnnnnnnnnnnnnnnnnnnnnnnnnnnnnnnnnnnnnnnnnnnnnnnnnnnnnnnnnnnnnnnnnnnnnn
nnnnnnnnnnnnnnnnnnnnnnnnnnnnnnnnnnnnnnnnnnnnnnnnnnnnnnnnnnnnnnnnnnnnnnnnnnnnnnnnnnnnnnnn
nnnnnnnnnnnnnnnnnnnnnnnnnnnnnnnnnnnnnnnnnnnnnnnnnnnnnnnnnnnnnnnnnnnnnnnnnnnnnnnnnnnnnnnn
nnnnnnnnnnnnnnnnnnnnnnnnnnnnnnnnnnnnnnnnnnnnnnnnnnnnnnnnnnnnnnnnnnnnnnnnnnnnnnnnnnnnnnnn
nnnnnnnnnnnnnnnnnnnnnnnnnnnnnnnnnnnnnnnnnnnnnnnnnnnnnnnnnnnnnnnnnnnnnnnnnnnnnnnnnnnnnnnn
nnnnnnnnnnnnnnnnnnnnnnnnnnnnnnnnnnnnnnnnnnnnnnnnnnnnnnnnnnnnnnnnnnnnnnnnnnnnnnnnnnnnnnnn
nnnnnnnnnnnnnnnnnnn

>P. mexicana_NRAMP3.2_genomic
nnnnnnnnnnnnnnnnnnnnnnnnnnnnnnnnnnnnnnnnnnnnnnnnnnnnnnnnnnnnnnnnnnnnnnnnnnnnnnnnnnnnnnnn
nnnnnnnnnnnnnnnnnnnnnnnnnnnnnnnnnnnnnnnnnnnnnnnnnnnnnnnnnnnnnnnnnnnnnnnnnnnnnnnnnnnnnnnn
nnnnnnnnnnnnnnnnnnnnnnnnnnnnnnnnnnnnnnnnnnnnnnnnnnnnnnnnnnnnnnnnnnnnnnnnnnnnnnnnnnnnnnnn
nnnnnnnnnnnnnnnnnnnnnnnnnnnnnnnnnnnnnnnnnnnnnnnnnnnnnnnnnnnnnnnnnnnnnnnnnnnnnnnnnnnnnnnn
nnnnnnnnnnnnnnnnnnnnnnnnnnnnnnnnnnnnnnnnnnnnnnnnnnnnnnnnnnnnnnnnnnnnnnnnnnnnnnnnnnnnnnnn
nnnnnnnnnnnnnnnnnnnnnnnnnnnnnnnnnnnnnnnnnnnnnnnnnnnnnnnnnnnnnnnnnnnnnnnnnnnnnnnnnnnnnnnn
nnnnnnnnnnnnnnnnnnnnnnnnnnnnnnnnnnnnnnnnnnnnnnnnnnnnnnnnnnnnnnnnnnnnnnnnnnnnnnnnnnnnnnnn
nnnnnnnnnnnnnnnnnnnnnnnnnnnnnnnnnnnnnnnnnnnnnnnnnnnnnnnnnnnnnnnnnnnnnnnnnnnnnnnnnnnnnnnn
nnnnnnnnnnnnnnnnnnnnnnnnnnnnnnnnnnnnnnnnnnnnnnnnnnnnnnnnnnnnnnnnnnnnnnnnnnnnnnnnnnnnnnnn
nnnnnnnnnnnnnnnnnnnnnnnnnnnnnnnnnnnnnnnnnnnnnnnnnnnnnnnnnnnnnnnnnnnnnnnnnnnnnnnnnnnnnnnn
nnnnnnnnnnnnnnnnnnnnnnnnnnnnnnnnnnnnnnnnnnnnnnnnnnnnnnnnnnnnnnnnnnnnnnnnnnnnnnnnnnnnnnnn
nnnnnnnnnnnnnnnnnnnnnnnnnnnnnnnnnnnnnnnnnnnnnnnnnnnnnnnnnnnnnnnnnnnnnnnnnnnnnnnnnnnnnnnn
nnnnnnnnnnnnnnnnnnnnnnnnnnnnnnnnnnnnnnnnnnnnnnnnnnnnnnnnnnnnnnnnnnnnnnnnnnnnnnnnnnnnnnnn
nnnnnnnnnnnnnnnnnnnnnnnnnnnnnnnnnnnnnnnnnnnnnnnnnnnnnnnnnnnnnnnnnnnnnnnnnnnnnnnnnnnnnnnn
nnnnnnnnnnnnnnnnnnnnnnnnnnnnnnnnnnnnnnnnnnnnnnnnnnnnnnnnnnnnnnnnnnnnnnnnnnnnnnnnnnnnnnnn
nnnnnnnnnnnnnnnnnnnnnnnnnnnnnnnnnnnnnnnnnnnnnnnnnnnnnnnnnnnnnnnnnnnnnnnnnnnnnnnnnnnnnnnn
nnnnnnnnnnnnnnnnnnnnnnnnnnnnnnnnnnnnnnnnnnnnnnnnnnnnnnnnnnnnnnnnnnnnnnnnnnnnnnnnnnnnnnnn
nnnnnnnnnnnnnnnnnnnnnnnnnnnnnnnnnnnnnnnnnnnnnnnnnnnnnnnnnnnnnnnnnnnnnnnnnnnnnnnnnnnnnnnn
nnnnnnnnnnnAATTTGTTTGTGACTACTGTTTTTGCTAAAGGTTTCTATGGGACAGAACTGGCCAAT
AGTATTGGCCTTGTAAATGCAGGGCAATATCTTCAAGACAAATATGGGGGTGGATTTTTCCCAAT
TTTATACATCTGGGGTATTGGATTACTAGCAGCTGGCCAAAGCAGCACCATTACCGGAACTTATG
CAGGACAGTTTATCATGGGAGGTTTCCTGAACTTGAGGTTAAAGAAATGGCTGAGGGCATTGAT
CACTCGAAGCTGTGCTATCATCCCAACTATGATTGTTGCACTTGTTTTTGATACCTCCGAAGACTC
ACTAGATGTTCTGAATGAATGGCTAAATGTGCTGCAGTCAATACAGATTCCTTTTGCACTCATCC
CTCTTCTCTGCTTGGTATCCAAGGAGCAAATCATGGGCACTTTCAAAATCAACCCCATTCTTAAG
GTATGTTGACTAGTCTGTTATTCTGCGGTATTTGTCTTGAAGCTTAAGATGGCCCTTTAAATTGGT
GACAGTTGACTAAATCAAGGAACCAATTGCCTTTTTCAATTnTTTGGAATCTCCATATAACCAAA
TCTCTCAACTTGTGTCGTTCTTCAATACTCCAATGCATCATAAAGTCAAAAACATGTGACATTTTG
TTAATGTGTAAACATGTCTGAAATTATGCATGTGATGGATGAATCTTGGGTAAAAAATTGnnnnnnn
nnnnnnnnnnnnnnnnnnnnnnnnnnnnnnnnnnnnnnnnnnnnnnnnnnnnnnnnnnnnnnnnnnnnnnnnnnnnnnnnnnnnnnn

nnnnnnnnnnnnnnnnnnnnnnnnnnnnnnnnnnnnnnnnnnnnnnnnnnnnnnnnnnnnnnnnnnnnnnnnnnnnnnnnnnnnnnnnn
nnnnnnnnnnnnnnnnnnnnnnnnnnnnnnnnnnnnnnnnnnnnnnnnnnnnnnnnnnnnnnnnnnnnnnnnnnnnnnnnnnnnnnnn
nnnnnnnnnnnnnnnnnnnnnnnnnnnnnnnnnnnnnnnnnnnnnnnnnnnnnnnnnnnnnnnnnnnnnnnnnnnnnnnnnnnnnnnn
nnnnnnnnnnnnnnnnnnnnnnnnnnnnnnnnnnnnnnnnnnnnnnnnnnnnnnnnnnnnnnnnnnnnnnnnnnnnnnnnnnnnnnnnn
nnnnnnnnnnnnnnnnnnnnnnnnnnnnnnnnnnnnnnnnnnnnnnnnnnnnnnnnnnnnnnnnnnnnnnnnnnnnnnnnnnnnnnnn
nnnnnnnnnnnnnnnnnnnnnnnnnnnnnnnnnnnnnnnnnnnnnnnnnnnnnnnnnnnnnnnnnnnnnnnnnnnnnnnnnnnnnnnn
nnnnnnnnnnnnnnnnnnnnnnnnnnnnnnnnnnnnnnnnnnnnnnnnnnnnnnnnnnnnnnnnnnnnnnnnnnnnnnnnnnnnnnnnn
nnnnn

Supplementary data S3. Protein sequences used to construct the phylogenetic tree shown in figure 2. $ at the end of the name indicate incomplete protein sequences.

>P. trichocarpa_NRAMP3.1
MPSPEEDPQPLLKDQEETAYDSDGKVLSFGIDYDTESGGSTVVPSFSWRKLWLFTGPGFLMCIAFLDP
GNLEGDLQAGAIAGYSLLWLLLWATAMGLLVQLLSARLGVATGRHLAELCREEYPTWARMILWIM
AELALIGADIQEVIGSAIAIQILSNGVLPLWAGVIITASDCFIFLFLENYGVRKLEAAFGILIGIMAVTFA
WMFADAKPSAPELFLGILIPKLSSKTIKQAVGVVGCIIMPHNVFLHSALVQSREIDHNKKGQVQEALR
YYSIESTAALAISFMINLFVTTIFAKGFHGTELANSIGLVNAGQYLQDKYGGGFFPILYIWGIGLLAAG
QSSTITGTYAGQFIMGGFLNLGLKKWLRALITRSCAIIPTIIVALVFDTSEDSLDVLNEWLNMLQSIQIP
FALIPLLCLVSKEQIMGTFTVGPILKMVSWLVAALVMLINGYLLLDFFSNEVTGVVFTTVVCAFTGAY
VTFIIYLISREVTISTWYCPT*

>P. trichocarpa_NRAMP3.2
MPVEENYQPLLQEEEERAYDSDEKVLIIGVDSDTESGGSTVLPPFSWKKLWLFTGPGFLMSIAFLDPG
NLEGDLQAGAIAGYSLLWLLLWATAMGLLVQLLSARLGVATGRHLAELCREEYPTWASMVLWIMA
ELALIGADIQEVIGSAIAIKILSNGFVPLWAGVTITACDCFIFLFLENYGVRKLEAVFAVLIGIMAVTFG
WMFADAKPSASELFLGILIPKLSSRTIQQAVGVVGCIIMPHNVFLHSALVQSREIDHNKKDRVQEALR
YYSIESTTALVISFVINLFVTTVFAKGFYGTELANSIGLVNAGQYLQDKYGGGFFPILYIWGIGLLAAG
QSSTITGTYAGQFIMGGFLNLRLKKWLRALITRSCAIIPTMIVALVFDTSEDSLDVLNEWLNVLQSIQIP
FALIPLLCLVSKEQIMGTFKIGPILKMVAWLVAALVMVINGYLLLDFFFNEVTGVAFTTVVCGFTGAY
VAFIIYLISRGFTCFSRCCPSKQIEV*

>P. tremula_NRAMP3.1
MPLPEEDPQPLLKDQEETAHDSDGKVLSFGIDYDTESGGSTVVPSFSWRKLWLFTGPGFLMCIAFLDP
GNLEGDLQAGAIAGYSLLWLLLWATAMGLLVQLLSARLGVATGRHLAELCREEYPTWARMILWIM
AELALIGADIQEVIGSAIAIQILSNGVLPLWAGVIITASDCFIFLFLENYGVRKLEAAFGILIGIMAVAFA
WMFADAKPSAPELFLGILIPKLSSKTIKQAVGVVGCIIMPHNVFLHSALVQSREIDCNKKGQVQEALR
YYSIESTAALAISFMINLFVTTIFAKGFHGTELANSIGLVNAGQYLQDKYGGGFFPILYIWGIGLLAAG
QSSTITGTYAGQFIMGGFLNLGLKKWLRALITRSCAIIPTIIVALVFDTSEDSLDVLNEWLNMLQSIQIP
FALIPLLCLVSKEQIMGTFTVGPILKMVSWLVAALVMLINGYLLLDFFSNEVTGVVFTTVVCAFTGAY
VTFIIYLISREVSISTWYCPT*

>P. tremula_NRAMP3.2
MSVEENQQPLLQEEEERAYDSDEKVLIIGVDSDTESGGSTVLPPFSWKKLWLFTGPGFLMSIAFLDPG
NLEGDLQAGAIAGYSLLWLLFWATAMGLLVQLLSARLGVATGRHLAELCREEYPTWASMVLWIMA
ELALIGADIQEVIGSAIALKILSNGFLPLWAGVTITACDCFIFLFLENYGVRKLEAVFAVLIGIMAVTFG
WMFADAKPSASELFLGILIPKLSSRTIQQAVGVVGCIIMPHNVFLHSALVQSREIDHNKKGRVQEALR
YYSIESTTALVISFVINLFVTTVFAKGFYGTELANSIGLVNAGQYLQDKYGGGFFPILYIWGIGLLAAG
QSSTITGTYAGQFIMGGFLNLRLKKWLRALITRSCAIIPTMIVALVFDASEDSLDVLNEWLNVLQSIQIP
FALIPLLFLVSKEQIMGTFKIGPILKMVSWLVAALVIVINGYLLLDFFVNEVTGVAFTTVVCGFTGAYV
AFIIYLISRGFKCFSWCCQSKQIEVE*

>P. tremuloides_NRAMP3.1

MPLPEEDPQPLLKDQEETAYDSDGKVLSFGIDYDTESGGSTVLPPFSWKKLWLFTGPGFLMSIAFLDP
GNLEGDLQAGAIAGYSLLWLLLWATAMGLLVQLLSARLGVATGRHLAELCREEYPTWARMILWIM
AELALIGADIQEVIGSAIAIQILSNGVLPLWAGVIITASDCFIFLFLENYGVRKLEAAFGILIGIMAVTFA
WMFADAKPSAPELFLGILIPKLSSKTIKQAVGVVGCIIMPHNVFLHSALVQSREIDHNKKGQVQEALR
YYSIESTAALAISFMINLFVTTIFAKGFHGTELANSIGLVNAGQYLQDKYGGGFFPILYIWGIGLLAAG
QSSTITGTYAGQFIMGGFLNLGLKKWLRALITRSCAIIPTIIVALVFDTSEDSLDVLNEWLNMLQSIQIP
FALIPLLCLVSKEQLMGTFTVGPILKMVSWLVAALVMLINGYLLLDFFSNEVTGVLFTTVVCAFTGA
YVTFIIYLISREVSISTWYCPT*

>P. tremuloides_NRAMP3.2
MPVEENQQPLLQEEEERAYDSDEKVLIIGVDSDTESGGSTVLPPFSWKKLWLFTGPGFLMSIAFLDPG
NLEGDLQAGAIAGYSLLWLLLWATAMGLLVQLLSARLGVATGRHLAELCREEYPTWASMVLWIMA
ELALIGADIQEVIGSAIALKILSNGFLPLWAGVTITACDCFIFLFLENYGVRKLEAVFAVLIGLMAVTFG
WMFADAKPSASELFLGILIPKLSSRTIQQAVGVVGCIIMPHNVFLHSALVQSREIDHNKKGRVQEALR
YYSIESTTALVISFVINLFVTTVFAKGFYGTELANSIGLVNAGQYLQDKYGGGFFPILYIWGIGLLAAG
QSSTITGTYAGQFIMGGFLNLRLKKWLRALITRSCAIIPTMIVALVFDASEDSLDVLNEWLNVLQSIQIP
FALIPLLCLVSKEQIMGTFKIGPILKMVSWLVAALVIVINGYLLLDFFVNEVAGVAFTTVVCGFTGAY
VAFIIYLISRGFTCFSWCCPSKQIEVE*

>P. grandidentata_NRAMP3.1
MPLPEEDPQPLLKDQEETAYDSDGKVLSFGIDYDTESGGSTVVPSFSWRKLWLFTGPGFLMCIAFLDP
GNLEGDLQAGAIAGYSLLWLLLWATAMGLLVQLLSARLGVATGRHLAELCREEYPTWARMILWIM
AELALIGADIQEVIGSAIAIQILSNGVLPLWAGVIITASDCFIFLFLENYGVRKLEAAFGILIGIMAVTFA
WMFADAKPSAPELFLGILIPKLSSKTIKQAVGVVGCIIMPHNVFLHSALVQSREIDHNKKGQVQEALR
YYSIESTAALAISFMINLFVTTIFAKGFHGTELANSIGLVNAGQYLQDKYGGGFFPILYIWGIGLLAAG
QSSTITGTYAGQFIMGGFLNLGLKKWLRALITRSCAIIPTIIVALVFDTSEDSLDVLNEWLNMLQSIQIP
FALIPLLCLVSKEQLMGTFTVGPILKMVSWLVAALVMLINGYLLLDFFSNEVTGVVFTTVVCAFTGA
YVTFIIYLISREVSISTWYCPT*

>P. grandidentata_NRAMP3.2
MPVEENQQPLLQEEEERAYDSDEKVLIIGVDSDTESGGSTVLPPFSWKKLWLFTGPGFLMSIAFLDPG
NLEGDLQAGAIAGYSLLWLLLWATAMGLLVQLLSARLGVATGRHLAELCREEYPTWASMILWIMA
ELALIGADIQEVIGSAIALKILSNGFLPLWAGVTITACDCFIFLFLENYGVRKLEAVFAVLIGIMAVTFG
WMFADAKPSASELFLGILIPKLSSRTIQQAVGVVGCIIMPHNVFLHSALVQSREIDHNKKGRVQEALR
YYSIESTTALVISFVINLFVTTVFAKGFYGTELANSIGLVNAGQYLQDKYGGGFFPILYIWGIGLLAAG
QSSTITGTYAGQFIMGGFLNLRLKKWLRALITRSCAIIPTMIVALVFDASEDSLDVLNEWLNVLQSIQIP
FALIPLLCLVSKEQIMGTFKIGPILKMVSWLVAALVIVINGYLLLDFFVNEVAGVAFTTVVCGFTGAY
VAFIIYLISRGFTCFSRCCPSKQIEVE*

>P. alba_NRAMP3.1
MPLPEEDPQPLLKDQEETAYDSDGKVLSFGIDYDTESGGSTVVPSFSWRKLWLFTGPGFLMCIAFLDP
GNLEGDLQAGAIAGYSLLWLLLWATAMGLLVQLLSARLGVATGRHLAELCREEYPTWARMILWIM
AELALIGADIQEVIGSAIAIQILSNGVLPLWAGVIITASDCFIFLFLENYGVRKLEAAFGILIGIMAVTFA
WMFADAKPSAPELFLGILIPKLSSKTIKQAVGVVGCIIMPHNVFLHSALVQSREIDHNKKGQVQEALR
YYSIESTAALAISFMINLFVTTVFAKGFHGTELANSIGLVNAGQYLQDKYGGGFFPILYIWGIGLLAAG
QSSTITGTYAGQFIMGGFLNLGLKKWLRALITRSCAIIPTIIVALVFDTSEDSLDVLNEWLNMLQSIQIP
FALIPLLCLVSKEQIMGTFTVGPILQMVSWLVAALVMLINGYLLLDFFSNEVTGVVFTTVVCAFTGAY
VTFIIYLISREVTISTWYCPT*

>P. alba_NRAMP3.2
MPVEENQQPLLQEEEERAYDSDEKVLIIGVDSDTESGGSTVLPPFSWKKLWLFTGPGFLMSIAFLDPG
NLEGDLQAGAIAGYSLLWLLLWATAMGLLVQLLSARLGVATGRHLAELCREEYPTWASMVLWIMA
ELALIGADIQEVIGSAIALKILSNGFLPLWAGVTITACDCFIFLFLENYGVRKLEAVFAVLIGIMAVTFG
WMFADAKPSASELFLGILIPKLSSRTIQQAVGVVGCIIMPHNVFLHSALVQSREIDHNKKGRVQEALR

YYSIESTTALVISFVINLFVTTVFAKGFYGTELANSIGLVNAGQYLQDKYGGGFFPILYIWGIGLLAAG
QSSTITGTYAGQFIMGGFLNLRLKKWLRALITRSCAIIPTMIVALVFDTSEDSLDVLNEWLNVLQSIQIP
FALIPLLCLVSKEQIMGTFKIGPILKVSWLVAALVIVINGYLLLDFFVNEVAGVAFTTVLCGFTGAYVA
FIIYLISRGFTCFFWCCQSKQIEVE*

>P. cathayana_NRAMP3.1
MPLPEEDPQPLLKDQEETAYDSDGKVLSFGIDYDTESGGSTVVPSFSWRKLWLFTGPGFLMCIAFLDP
GNLEGDLQAGAIAGYSLLWLLLWATAMGLLVQLLSARLGVATGRHLAELCREEYPTWARMILWIM
AELALIGADIQEVIGSAIAIQILSNGVLPLWAGVIITASDCFIFLFLENYGVRKLEAAFGILIGIMAVTFA
WMFADAKPSAPELFLGILIPKLSSKTIKQAVGVVGCIIMPHNVFLHSALVQSREIDHNKKGQVQEALR
YYSIESTAALAISFMINLFVTTVFAKGFHGTELANSIGLVNAGQYLQDKYGGGFFPILYIWGIGLLAAG
QSSTITGTYAGQFIMGGFLNLGLKKWLRALITRSCAIIPTIIVALVFDTSEDSLDVLNEWLNMLQSIQIP
FALIPLLCLVSKEQIMGTFTVGPILQMVSWLVAALVMLINGYLLLDFFSNEVTGVVFTTVVCAFTGAY
VTFIIYLISREVTISTWYCPT*

>P. cathayana_NRAMP3.2
MPVEENHQPLLQEEEERAYDSDEKVLIIGVDSDTESGGSTVLPPFSWKKLWLFTGPGFLMSIAFLDPG
NLEGDLQAGAIAGYSLLWLLLWATAMGLLVQLLSARLGVATGRHLAELCREEYPTWASMVLWIMA
ELALIGADIQEVIGSAIAIKILSNGFVPLWAGVTITACDCFIFLFLENYGVRKLEAVFAVLIGIMAVTFG
WMFADAKPSASELFLGILIPKLSSRTIQQAVGVVGCIIMPHNVFLHSALVQSREIDHNKKDRVQEALR
YYSIESTTALVISFVINLFVTTVFAKGFYGTELANSIGLVNAGQYLQDKYGGGFFPILYIWGIGLLAAG
QSSTITGTYAGQFIMGGFLNLRLKKWLRALITRSCAIIPTMIVALVFDTSEDSLDVLNEWLNVLQSIQIP
FALIPLLCLVSKEQIMGTFKIGPILKMVAWLVAALVMVINGYLLLDFFFNEVTGVAFTTVVCGFTGAY
AAFIIYLISRGFTCFSRCCPSKQIEVE*

>P. simonii_NRAMP3.1
MPLPEEDPRPLLKDQEETAYDSDGKVLSFGIDYDTESGGSTVVPSFSWRKLWLFTGPGFLMCIAFLDP
GNLEGDLQAGAIAGYSLLWLLLWATAMGLLVQLLSARLGVATGRHLAELCREEYPTWARMILWIM
AELALIGADIQEVIGSAIAIQILSNGVLPLWAGVIITASDCFIFLFLENYGVRKLEAAFGILIGIMAVTFA
WMFADAKPSAPELFLGILIPKLSSKTIKQAVGVVGCIIMPHNVFLHSALVQSREIDHNKKGQVQEALR
YYSIESTAALAISFMINLFVTTVFAKGFHGTELANSIGLVNAGQYLQDKYGGGFFPILYIWGIGLLAAG
QSSTITGTYAGQFIMGGFLNLGLKKWLRALITRSCAIIPTTIVALVFDTSEDSLDVLNEWLNMLQSIQIP
FALIPLLCLVSKEQIMGTFTVGPILQMVSWLVAALVMLINGYLLLDFFSNEVTGVVFTTVVCAFTGAY
VTFIIYLISREVTISTWYCPT*

>P. simonii_NRAMP3.2
MPVEENHQPLLQEEEERAYDSDEKVLIIGVDSDTESGGSTVLPPFSWKKLWLFTGPGFLMSIAFLDPG
NLEGDLQAGAIAGYSLLWLLLWATAMGLLVQLLSARLGVATGRHLAELCREEYPTWASMVLWIMT
ELALIGADIQEVIGSAIAIKILSNGFVPLWAGVTITACDFIFLFLENYGVRKLEAVFAVLIGIMAVTFGW
MFADAKPSASELFLGILIPKLSSRTIQQAVGVVGCIIMPHNVFLHSALVQSREIDHNKKDRVQEALRY
YSIESTTALVISFVINLFVTTVFAKGFYGTELANSIGLVNAGQYLQDKYGGGFFPILYIWGIGLLAAGQ
SSTITGTYAGQFIMGGFLNLRLKKWLRALITRSCAIIPTMIVALVFDTSEDSLDVLNEWLNVLQSIQIPF
ALIPLLCLVSKEQIMGTFKIGPILKMVSWLVAALVMVINGYLLLDFFFNEVTGVAFTTVVCGFTGAYV
AFIIYLISRGFTCFSRCCPSKQIEVE*

>P. lasiocarpa_NRAMP3.1
MPLPEEDPKPLLKDQEETAYDSDGKVLSFGIDYGTESGGSTVVPSFSWRKLWLFTGPGFLMCIAFLDP
GNLEGDLQAGAIAGYSLLWLLLWATAMGLLVQLLSARLGVATGRHLAELCREEYPTWARMILWIM
AELALIGADIQEVIGSAIAIQILSNGVLPLWAGVIITASDCFIFLFLENYGVRKLEAAFGILIGIMAVTFA
WMFADAKPSAPELFLGILIPKLSSKTIKQAVGVVGCIIMPHNVFLHSALVQSREIDHNKKGQVQEALR
YYSIESTAALAISFMINLFVTTVFAKGFHGTELANSIGLVNAGQYLQDKYGGGFFPIFYIWGIGLLAAG
QSSTITGTYAGQFIMGGFLNLGLKKWLRALITRSCAIIPTIIVALVFDTSEDSLDVLNEWLNMLQSIQIP
FALIPLLCLVSKEQIMGTFTVGPILQMVSWLVAALVMLINGYLLLDFFSNEVTGVVFTTVVCAFTGAY
VTFIIYLISREVTISTWYCPT*

>P. lasiocarpa_NRAMP3.2
MPVEENHQPLLQEEEERAYDSDEKVLIIGVDSDTESGGSTVLPPFSWKKLWLFTGPGFLMSIAFLDPG
NLEGDLQAGAIAGYSLLWLLLWATAMGLLVQLLSARLGVATGRHLAELCREEYPTWASMVLWIMA
ELALIGADIQEVIGSAIAIKILSNGFVPLWAGVTITACDWFIFLFLENYGVRKLEAVFAVLIGIMAVTFG
WMFADAKPSASELFLGILIPKLSSRTIQQAVGVVGCIIMPHNVFLHSALVQSREIDHNKKDRVQEALR
YYSIESTTALVISFVINLFVTTVFAKGFYGTELANSIGLVNAGQYLQDKYGGGFFPILYIWGIGLLAAG
QSSTITGTYAGQFIMGGFLNLRLKKWLRALITRSCAIIPTMIVALVFDTSEDSLDVLNEWLNVLQSIQIP
FALIPLLCLVSKEQIMGTFKIDPILKMVSWLVAALVMVINGYLLLDFFFNEVTGVAFTTVVCGFTGAY
VAFIIYLISRGFTCFSRCCPSKQIEVE*

>P. maximowiczii_NRAMP3.1
MPLPEEDPQPLLKDQEETAYDSDGKVLSFGIDYDTESGGSTVVPSFSWRKLWLFTGPGFLMCIAFLDP
GNLEGDLQAGAIAGYSLLWLLLWATAMGLLVQLLSARLGVATGRHLAELCREEYPTWARMILWIM
AELALIGADIQEVIGSAIAIQILSNGVLPLWAGVIITASDCFIFLFLENYGVRKLEAAFGILIGIMAVTFA
WMFADAKPSAPELFLGILIPKLSSKTIKQAVGVVGCIIMPHNVFLHSALVQSREIDHNKKGQVQEALR
YYSIESTAALAISFMINLFVTTVFAKGFHGTELANSIGLVNAGQYLQDKYGGGFFPILYIWGIGLLAAG
QSSTITGTYAGQFIMGGFLNLGLKKWLRALITRSCAIIPTIIVALVFDTSEDSLDVLNEWLNMLQSIQIP
FALIPLLCLVSKEQIMGTFTVGPILKMVSWLVAALVMLINGYLLLDFFSNEVTGVVFTTVVCAFTGAY
VTFIIYLISREVTISTWYCPT*

>P. maximowiczi_NRAMP3.2
MPVEENHQPLLQEEEERAYDSDEKVLIIGVDSDTESGGSTVLPPFSWKKLWLFTGPGFLMSIAFLDPG
NLEGDLQAGAIAGYSLLWLLLWATAMGLLVQLLSARLGVATGRHLAELCREEYPTWASMVLWIMA
ELALIGADIQEVIGSAIAIKILSNGFVPLWAGVTITACDCFIFLFLENYGVRKLEAVFAVLIGIMAVTFG
WMFADAKPSASELFLGILIPKLSSRTIQQAVGVVGCIIMPHNVFLHSALVQSREIDHNKKDRVQEALR
YYSIESTTALVISFVINLFVTTVFAKGFYGTELANSIGLVNAGQYLQDKYGGGFFPILYIWGIGLLAAG
QSSTITGTYAGQFIMGGFLNLRLKKWLRALITRSCAIIPTMIVALVFDTSEDSLDVLNEWLNVLQSIQIP
FALIPLLCLVSKEQIMGTFKIGPILKMVAWLVAALVMVINGYLLLDFFFNEVTGVAFTTVVCGFTGAY
AAFIIYLISRGFTCFSRCCPSKQIEVE*

>P. euphratica_NRAMP3.1
MPLPEEDPQPLLEDQEETAYDSNGKVLSFGIDYDTESGGSTVVPSFSWRKLWLFTGPGFLMCIAFLDP
GNLEGDLQAGAIAGYSLLWLLLWATAMGLLVQLLSARLGVATGRHLAELWREE*PTWARMILWIM
AELALIGADIQEVIGSAIAIQILSNGVLPLWAGVIITASDSFIFLFLENYGVRKLEAAFGILIGIMAVTFAC
MFADAKPSAPELFLGILIPKLSSKTIKQAVGVVGCIIMPHNVFLHSALVQSREIDHNKKGRVQEALRY
YSIESTAALAISFMNNLFVTTVFAKGFHRTELANSIGLVNAGQYLQDKYGGGFFPILYIWGIGLLAAG
QSSTITGTYAGQFIMGGFLNLGLKKWLRALITRSCAIVPTIIVALVFDTSEDSLDVLNEWLNMLQSIQIP
FALIPLLCLVSKEQIMGTFTVGPILKMVSWFVAALVMLINGYLLLDFFSNEVTGVVFITVVCAFTGAY
VTFTIYLISREVTISTWYCPS*

>P. euphratica_NRAMP3.2
MPVEENHQPLLQEEEERAYDSDEKVLIIGVDSDTESGGSTVLPPFSWKKLWLFTGPGFLMSIAFLDPG
NLEGDLQAGAIAGYSLLWLLLWATAMGLLVQLLSARLGVATGRHLAELCREEYPTWASMVLWIMA
ELALIGADIQEVIGSAIAIKILSNGFVPLWAGVTITACDCFIFLFLENYGVRKLEAVFAVLIGIMAVTFG
WMFADAKPSASELFLGILIPKLSSRTIQQAVGVVGCIIMPHNVFLHSALVQSREIDHNKKDRVQEALR
YYSIESTTALVISFVINLFVTTVFAKGFYGTELANSIGLVNAGQYLQDKYGGGFFPILYIWGIGLLAAG
QSSTITGTYAGQFIMGGFLNLRLKKWLRALITRSCAIIPTMIVALVFDTSEDSLDVLNEWLNVLQSIQIP
FALIPLLCLVSKEQIMGTFKIGPILKMVAWLVAALVMVINGYLLLDFFFNEVTGVAFTTVVCGFTGAY
AAFIIYLISRGFTCFSRCCPSKQIEVE*

>P. ussuriensis_NRAMP3.1
MPLPEEDPQPLLKDQEETAYDSDGKVLSFGIDYDTESGGSTVVPSFSWRKLWLFTGPGFLMCIAFLDP
GNLEGDLQAGAIAGYSLLWLLLWATAMGLLVQLLSARLGVATGRHLAELCREEYPTWARMILWIM
AELALIGADIQEVIGSAIAIQILSNGVLPLWAGVIITASDCFIFLFLENYGVRKLEAAFGILIGIMAVTFA

WMFADAKPSAPELFLGILIPKLSSKTIKQAVGVVGCIIMPHNVFLHSALVQSREIDHNKKGQVQEALR
YYSIESTAALAISFMINLFVTTVFAKGFHGTELANSIGLVNAGQYLQDKYGGGFFPILYIWGIGLLAAG
QSSTITGTYAGQFIMGGFLNLGLKKWLRALITRSCAIIPTIIVALVFDTSEDSLDVLNEWLNMLQSIQIP
FALIPLLCLVSKEQIMGTFTVGPILQMVSWLVAALVMLINGYLLLDFFSNEVTGVVFTTVVCAFTGAY
VTFIIYLISREVTISTWYCPT*

>P. ussuriensis_NRAMP3.2
MPVEENHQPLLQEEEERAYDSDEKVLIIGVDSDTESGGSTVLPPFSWKKLWLFTGPGFLMSIAFLDPG
NLEGDLQAGAIAGYSLLWLLLWATAMGLLVQLLSARLGVATGRHLAELCREEYPTWASMVLWIMA
ELALIGADIQEVIGSAIAIKILSNGFVPLWAGVTITACDCFIFLFLENYGVRKLEAVFAVLIGIMAVTFG
WMFADAKPSASELFLGILIPKLSSRTIQQAVGVVGCIIMPHNVFLHSALVQSREIDHNKKDRVQEALR
YYSIESTTALVISFVINLFVTTVFAKGFYGTELANSIGLVNAGQYLQDKYGGGFFPILYIWGIGLLAAG
QSSTITGTYAGQFIMGGFLNLRLKKWLRALITRSCAIIPTMIVALVFDTSEDSLDVLNEWLNVLQSIQIP
FALIPLLCLVSKEQIMGTFKIGPILKMVAWLVAALVMVINGYLLLDFFFNEVTGVAFTTVVCGFTGAY
AAFIIYLISRGFTCFSRCCPSKQIEVE*

>P. nigra_NRAMP3.1
MPVPEEDPQPLLKDQEETAYDSDGKVLSFGIDYDTESGGSTVVPSFSWRKLWLFTGPGFLMCIAFLDP
GNLEGDLQAGAIAGYSLLWLLLWATAMGLLVQLLSARLGVATGRHLAELCREEYPTWARMILWIM
AELALIGADIQEVIGSAIAIQILSNGVLPLWAGVIITASDCFIFLFLENYGVRKLEAAFGILIGIMAVTFA
WMFADAKPSAPELFLGILIPKLSSKTIKQAVGVVGCIIMPHNVFLHSALVQSREIDHNKKGQVQEALR
YYSIESTAALAISFMINLFVTTVFAKGFHGTELANSIGLVNAGQYLQDKYGGGFFPILYIWGIGLLAAG
QSSTITGTYAGQFIMGGFLNLGLKKWLRALITRSCAIIPTIIVALVFDTSEDSLDVLNEWLNMLQSIQIP
FALIPLLCLVSKEQIMGTFTVGPILQMVSWLVAALVMLINGYLLLDFFSNEVTGVAFTTVVCAFTGAY
VAFIIYLISREVTISTWYCPT*

>P. nigra_NRAMP3.2
MPVEENHQPLLQEEEERAYDSDEKVLIIGVDSDTESGSSTVLPPFSWKKLWLFTGPGFLMSIAFLDPG
NLEGDLQAGAIAGYSLLWLLLWATAMGLLVQLLSARLGVATGRHLAELCREEYPTWASMVLWIMA
ELALIGADIQEVIGSAIAIKILSNGFVPLWAGVTITACDWFIFLFLENYGVRKLEAVFAVLIGIMAVTFG
WMFADAKPSASELFLGILIPKLSSRTIQQAVGVVGCIIMPHNVFLHSALVQSREIDHNKKDRVQEALR
YYSIESTTALVISFVINLFVTTVFAKGFYGTELANSIGLVNAGQYLQDKYGGGFFPILYIWGIGLLAAG
QSSTITGTYAGQFIMGGFLNLRLKKWLRALITRSCAIIPTMIVALVFDTSEDSLDVLNEWLNVLQSIQIP
FALIPLLCLVSKEQIMGTFKIGPTLQMVSWLVAALVMVINGYLLLDFFFNEVTGVAFTTVVCGFTGA
YVAFIIYLISRGFTCFSRCCSSKQIEVE*

>P. deltoides_NRAMP3.1
MPLPEEDPQPLLKDQEETAYDSDGKVLSFGIDYDTESGSSTVVPSFSWRKLWLFTGPGFLMCIAFLDP
GNLEGDLQAGAIAGYSLLWLLLWATAMGLLVQLLSARLGVATGRHLAELCREEYPTWARMILWIM
AELALIGADIQEVIGSAIAIQILSNGVLPLWAGVIITASDCFIFLFLENYGVRKLEAAFGILIGIMAVTFA
WMFADAKPSAPELFLGILIPKLSSKTIKQAVGVVGCIIMPHNVFLHSALVQSREIDHNKKGQVQEALR
YYSIESTAALAISFMINLFVTTVFAKGFHGTELANSIGLVNAGQYLQDKYGGGFFPILYIWGIGLLAAG
QSSTITGTYAGQFIMGGFLNLGLKKWLRALITRSCAIIPTIIVALVFDTSEDSLDVLNEWLNMLQSIQIP
FALIPLLCLVSKEQIMGTFTVGPILQMVSWLVAALVMLINGYLLLDFFSNEVTGVVFTTVVCAFTGAY
VTFIIYLISREVTISTWYCPT*

>P. deltoides_NRAMP3.2
LQAGAIAGYSLLWLLLWATAMGLLVQLLSARLGVATGRHLAELCREEYPTWASMVLWIMAELALI
GADIQEVIGSAIAIKILSNGFVPLWAGVTITACDCFIFLFLENYGVRKLEAVFAVLIGIMAVTFGWMFA
DAKPSASELFLGILIPKLSSRTIQQAVGVVGCIIMPHNVFLHSALVQSREIDHNKKGRVQEALRYYSIES
TTALVISFVINLFVTTVFAKGFYGTELANSIGLVNAGQYLQDKYGGGFFPILYIWGIGLLAAGQSSTIT
GTYAGQFIMGGFLNLRLKKWLRALITRSCAIIPTMIVALVFDTSEDSLDVLNEWLNVLQSIQIPFALIPL
LCLVSKEQIMGTFKIGPILQMVAWLVAALVMVINGYLLLDFFFNEVTGVAFTTVVCGFTGAYAAFIIY
LISRGFTCFSRCCPSKQIEVE*

>S. purpurea_NRAMP3
MSLDENQQPLLQEEEERAYDSDEKVLVIGIDSDAESGGTVLPPFSWKKLWLFTGPGFLMSIAFLDPGN
LEGDLQAGAIAGYSLLWLLFWATAMGLLVQLLSARLGVATGRHLAELCREEYPTWARMILWIMAE
LALIGADIQEVIGSAIAIKILSNGVVPLWAGVTITACDCFIFLFLENYGVRKLEAVFAVLIGVMAVTFG
WMFADAKPSASELFLGILIPKLSSRTIQQAVGVVGCIIMPHNVFLHSALVQSREIDHNNKVQVQEALR
YYSIESTTALVISFVINLFVTTVFAKGFYGTELANSIGLVNAGQYLQDKYGGGFFPILYIWGIGLLAAG
QSSTITGTYAGQFIMGGFLNMRLKKWLRALITRSCAIIPTIIVALIFDTSEDSLDVLNEWLNVLQSIQIPF
ALIPLLCLVSKERIMGTFKIGSILKVVSWLVAALVIVINGYLLLDFFFNEVTGVAFTTVVCTFTAAYAA
FIIYLTSRGVTCSSWRGPPKQIEVE*

>S. brachista_NRAMP3
MSLDENQQPLLQEEEERAYDSDQKVLVIGIDSDAESGGTVLPPFSWKKLWLFTGPGFLMSIAFLDPGN
LEGDLQAGAIAGYSLLWLLLWATAMGLLVQLLSARLGVATGRHLAELCREEYPTWARMILWIMAE
LALIGADIQEVIGSAIAIKILSNGVVPLWAGVTITACDCFIFLFLENYGVRKLEAVFAVLIGVMAVTFG
MMFADAKPSASELFLGILIPKLSSRTIQQAVGVVGCIIMPHNVFLHSALVQSREIDHNNKVQVQEALR
YYSIESTTALVISFVINLFVTTVFAQGFYGTELANSIGLVNAGQYLQDKYGGGFFPILYIWGIGLLAAG
QSSTITGTYAGQFIMGGFLNMRLKKWLRALITRSCAIIPTIIVALVFETSEESLDVLNEWLNVLQSIQIPF
ALIPLLCLVSKERIMGTFKIGSILKVVSWLVAALVIIINGYLLLDFFFNEVTGVAYTTAVCTFTAAYAA
FIIYLTSRGVTCSSWRGPPKQNQQIEVE*

>S. suchowensis_NRAMP3
MSLDENQQPLLQEEEERAYDSDEKVLVIGIDSDAESGRTVLPPFSWKKLWLFTGPGFLMSIAFLDPGN
LEGDLQAGAIAGYSLLWLLLWATAMGLLVQLLSARLGVATGRHLAELCREEYPTWARMILWIMAE
LALIGADIQEVIGSAIAIKILSNGVVPLWAGVTITACDCFIFLFLENYGVRKLEAVFAVLIGVMAVTFG
WMFADAKPSASELFLGILIPKLSSRTIQQAVGVVGCIIMPHNVFLHSALVQSREIDHNNKVQVQEALR
YYSIESTTALVISFVINLFVTTVFAKGFYGTELANSIGLVNAGQYLQDKYGGGFFPILYIWGIGLLAAG
QSSTITGTYAGQFIMGGFLNMRLKKWLRALITRSCAIIPTIIVALIFDTSEDSLDVLNEWLNVLQSIQIPF
ALIPLLCLVSKERIMGTFKIGSILKVVSWLVAALVIVINGYLLLDFFFNEVTGVAFTTAVCTFTAAYAA
FIIYLTSRGITCSSWRGPPKQIEAE*

>S. eriocephala_NRAMP3
MSLDENQQALLQEEEERAYDSDEKVLVIGVYSDAESGGTVLPPFSWKKLWLFTGPGFLMSIAFLDPG
NLEGDLQAGAIAGYSLLWLLLWATAMGLLVQLLSARLGVATGRHLAELCREEYPTWARMILWIMA
ELALIGADIQEVIGSAIAIKILSNGVVPLWAGVTITACDCFIFLFLENYGVRKLEAVFAVLIGVMAVTFG
WMFADAKPSASELFLGILIPKLSSRTIQQAVGVVGCIIMPHNVFLHSALVQSREIDHNNKIQVQEALRY
YSIESTTALVISFVINLFVTTVFAKGFYGTELANSIGLVNAGQYLQDKYGGGFFPILYIWGIGLLAAGQ
SSTITGTYAGQFIMGGFLNMRLKKWIRALITRSCAIIPTIIVALIFDTSEDSLDVLNEWLNVLQSIQIPFA
LIPLLCLVSKERIMGTFKIGSILKVVSWLVAALVMVINGYLLLDFFFNEVTGVAFTTAVCTFTAAYAA
FIIYLTSRGVTCSSWRGPPKQIEVE*

>S. sachalinensis_NRAMP3
MSLDENQQPLLQEEEERAYDSDEKVLVIGIDSDAESGGTVLPPFSWKKLWLFTGPGFLMSIAFLDPGN
LEGDLQAGAIAGYSLLWLLLWATAMGLLVQLLSARLGVATGRHLAELCREEYPTWARMILWIMAE
LALIGADIQEVIGSAIAIKILSNGVVPLWAGVTITACDCFIFLFLENYGVRKLEAVFAVLIGVMAVTFG
WMFADAKPSASELFLGILIPKLSSRTIQQAVGVVGCIIMPHNVFLHSALVQSREIDHNNKVQVQEALR
YYSIESTTALVISFVINLFVTTVFAKGFYGTELANSIGLVNAGQYLQDKYGGGFFPILYIWGIGLLAAG
QSSTITGTYAGQFIMGGFLNMRLKKWLRALITRSCAIIPTIIVALIFDTSEDSLDVLNEWLNVLQSIQIPF
ALIPLLCLVSKERIMGTFKIGSILKVVSWLVAALVIVINGYLLLDFFFNEVIGVAFTTAVCTFTAAYAA
FIIYLTSRGITCSSWRGPPKQIEAE*

>S. dasyclados_NRAMP3[$]
MAVTFGWMFADAKPSASELFLGILIPKLSSRTIQQAVGVVGCIIMPHNVFLHSALVQSREIDHNNKVQ
VQEAVRYYSIESTTALVISFVINLFVTTVFAQGFYGTELANSIGLVNAGQYLQDKYGGGFFPILYIWGI
GLLAAGQSSTITGTYAGQFIMGGFLNMRLKKWLRALITRSCAIIPTIIVALIFDTSEDSLDVLNEWLNV

LQSIQIPFALIPLLCLVSKERIMGTFKIGSILKVVSWLVAALVIVINGYLLLDFFFNEVTGVAFTTAVCTF TAAYAAFIIYLTSRGITCSSWRGPPKQIEVE*

>S. fargesii_NRAMP3$
MSIAFLDPGNLEGDLQAGAIAGYSLLWLLLWATAMGLLVQLLSARLGVATGRHLAELCREEYPTWA RMILWIMAELALIGADIQEVIGSAIAIKILSNGVVPLWAGVTITACDCFIFLFLENYGVRKLEAVFAVLI GVMAVTFGMMFADAKPSASELFLGILIPKLSSRTIQQAVGVVGCIIMPHNVFLHSALVQSREIDHNNK VQVQEALRYYSIESTTALVISFVINLFVTTVFAQGFYGTELANSIGLVNAGQYLQDKYGGGFFPILYIW GIGLLAAGQSSTITGTYAGQFIMGGFLNMRLKKWLRALITRSCAIIPTIIVALVFETSEESLDVLNEWLN VLQSIQIPFALIPLLCLVSKERIMGTFKIGSILKVVSWLVAALVIIINGYLLLDFFFNEVTGVAYTTAVCT FTAAYAAFIIYLTSRGVTCSSWRGPPKQNQQIEVE*

>S. viminalis_NRAMP3$
MSLDENQQPLLQEEEERAYDSDEKVLVIGIDSDAESSGTVLPPFSWKKLWLFTGPGFLMSIAFLDPGN LEGDLQAGAIAGYSLLWLLLWATAMGLLVQLLSARLGVATGRHLAELCREEYPTWARMILWIMAE LALIGADIQEVIGSAIAIKILSNGVVPLWAGVTITACDCFIFLFLENYGVRKLEAVFAVLIGVMAVTFG WMFADAKPSASELFLGILIPKLSSRTIQQAVGVVGCIIMPHNVFLHSALVQSREIDHNNKVQVQEAVR YYSIESTTALVISFVINLFVTTVFAQGFYGTELANSIGLVNAGQYLQDKYGGGFFPILYIWGIGLLAAG QSSTITGTYAGQFIMGGFLNMRLKKWLRALITRSCAIIPTIIVALIFDTSEDSLDVLNEWLNVLQSIQIPF ALIPLLCLVSKERIMGTFKIGSILKVVSWLVAALVIVINGYLLLDFFF

Supplementary materials and methods

Gene expression

Total RNA from 1-month-old poplars or 1 week-old *A. thaliana* grown *in vitro* on half strength Murashige and Skoog (MS) or ABIS medium, respectively, was extracted using RNeasy Plant Mini Kit (Qiagen) as previously described (Pottier et al., 2015a). Five micrograms of DNA-free RNA were used for reverse transcription by the SuperScript III First-Strand kit (Invitrogen) using random hexamers. Primers were designed using OligoPerfect™ Designer (http://tools.lifetechnologies.com) and their specificity was confirmed by analysis of the melting curves and sequencing of the PCR products. qPCR reactions were performed on a Roche LightCycler 96 using primers listed in supplementary table S7 and Roche reagents, according to the manufacturer's instructions (http://www.roche.com). Relative transcript levels were calculated by normalization to the transcript amount of constitutively expressed genes.

Yeast strains, transformations and media

DEY1453 (*fet3fet4*, (Eide et al. 1996), *smf1* (Supek et al. 1996; Thomine et al. 2000) and *smf2* (Cohen et al. 2000) yeast strains were grown at 30ºC on Yeast extract/Peptone/Dextrose (before transformation) or synthetic dextrose -ura (after transformation). For the DEY1453 strain, media were supplemented with 0.2 mM $FeCl_3$. Yeast cells were transformed according to standard procedures (Invitrogen).

Poplar transformation and regeneration

The poplar INRA 717-1-B4 clone (*P. tremula* x *P. alba*) was transformed as indicated in supplementary material and methods and using media listed in Supplementary table S8 (Leplé et al. 1992). Briefly, stem explants were excised from *in vitro* grown poplar and incubated for 2 days in the dark on M1 medium. The explants were then co-cultivated for 16 h at 24°C on an orbital shaker (180 rpm) with 150 ml of a suspension (OD_{600} = 0.3) of *A. tumefaciens* C58/pMP90 transformed with pMDC83 constructs. The explants were washed 5 times in sterile water under orbital shaking (140 rpm). After washing, they were transferred on M2 medium containing Ticarpen, cefotaxime and hygromycin B, and incubated at 24°C in darkness for 21 days. The plates were subsequently transferred to light (16 h 130 µE.m^{-2}/8h dark) and generated green calli were transferred to M3 agar medium with hygromycin B (Supplementary table S8). The regenerated transformed shoots were then transferred on half strength MS agar medium with hygromycin to allow root regeneration. The transgenic lines were propagated on the same medium until transfer to pots in the greenhouse.

References

Chen J, Huang Y, Brachi B, Yun Q, Zhang W, Lu W, Li H, Li W, Sun X, Wang G, et al. 2019. Genome-wide analysis of Cushion willow provides insights into alpine plant divergence in a biodiversity hotspot. *Nat. Commun.* 10:5230.

Cohen A, Nelson H, Nelson N. 2000. The family of SMF metal ion transporters in yeast cells. *J. Biol. Chem.* 275:33388–33394.

Dai X, Hu Q, Cai Q, Feng K, Ye N, Tuskan GA, Milne R, Chen Y, Wan Z, Wang Z, et al. 2014. The willow genome and divergent evolution from poplar after the common genome duplication. *Cell Res.* 24:1274–1277.

Eide D, Broderius M, Fett J, Guerinot ML. 1996. A novel iron-regulated metal transporter from plants identified by functional expression in yeast. *Proc. Natl. Acad. Sci.* 93:5624–5628.

Goodstein DM, Shu S, Howson R, Neupane R, Hayes RD, Fazo J, Mitros T, Dirks W, Hellsten U, Putnam N, et al. 2012. Phytozome: a comparative platform for green plant genomics. *Nucleic Acids Res.* 40:D1178–D1186.

Kosakovsky Pond SL, Frost SDW. 2005. Not So Different After All: A Comparison of Methods for Detecting Amino Acid Sites Under Selection. *Mol. Biol. Evol.* 22:1208–1222.

Lamesch P, Berardini TZ, Li D, Swarbreck D, Wilks C, Sasidharan R, Muller R, Dreher K, Alexander DL, Garcia-Hernandez M, et al. 2012. The Arabidopsis Information Resource (TAIR): improved gene annotation and new tools. *Nucleic Acids Res.* 40:D1202–D1210.

Leplé J-C, Brasileiro A-C, Michel M-F, Delmotte F, Jouanin L. 1992. Transgenic poplars: expression of chimeric genes using four different constructs. *Plant Cell Rep.* [Internet] 11. Available from: http://link.springer.com/10.1007/BF00232166

Lin Y-C, Wang J, Delhomme N, Schiffthaler B, Sundström G, Zuccolo A, Nystedt B, Hvidsten TR, de la Torre A, Cossu RM, et al. 2018. Functional and evolutionary genomic inferences in *Populus* through genome and population sequencing of American and European aspen. *Proc. Natl. Acad. Sci.* 115:E10970–E10978.

One Thousand Plant Transcriptomes Initiative. 2019. One thousand plant transcriptomes and the phylogenomics of green plants. *Nature* 574:679–685.

Sayers EW, Beck J, Bolton EE, Bourexis D, Brister JR, Canese K, Comeau DC, Funk K, Kim S, Klimke W, et al. 2021. Database resources of the National Center for Biotechnology Information. *Nucleic Acids Res.* 49:D10–D17.

Sjödin A, Street NR, Sandberg G, Gustafsson P, Jansson S. 2009. The *Populus* Genome Integrative Explorer (PopGenIE): a new resource for exploring the *Populus* genome. *New Phytol.* 182:1013–1025.

Supek F, Supekova L, Nelson H, Nelson N. 1996. A yeast manganese transporter related to the macrophage protein involved in conferring resistance to mycobacteria. *Proc. Natl. Acad. Sci.* 93:5105–5110.

Thomine S, Wang R, Ward JM, Crawford NM, Schroeder JI. 2000. Cadmium and iron transport by members of a plant metal transporter family in Arabidopsis with homology to Nramp genes. *Proc. Natl. Acad. Sci.* 97:4991–4996.

Zhang B, Zhu W, Diao S, Wu X, Lu J, Ding C, Su X. 2019. The poplar pangenome provides insights into the evolutionary history of the genus. *Commun. Biol.* 2:215.

Zhou R, Macaya-Sanz D, Rodgers-Melnick E, Carlson CH, Gouker FE, Evans LM, Schmutz J, Jenkins JW, Yan J, Tuskan GA, et al. 2018. Characterization of a large sex determination region in Salix purpurea L. (Salicaceae). *Mol. Genet. Genomics* 293:1437–1452.
