# Supplementary material for: Duplication of NRAMP3 Gene in Poplars Generated Two Homologous Transporters with Distinct Functions
Source: Mol Biol Evol. 2022 Jun 14;39(6):msac129. doi: 10.1093/molbev/msac129 (PMC9234761; doi:10.1093/molbev/msac129)
Supplement: msac129_Supplementary_Data [file msac129_supplementary_data.zip › Pottier,_Le_Thi_et_al_uncropped_pictures_of_yeast_drop_test.pdf]

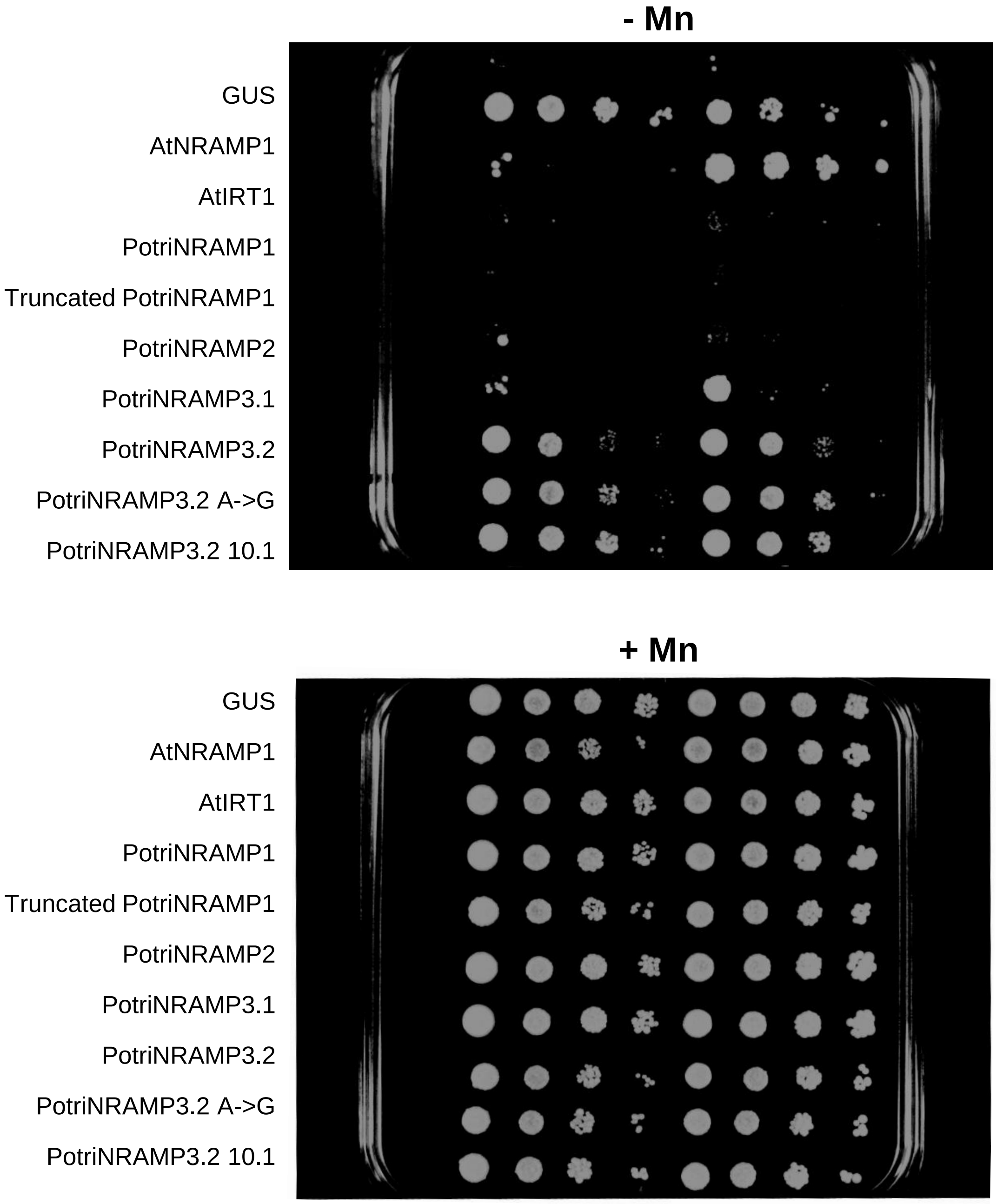

Fig. A1. Functional complementation of the *smf1* yeast Mn uptake mutant. Yeast cells were transformed with pDR195gtw vector containing the cDNA of *GUS, AtNRAMP1, AtIRT1, PotriNRAMP1 (2 variants), PotriNRAMP2*, *PotriNRAMP3.1* and *PotriNRAMP3.2 (3 variants)*. Transformed *smf1* yeasts were grown overnight in liquid synthetic dextrose -ura. The cultures were diluted to ODs of 1 to 10^{-3} and spotted on synthetic dextrose -ura plates. Transformed *smf1* yeasts were spotted on medium supplemented with 5 mM EGTA and 100 µM $MnSO_4$ (+ Mn) or with 5 mM EGTA without $MnSO_4$ (- Mn). The plates were incubated at 30°C for 5 days (*smf1*) before photography. The right and left part of each plate show results obtained with 2 different transformants per construct.

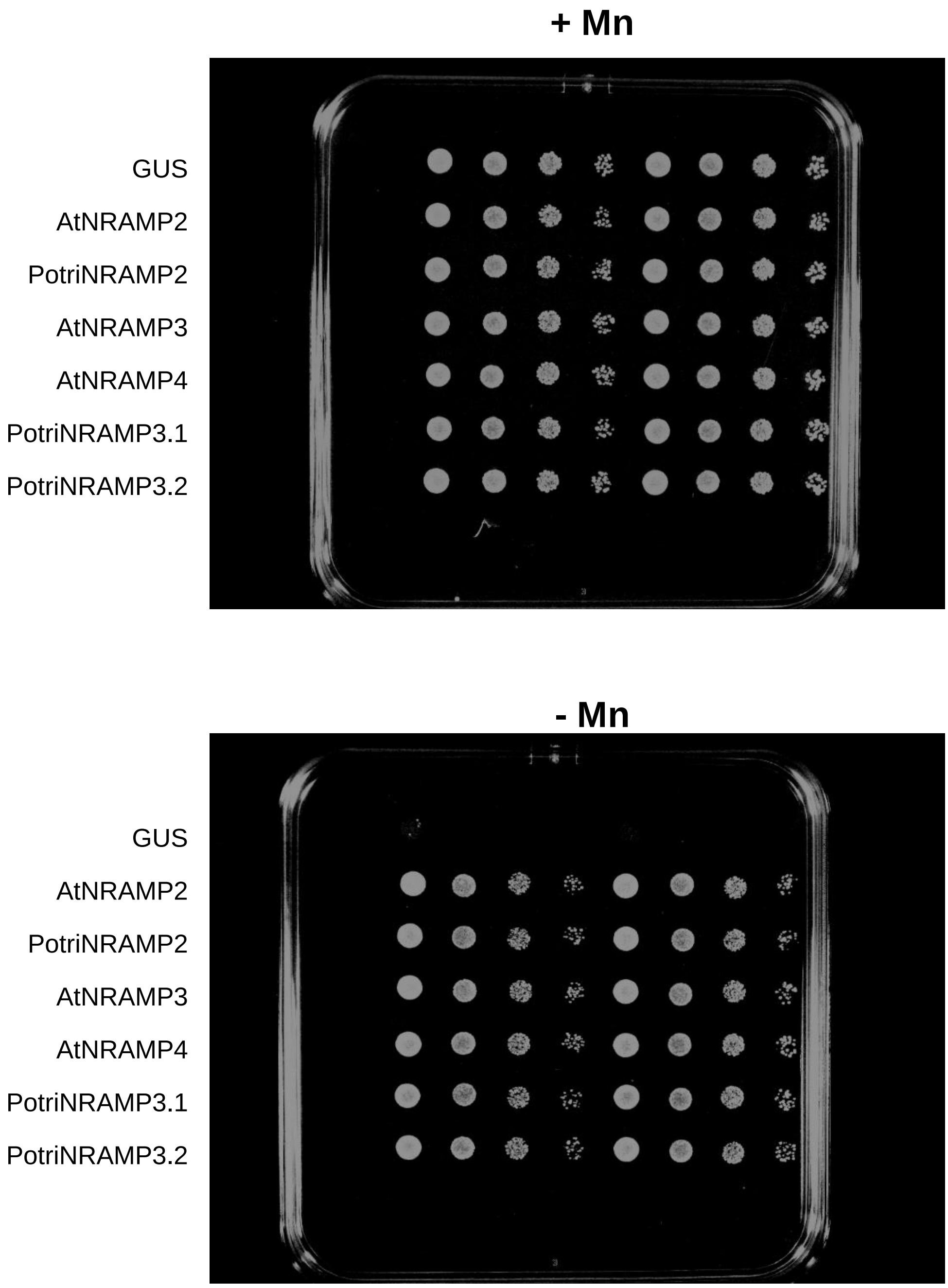

Fig. A2. Functional complementation of the *smf2* yeast mutant deficient of the intracellular Mn distribution. Yeast cells were transformed with pDR195gtw vector containing the cDNA of *GUS, AtNRAMP2*, *PotriNRAMP2, AtNRAMP3, AtNRAMP4*, *PotriNRAMP3.1* and *PotriNRAMP3.2*. Transformed *smf2* yeasts were grown overnight in liquid synthetic dextrose -ura. The cultures were diluted to ODs of 1 to 10^{-3} and spotted on synthetic dextrose -ura plates. Transformed *smf2* yeasts were spotted on media medium supplemented with 10 mM EGTA and 100 µM $MnSO_4$ (+ Mn) or with 5 mM EGTA without $MnSO_4$ (- Mn). The plates were incubated at 30°C for 2 days before photography. The right and left part of each plate show results obtained with 2 different transformants per construct.

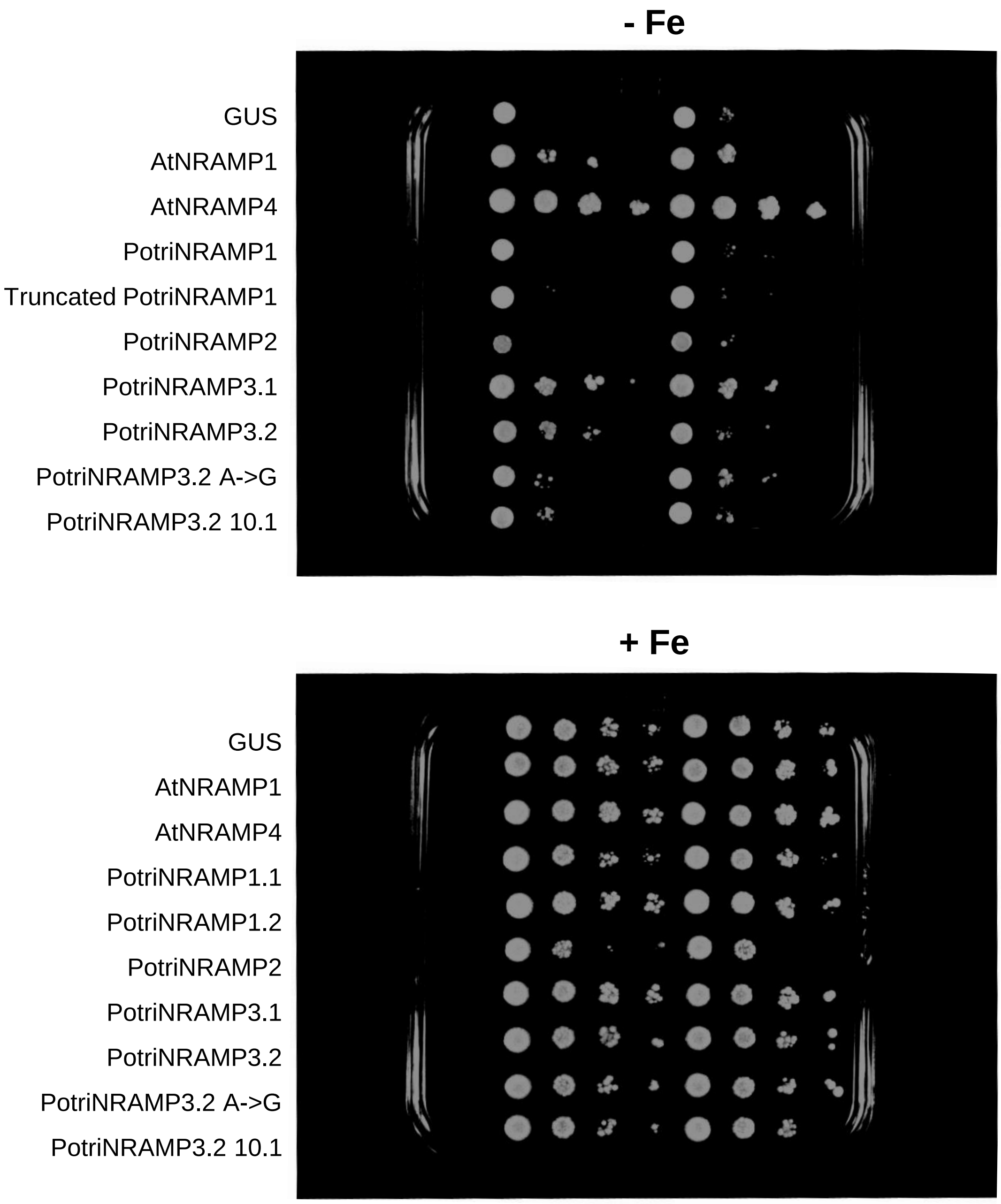

Fig. A3. Functional complementation of the *fet3fet4* yeast iron uptake mutant. *fet3fet4* yeast cells were transformed with pDR195gtw vector containing the cDNA of *GUS, AtNRAMP1, AtNRAMP4*, *PotriNRAMP1* (2 variants), *PotriNRAMP2*, *PotriNRAMP3.1* or *PotriNRAMP3.2* (3 variants). Transformed *fet3fet4* were grown overnight in liquid synthetic dextrose -ura supplemented with 0.2 mM $FeCl_3$. Cultures from 2 independent transformed strains for each construct (left-right) were diluted to ODs of 1 to 10^{-3} and spotted on synthetic dextrose -ura plates supplemented with 100 µM of the Fe chelator BathoPhenanthroline-Di-Sulfonic acid (BPDS) and 0.2 mM $FeCl_3$ (+ Fe) or with 100 µM BPDS and 40 µM $FeCl_3$ (- Fe). The plates were incubated at 30°C for 4 days before photography.
